# Indole-containing arene-ruthenium complexes with broad spectrum activity against antibiotic-resistant bacteria

**DOI:** 10.1016/j.crmicr.2021.100099

**Published:** 2021-12-16

**Authors:** Victoria C. Nolan, Laia Rafols, James Harrison, Joan J. Soldevila-Barreda, Marialuisa Crosatti, Natalie J. Garton, Malgorzata Wegrzyn, Danielle L. Timms, Colin C. Seaton, Helen Sendron, Maria Azmanova, Nicolas P.E. Barry, Anaïs Pitto-Barry, Jonathan A.G. Cox

**Affiliations:** 1 College of Health and Life Sciences, Aston University, B4 7ET, Birmingham, United Kingdom; 2 School of Chemistry and Biosciences, University of Bradford, BD7 1DP, Bradford, United Kingdom; 3 CL3 facility, Division of Biomedical Services, University of Leicester, LE1 7RH, Leicester, United Kingdom; 4 Department of Respiratory Sciences and Leicester TB Research Group, University of Leicester, LE1 7RH, Leicester, United Kingdom; 5 Université Paris-Saclay, CNRS, Institut Galien Paris-Saclay, 92296 Châtenay-Malabry, France

## Abstract

Antimicrobial resistant (AMR) bacteria are emerging and spreading globally, threatening our ability to treat common infectious diseases. The development of new classes of antibiotics able to kill or inhibit the growth of such AMR bacteria through novel mechanisms of action is therefore urgently needed. Here, a new family of indole-containing arene ruthenium organometallic compounds are screened against several bacterial species and drug resistant strains. The most active complex [(*p*-cym)Ru(O-cyclohexyl-*1H*-indole-2-carbothioate)Cl] (**3**) shows growth inhibition and bactericidal activity against different organisms (*Acinetobacter baumannii, Mycobacterium abscessus, Mycobacterium tuberculosis, Staphylococcus aureus, Salmonella enterica* serovar Typhi and *Escherichia coli*), demonstrating broad-spectrum inhibitory activity. Importantly, this compound series exhibits low toxicity against human cells. Owing to the novelty of the antibiotic family, their moderate cytotoxicity, and their inhibitory activity against Gram positive, Gram negative and acid-fast, antibiotic resistant microorganisms, this series shows significant promise for further development.

## Highlights

• A new family of indole-containing arene ruthenium organometallic compounds are active against several bacterial species and drug resistant strains
• Bactericidal activity observed against various Gram negative, Gram positive and acid-fast bacteria, demonstrating broad-spectrum inhibitory activity
• Compound series exhibits low toxicity against human cells
• Shows considerable promise as next generation antibiotics

## Introduction

Antibiotic resistance is a worldwide threat to public health, food security, and economic and societal developments. (1) The prevalence of antimicrobial resistance (AMR) among common pathogens is rapidly increasing, which leads to widespread diseases becoming harder, or impossible, to cure. AMR organisms of interest include *Acinetobacter baumannii, Escherichia coli, Salmonella sp.* and several other multidrug-resistant Gram-negative organisms. (Wang et al., 2019) In the last decades, a global effort has allowed the development of new antibiotic drugs with activity against common AMR organisms, (Cox and Worthington, 2017) but resistance against such agents is already emerging. There is therefore an urgent need to find new families of compounds with high levels of antibacterial activity, novel mechanisms of action and low frequencies of antibiotic resistance.

Inorganic metallodrugs offer potential for unique mechanisms of drug action based on the choice of the metal, its oxidation state, the types and number of coordinated ligands and the coordination geometry. (Barry and Sadler, 2013, Barry and Sadler, 2014) As such, medicinal inorganic chemistry provides a rich platform in the pharmacological space for structural and electronic diversity. (Silva et al., 2021) Medicinal inorganic chemistry has been stimulated by the success of platinum anticancer drugs (used as a component of nearly 50% of all cancer chemotherapy treatments), by the use of gadolinium(III) complexes as MRI contrast agents (about 20 million doses administered per year), and of the radionuclide 99m-technetium radiopharmaceuticals for γ-ray imaging (used in about 20 million radio diagnostic procedures each year). (Biancalana et al., 2020, (Farley et al., 2021), Hanif et al., 2020, Rafols et al., 2021) However, the involvement of metals in many other diseases and conditions is of current interest in relation to their causes, their treatment or detection, including neurodegeneration, (Anthony et al., 2020) fungal (Golbaghi et al., 2020), parasitic, (Minori et al., 2020, Melis et al., 2021) viral (Yuan et al., 2020) infections and inflammation. (Zhang et al., 2017) In the last decades, a wide range of inorganic and organometallic compounds have been investigated for killing or inhibiting microbial growth. (Gambino and Otero, 2019, Sierra et al., 2019, Ravera et al., 2018, Jürgens and Casini, 2017, Lee et al., 2017, Patil et al., 2015, Patra et al., 2012, Patra et al., 2015, Smitten et al., 2020, Smitten et al., 2020, Sierra et al., 2019, Frei, 2020, Nasiri Sovari and Zobi, 2020, Frei et al., 2020, Frei et al., 2021, Sovari et al., 2021, Sovari et al., 2020)

The antimicrobial activity of some half-sandwich “piano-stool” complexes of precious metals (Ru, Os, Rh, Ir) has very recently proven to be highly promising in our current fight against AMR, with demonstrated potency against drug-resistant strains of *Mycobacterium tuberculosis*. (Coverdale et al., 2021) Half-sandwich complexes are versatile and highly modifiable organometallics, which contains a π-bonded neutral arene or negatively charged cyclopentadienyl ligand, and, usually, one monodentate and one bidentate ligand. The monodentate and bidentate ligands allow further structural and electronic control of reactivity, including the incorporation of bio-active moieties.

We have recently developed an interest in investigating the effect of bio-active indole moieties as bidentate ligands on the biological properties of half-sandwich metal complexes. (Soldevila-Barreda et al., 2020) Indoles are bicyclic heterocycles that are commonly found in plants, bacteria and animals. Natural and synthetic indole-based compounds have been widely used as antibacterial, antifungal, anti-inflammatory, antihistaminic, and anticancer drugs. (Wan et al., 2019, Dadashpour and Emami, 2018) Examples of such compounds currently in clinical use are the non-steroidal anti-inflammatory drug indomethacin (Lal and Snape, 2012) or the antiretroviral delavirdine. (Xu and Lv, 2009)

**Scheme 1:**
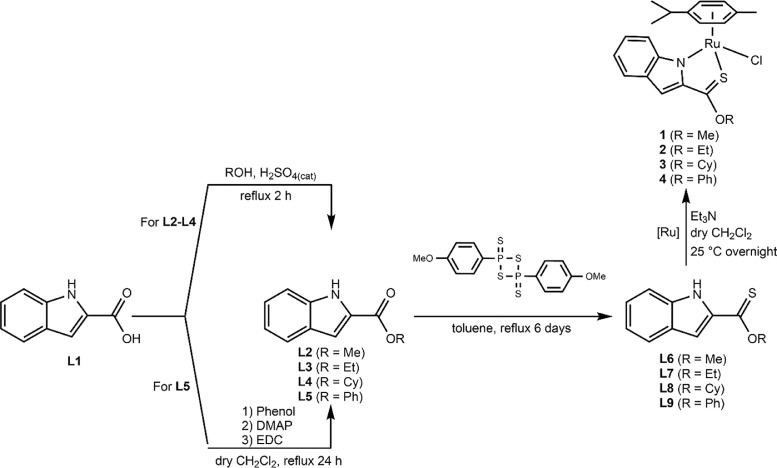
Preparation of the indole-based ligands **L6** – **L9** and complexes **1 – 4**.

Herein, we report the synthesis and characterisation of a new family of four ruthenium half-sandwich complexes containing O-R-*1H*-indole-2-carbothioate (O-R-ind-th; R = methyl, ethyl, cyclohexyl, phenyl) [(*p*-cym)Ru(O-Me-ind-th)Cl] (**1**), [(*p*-cym)Ru(O-Et-ind-th)Cl] (**2**), [(*p*-cym)Ru(O-Cy-ind-th)Cl] (**3**), [(*p*-cym)Ru(O-Ph-ind-th)Cl] (**4**) (Scheme 1). Their stability in solution, hydrolysis rates and acid dissociation constants are studied, along with toxicity data on three human cell lines for both ligands and complexes. The bactericidal activities of the four ligands and complexes against several drug resistant isolates of *Escherichia coli* are reported, including(*E. coli J53 2138*, a clinical isolate producing the extended spectrum β-lactamase (ESBL) OXA-1, which confers resistance to ampicillin, ticarcillin, piperacillin and cephalosporins, and *E. coli J53 2140E*, producing the ESBL OXA-3.

## Results and discussions

### Synthesis, stability in solution, aquation and pKa determination

**Fig. 1:**
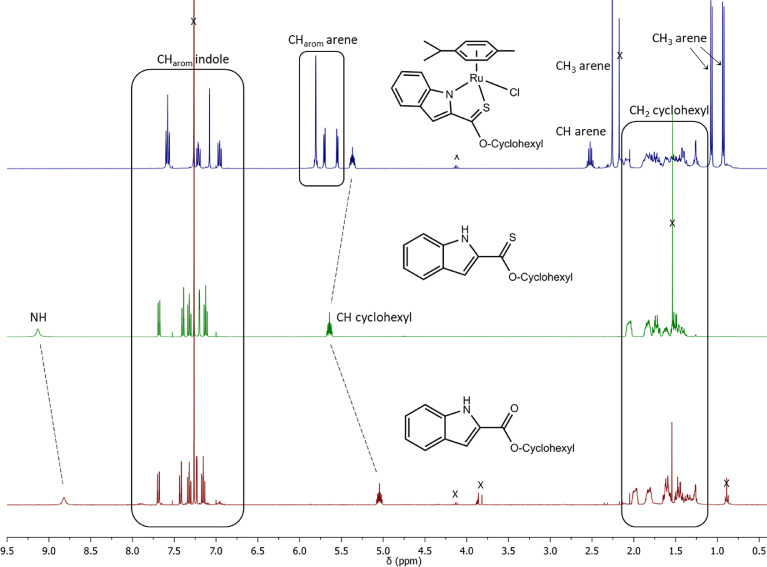
^1^H NMR spectra of **L4** (bottom), **L8** (middle), and complex **3** (top) in CDCl_3_ (400 MHz). Residual solvents are marked with a cross.

The indole 2-carboxylate ligands **L2** – **L5** were synthesised by esterification of *1H*-indole-2-carboxylic acid (L1) with the corresponding alcohol (MeOH, EtOH, CyOH, PhOH, respectively). Thiolation of the carboxylic group of ligands **L2** – **L5** was performed by refluxing the corresponding ligands with the Lawesson's reagent in toluene to yield the O-R-*1H*-indole-2-carbothioate ligands **L6** – **L9** (R = methyl, ethyl, cyclohexane, benzene; Scheme 1; Experimental Section). Complexes **1 – 4** were then prepared by stirring the dichloro(*p*-cymene)ruthenium(II) dimer with the corresponding ligand (**L6** – **L9**) in dry dichloromethane at ambient temperature and in the presence of triethylamine. All ligands and complexes were characterised by ^1^H and ^13^C NMR spectroscopy, and high-resolution ESI-MS (Experimental Section). ^1^H and ^13^C NMR spectra can be found in the Supporting Information (Figs. S1 – S24), as well as the HR mass spectra (Figs. S25 – S36). As an example, Fig. 1 shows the ^1^H NMR spectra of **L4** (bottom), **L8** (middle), and complex **3** (top) in CDCl_3_. The lowest field region (9.5 – 7.0 ppm) shows the aromatic protons of the indole-based substituent, followed by the region of the coordinated arene (5.5 – 4.0 ppm), and by the aliphatic region (4.0 – 0.5 ppm). Chemical shifts for the protons located near the ligand heteroatoms that bind to the metal can be observed, for example, the proton of the CH cyclohexyl bonded to the oxygen/sulfur (Fig. 1) can be seen to shift upfield from the free ligand **L8** to the metal complex **3**. Such shifts can be explained by the stereo-electronic effects due to the coordination to the metal moiety, as previously observed. (Soldevila-Barreda et al., 2020) The signal for the NH proton is also no more visible after complexation, which confirms the successful synthesis of the complex.

**Fig. 2:**
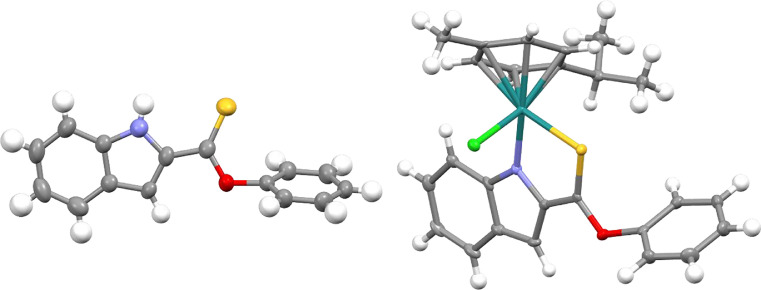
Structures of ligand **L5** (left) and Ru^II^ complex **4** (right). Thermal ellipsoids are drawn at the 50% probability level.

Crystals of ligand **L5** and complexes **2** [(*p*-cym)Ru(O-Et-ind-th)Cl], **3** [(*p*-cym)Ru(O-Cy-ind-th)Cl], and **4** ([(*p*-cym)Ru(O-Ph-ind-th)Cl], suitable for X-ray structure determination, were obtained by slow diffusion of hexane into a saturated dichloromethane solution at 20 ºC. The crystallographic data and selected bond lengths and angles are given in Tables S1 – S12, and the crystal structures are shown in Fig. 2 and Fig. S40. Complexes **2-4** adopt a pseudo-octahedral structure with Ru^II^ bound to a η^6^-*para*-cymene ring, a *N,S-*chelated indole and chloride as ligands to form an 18-electron complex with “piano-stool” geometry.

**Fig. 3:**
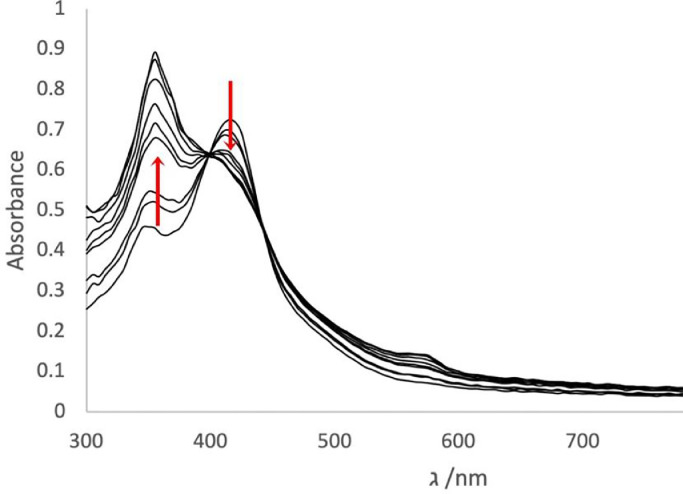
UV−vis spectra of complex **2** in the mixture DMSO/RPMI over time (10^−5^ M, 298 K, 24 h).

To investigate the stability of complexes **1** – **4**, the compounds were dissolved in a mixture (1:1) (v/v) of DMSO/RPMI and UV-Vis spectra were recorded over 24 h (Fig. 3). The absorption band at 420 nm gradually decreases while the one at 350 nm concomitantly increases, intersecting at the isosbestic point, which indicates that only the chloride and the hydrolysed forms of the complex contribute to the observed absorptions. (Bacac et al., 2004, Peacock et al., 2007, Peacock et al., 2007, Scolaro et al., 2008) The time dependence of the absorbance allows for the determination of the rate constants by plotting the absorbance *versus* time at fixed wavelength for each compound (Table 1). The hydrolysis rate depends on the ester group of the indole ligand. Complex **4** (the only complex containing an aromatic R substituent) presents the fastest hydrolysis. The rate of hydrolysis of complexes **1** – **3**, with aliphatic R groups, suggests that the bulkier the R substituent is, the slower the aquation process is.

**Table 1:** Reactivity of complexes **1 – 4**. p*K*a values measured using UV-Vis spectroscopy (10^−5^ M, 298 K), solvent acetonitrile/water v/v. Aquation constants and rates of hydrolysis measured using UV-Vis spectroscopy (10^−5^ M, 298K), in a mixture (1:1) DMSO/RPMI v/v.

Compound	p*K*a	*K*_aquation_ (s^−1^)
[(*p*-cym)Ru(O-Me-ind-th)Cl] (**1**)	10.34 ± 0.07	2.0×10^−4^ ± 6×10^−5^
[(*p*-cym)Ru(O-Et-ind-th)Cl] (**2**)	10.24 ± 0.09	1.8×10^−4^ ± 4×10^−5^
[(*p*-cym)Ru(O-cy-ind-th)Cl] (**3**)	9.81 ± 0.07	1.2×10^−4^ ± 3×10^−5^
[(*p*-cym)Ru(O-ph-ind-th)Cl] (**4**)	10.07 ± 0.05	2.4×10^−4^ ± 4×10^−5^

Aquation of the monodentate ligand X is a common behaviour for half-sandwich complexes of the type [(arene)M(*N*U*S*)X] and is usually considered an activation step, which allows further reactions with the corresponding targets. (Liu and Sadler, 2014, Soldevila-Barreda and Metzler-Nolte, 2019, Meier-Menches et al., 2018, Rilak Simović et al., 2019) Solutions ranging from pH 7 to 12 were prepared using 0.1 M NaOH solution, and complexes **1** – **4** (previously dissolved in pure acetonitrile) were added to the corresponding solution with a known pH and mixed for 10 min. Spectra were recorded by UV-visible spectroscopy. The p*K*a values of the complexes were calculated using Origin 2019 by plotting the absorbance at the corresponding wavelength against the pH and fitting it to the Boltzmann equation to obtain the inflection point. For complexes **1** – **4**, the p*K*a values were calculated to be around 10 (Experimental section and Table 1). These p*K*a values are high, although such decrease in acidity has been previously reported (generally attributed to an increased electron density on the metal centre). (Liu and Sadler, 2014, Peacock et al., 2006, Cross et al., 2016) As a consequence of such high p*K*a values, only the aqua adduct of the metal complexes is expected to be present at physiological pH (7.4), thus favouring the reaction between the metal complexes with possible ligands such as nucleobases or proteins.

### Toxicity studies against human cells

Toxicity data against human cells is important information for the identification of a novel family of antibiotic drug candidates. Initially developed for anticancer activity, complexes **1** – **4** (and ligands **L6 – L9**) were tested against human ovarian adenocarcinoma (A2780), cisplatin-resistant variant of A2780 (A2780cisR) and normal human prostate epithelial (PNT2) cell lines. Half-maximal inhibitory concentrations (IC_50_) were determined using a 24 h MTT assay with 48 h recovery period. The IC_50_ values are shown in table 2 and the IC_50_ graphs for ligands **L6** – **L9** and complexes **1** – **4** against PNT2, A2780, and A2780cisR can be found in the Supporting Information (Figs. S37 – S38).

**Table 2:** IC_50_ values (µM) in A2780, A2780 cisplatin resistant and PNT2 cells for ligands **L6** – **L9** and for complexes **1 – 4**.

	IC_50_ values (µM)		
Compound	A2780	A2780cisR	PNT2
**L6**	>100	>100	>100
**L7**	>100	>100	>100
**L8**	72 ± 2	76 ± 5	71 ± 5
**L9**	21 ± 1	20 ± 2	26 ± 2
**1**	22 ± 1	28 ± 1	32 ± 2
**2**	12 ± 2	22 ± 2	37 ± 2
**3**	10.7 ± 0.6	15.3 ± 0.9	20.8 ± 0.9
**4**	68 ± 4	83 ± 3	96 ± 6
Cisplatin	5.9 ± 0.4	11.8 ± 0.8	26.1 ± 0.6

Complexes **1, 2** and **3** are moderately cytotoxic against the tested cell lines and showed 2 – 3x higher IC_50_ values towards normal prostate cells in comparison to cancer cells. They are also less toxic than cisplatin against all cell lines. Complex **4** exhibits only low toxicity against the three cell lines.

### Antibiotic activity

**Fig. 4:**
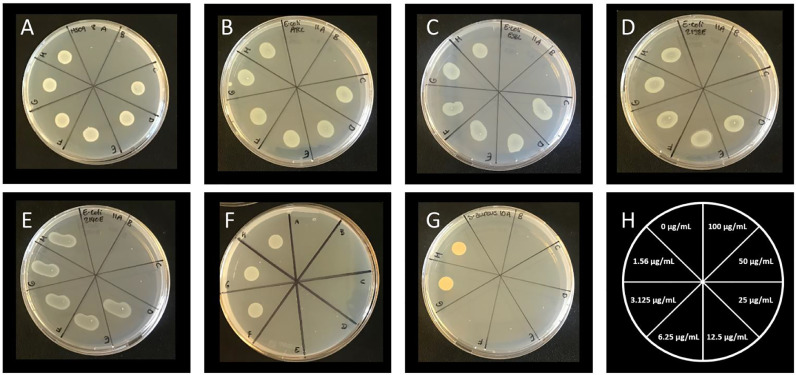
The minimum bactericidal concentrations (MBC) for complex 3 identified by absence of growth. MBCs were defined by minimum concentration at which an absence of bacterial growth was observed after exposure to complex **3**. A, *Acinetobacter baumannii* ATCC 12156 had an MBC of 50 µg/mL (n=3). B, *Escherichia coli* ATCC 11775 and C, *E. coli* ESBL had an MBC of 50 µg/mL (n=3). D, *E. coli* 2138E and E, *E. coli* 2140E both had MBCs of 25 µg/mL (n=3). F, *Salmonella enterica* serovar Typhi had an MBC of 6.25 µg/mL (n=3). G, *Staphylococcus aureus* exhibited an MBC of 3.125 µg/mL (n=3). H, plate map showing concentrations of complex **3**.

A concentration range of complexes **1** – **4** were tested and activity was observed for a range of organisms, including *Mycobacterium abscessus* NCTC 13031*, Escherichia coli* ATCC 11775, I469 ESBL, J53 2138E, J53 2140E*, Staphylococcus aureus* ATCC 29213*, Acinetobacter baumannii* NCTC 12156*, Salmonella enterica* serovar Typhi and *Mycobacterium tuberculosis* H37Rv. The minimum inhibitory concentration (MIC) and minimum bactericidal concentration (MBC) activity of all four complexes were determined (Tables 3 and 4). Overall, complex **3** was the most effective, inhibiting nine out of the 12 organisms tested. Complex **3** also had the lowest MIC observed for all complexes tested, at 1.56 µg/mL against *S. aureus*. Bactericidal activity was also observed against seven out of the 12 organisms tested (Fig. 4). The two organisms that did not have an MBC were both mycobacterial species, *M. abscessus* and *M. tuberculosis* (Fig. 4 and 6). However, complex **3** was bacteriostatic against both species of mycobacteria inhibiting at a concentration of 12.5 µg/mL and 100 µg/mL, respectively. Complex **2** had similar inhibitory activity to complex **3**, however no activity was observed for *A. baumannii* or *E. coli* I469 EBL (Table 3). Bactericidal activity for complex **2** was greatly reduced compared to complex **3** with an MBC observed for only *S. typhi* and *S. aureus*.

**Table 3:** The minimum inhibitory concentrations observed for all organisms tested.

	Minimum Inhibitory Concentration (MIC) µg/mL			
Organism	Complex 1	Complex 2	Complex 3	Complex 4
***Acinetobacter baumannii* NCTC 12156**	>100 (>216.9 µM)	>100 (>210.5 µM)	50 (94.5 µM)	>100 (>191.2 µM)
***Escherichia coli* ATCC 11775**	>100 (>216.9 µM)	100 (210.5 µM)	50 (94.5 µM)	>100 (>191.2 µM)
***Escherichia coli* I469 ESBL**	>100 (>216.9 µM)	>100 (>210.5 µM)	50 (94.5 µM)	>100 (>191.2 µM)
***Escherichia coli* J53 2138E**	>100 (>216.9 µM)	50 (105.2 µM)	25 (47.25 µM)	>100 (>191.2 µM)
***Escherichia coli* J53 2140E**	>100 (>216.9 µM)	100 (210.5 µM)	25 (47.25 µM)	>100 (>191.2 µM)
***Klebsiella pneumoniae* H467 KPC**	>100 (>216.9 µM)	>100 (>210.5 µM)	>100 (<189 µM)	>100 (>191.2 µM)
***Mycobacterium abscessus* NCTC 13031**	50 (108.4 µM)	25 (52.6 µM)	12.5 (23.6 µM)	>100 (>191.2 µM)
***Mycobacterium tuberculosis* H37Rv**	50 (108.4 µM)	100 (210.5 µM)	100 (189 µM)	50 (95.6 µM)
***Proteus mirabilis* NCTC 8309**	>100 (>216.9 µM)	>100 (>210.5 µM)	>100 (<189 µM)	>100 (>191.2 µM)
***Pseudomonas aueruginosa* ATCC 10145**	>100 (>216.9 µM)	>100 (>210.5 µM)	>100 (<189 µM)	>100 (>191.2 µM)
***Salmonella enterica* serovar Typhi**	50 (108.4 µM)	25 (52.6 µM)	6.25 (11.8 µM)	>100 (>191.2 µM)
***Staphylococcus aureus* ATCC 29213**	6.25 (13.5 µM)	12.5 (26.3 µM)	1.56 (2.94 µM)	12.5 (23.9 µM)

**Table 4:** The minimum bactericidal concentrations observed for all organisms tested.

	Minimum Bactericidal Concentration (MBC) µg/mL			
Organism	Complex 1	Complex 2	Complex 3	Complex 4
***Acinetobacter baumannii* NCTC 12156**	>100 (>216.9 µM)	>100 (>210.5 µM)	50 (94.5 µM)	>100 (>191.2 µM)
***Escherichia coli* ATCC 11775**	>100 (>216.9 µM)	>100 (>210.5 µM)	50 (94.5 µM)	>100 (>191.2 µM)
***Escherichia coli* I469 ESBL**	>100 (>216.9 µM)	>100 (>210.5 µM)	50 (94.5 µM)	>100 (>191.2 µM)
***Escherichia coli* J53 2138E**	>100 (>216.9 µM)	>100 (>210.5 µM)	25 (47.25 µM)	>100 (>191.2 µM)
***Escherichia coli* J53 2140E**	>100 (>216.9 µM)	>100 (>210.5 µM)	25 (47.25 µM)	>100 (>191.2 µM)
***Klebsiella pneumoniae* H467 KPC**	>100 (>216.9 µM)	>100 (>210.5 µM)	>100 (<189 µM)	>100 (>191.2 µM)
***Mycobacterium abscessus* NCTC 13031**	>100 (>216.9 µM)	>100 (>210.5 µM)	>100 (<189 µM)	>100 (>191.2 µM)
***Mycobacterium tuberculosis* H37Rv**	50 (108.4 µM)	>100 (>210.5 µM)	>100 (<189 µM)	50 (95.6 µM)
***Proteus mirabilis* NCTC 8309**	>100 (>216.9 µM)	>100 (>210.5 µM)	>100 (<189 µM)	>100 (>191.2 µM)
***Pseudomonas aueruginosa* ATCC 10145**	>100 (>216.9 µM)	>100 (>210.5 µM)	>100 (<189 µM)	>100 (>191.2 µM)
***Salmonella enterica* serovar Typhi**	50 (108.4 µM)	25 (52.6 µM)	6.25 (11.8 µM)	>100 (>191.2 µM)
***Staphylococcus aureus* ATCC 29213**	12.5 (27.11 µM)	12.5 (26.3 µM)	3.125 (5.9 µM)	50 (95.6 µM)

Complex **1** was less effective, only inhibiting *M. abscessus, M. tuberculosis, S. typhi* and *S. aureus*. No bactericidal activity was observed for *M. abscessus* but bactericidal activity was observed for *M. tuberculosis, S. ty*ph*i* and *S. aureus*. Arguably complex **4** was the least effective, with an MIC and MBC observed for only *S. aureus* and *M. tuberculosis* (Fig. 6). However, *M. tuberculosis* is a major human pathogen that is often highly drug resistant. Therefore, complex **4** is still of great potential importance and could be effective against other pathogens not yet tested.

**Fig. 5:**
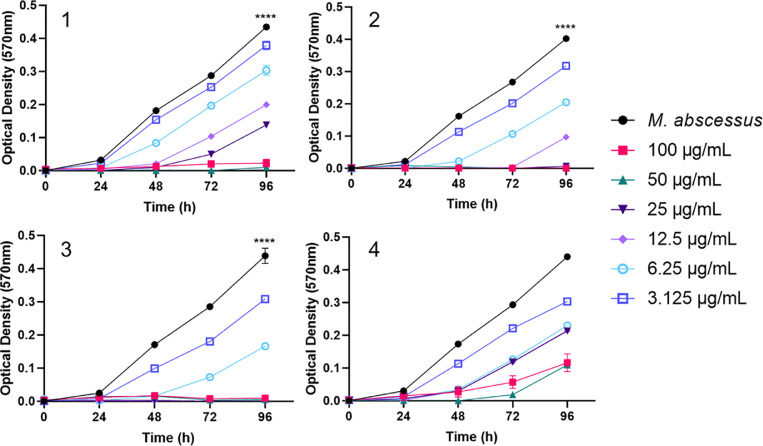
Spectrophotometric growth curves of *Mycobacterium abscessus* with complexes **1** – **4** showing bacteriostatic activity. (1) Activity of complex **1** against *M. abscessus* identifying the minimum inhibitory concentration (MIC) as 50 µg/mL (n=3). A one-way ANOVA was used on endpoint data and identified a significant difference between all concentrations and the control (p value <0.0001), apart from 3.125 µg/mL (p value 0.0064). (2) Complex **2** exhibited increased activity, with an MIC of 25 µg/mL (n=3). All concentrations were significantly different to the growth of *M. abscessus* only (p value <0.0001). (3) Complex **3** was the most effective of the complexes tested, inhibiting *M. abscessus* at 12.5 µg/mL (n=3). All concentrations impacted the growth of *M. abscessus* significantly (p value <0.0001). (4) Complex **4** had no MIC observed and did not significantly impact the growth of *M. abscessus* (n=3) (p value 0.2960).

Growth curve data was obtained for *M. abscessus* (Fig. 5) and analysed with an ANOVA. A significant difference between the different concentrations and controls was observed for complexes **1, 2** and **3** with p values of <0.0001 for all three complexes. No significant difference between concentrations and control were observed for complex **4**. A multiple comparison was conducted for complexes **1, 2** and **3**, which identified a significant difference between all concentrations and control (p value <0.0001) for all, apart from complex **1** at 3.125 µg/mL and the *M. abscessus* only control (p value 0.0064). This identifies that complexes **1, 2** and **3** are bacteriostatic against *M. abscessus* and have a significant impact on the growth.

**Fig. 6:**
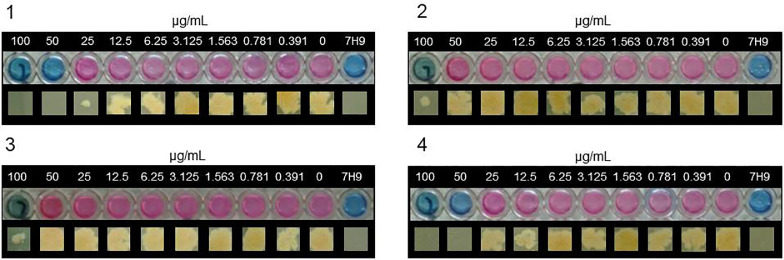
Minimum Inhibitory Concentrations (MIC) (upper) and Minimum Bactericidal Concentrations (MBC) (lower) of *Mycobacterium tuberculosis* with complexes **1** – **4**. (MBC images contrast adjusted to emphasise growth *vs* no growth). (1) Complex **1** shows an MIC with resazurin of 50 µg/mL and an MBC on solid agar of 50 µg/mL (n=3). (2) Complex **2** shows an MIC with resazurin of 100 µg/mL and no MBC on solid agar (>100 µg/mL) (n=3). (3) Complex **3** shows an MIC with resazurin of 100 µg/mL and no MBC on solid agar (>100 µg/mL) (n=3).(4) Complex **4** shows an MIC with resazurin of 50 µg/mL and an MBC on solid agar of 50 µg/mL (n=3).

All four of the complexes inhibited a variety of microorganisms tested, with complex **3** being the most effective. The Gram-positive *S. aureus* was the most affected organism with the lowest MIC and MBC for all of the complexes tested, as well as being the only one, other than *M. tuberculosis*, affected by complex **4**. Growth of *M. tuberculosis* was also inhibited by each complex, but at higher concentrations than for *S. aureus*. However, unlike *S. aureus*, complexes **2** and **3** had no bactericidal activity against *M. tuberculosis. M. abscessus* was largely inhibited by three out of the four complexes but had no bactericidal activity. A range of activity was identified for the Gram-negative organisms *E. coli* and *S. typhi* against complexes **1, 2** and **3**, however no activity was observed for *Proteus mirabilis, Pseudomonas aeruginosa* or *Klebsiella pneumonia*.

## Conclusions

In conclusion, four half-sandwich metal complexes containing a bioactive indole moiety were synthesised and characterised. They were progressed to *in vitro* screening to gain a first estimation of their cytotoxicity profile. The complexes only show moderate toxicity against A2780 ovarian cancer, A2780 cisplatin resistant, and PNT2 cell lines. As such, the bactericidal activity of the four compounds was investigated against several drug resistant isolates of Gram negative *Escherichia coli* and *Salmonella enterica* serovar Typhi as well as against isolates of Gram positive bacteria.

All complexes showed growth inhibition and bactericidal activity against a variety of bacteria, including the notable pathogens *M. tuberculosis, M. abscessus* and *E. coli*. Complexes **2** and **3** exhibit the most promising antibacterial activities, having the lowest minimum inhibitory concentrations and exhibiting bactericidal activity. It appears that steric hindrance of the R group on the indole has an influence on the aquation rate, the cytotoxicity, and the antibacterial properties, which will be confirmed/infirmed by future determination of the antibacterial mechanism of action. This series shows significant promise for further hit-to-lead medicinal chemistry, and future work will include further cytotoxicity studies, collection of *in vivo* data and mechanisms of action elucidation (target identification).

## Materials and methods

Hydrated metallic chlorides were purchased from Precious Metals Online. All other chemicals were purchased from Sigma-Aldrich (UK). Non-dried solvents were purchased from Fischer Scientific and used as received. Dichloromethane, tetrahydrofuran and toluene were dried over molecular sieves (3 Å). All compounds were prepared under a purified dinitrogen atmosphere using standard Schlenk and vacuum line techniques, unless otherwise specified. pH* was adjusted using EDT direction non-glass pocket pH meter with an ISFET silicon chip pH sensor. pH* values (pH readings without correction for the effect of deuterium) of NMR samples were adjusted using KOD solutions in D_2_O. All NMR spectra were recorded on a 400 MHz Bruker Spectrospin spectrometer using 5 mm NMR tubes. Data processing was carried out using TOPSPIN 4.0.9 (Bruker U.K. Ltd.). Deuterated solvents were purchased from Goss Scientific Instrument. ^1^H NMR chemical shifts were internally referenced to TMS *via* residual solvent peaks DMSO (*δ* = 2.52 ppm), CHCl_3_ (*δ* = 7.26 ppm), acetone (*δ* = 2.05 ppm), THF (*δ* = 1.72 ppm) or MeOD (*δ* = 3.31 ppm). All UV-Vis spectra were recorded with Agilent 60 Cary UV-Vis spectrophotometer. Ruthenium dimer [(*p*-cym)RuCl_2_]_2_ was synthesised using an Anton Paar microwave synthesis reactor (Monowave 300) and a 20 mL microwave vial equipped with a magnetic stirring bar.

Roswell Park Memorial Institute (RPMI) 1640 medium, foetal bovine serum (FBS), penicillin and streptomycin, phosphate-buffered saline (PBS, pH 7.4), and other tissue culture reagents were purchased from Gibco (Thermo Fisher Scientific, UK). Cell lines were provided by the Institute of Cancer Therapeutics, University of Bradford. Cells were incubated in a ThermoScientific HERAcell 150 incubator and observed under a Nikon ECLIPSE TS100 Microscope.

### Bacterial culture

All chemicals and reagents were obtained from Sigma-Aldrich or Fisher Scientific, unless otherwise stated. The media used for *Mycobacterium abscessus* NCTC 13031 and *Mycobacterium tuberculosis* H37Rv was Middlebrook 7H9 broth with the addition of glycerol (0.4%), Albumin-Dextrose-Catalase (ADC) supplement (10%) and Tween80 (0.05%) and Middlebrook 7H11 agar with glycerol (0.5%) and Oleic acid-Albumin-Dextrose-Catalase (OADC) supplement (10%). The media used for all other organisms (*Escherichia coli* ATCC 11775, *E. coli* I469 ESBL, *E. coli* J53 2138E, *E.* coli J53 2140E, *Klebsiella pneumonia* H467 KPC, *Proteus mirabilis* NCTC 8309, *Pseudomonas aeruginosa* ATCC 10145, *Salmonella enterica* serovar *Typhi, Staphylococcus aureus* ATCC 29213) was nutrient broth and nutrient agar. Prior to testing, all organisms were grown in broth to log phase at 37°C with orbital shaking at 180 rpm. For *M. abscessus* this was 72 h, for *M. tuberculosis* this was 168 h and all other organisms 24 h.

### Synthesis and characterisation

**[Methyl indole-2-carboxylate] (L2).** 1-*H*-indole-2-carboxylic acid (3.0 g, 18.6 mmol) was added to a round-bottom flask (100 mL) and dissolved in methanol (30 mL). Concentrated sulphuric acid (1.5 mL, 28.0 mmol) was added to the mixture and subsequently heated to reflux for 2 h. Then, the solvent was removed under reduced pressure, and the crude was dissolved in ethyl acetate (6 mL), and subsequently extracted with a saturated solution of NaHCO_3_ (3×30 mL). The organic phases were combined and dried over MgSO_4_ and filtered. The filtrate was evaporated under vacuum to afford a light-brown powder (4.3 g, 93%). The synthesis and characterisation are in accordance with the literature. (Mistry et al., 2015)

**Figure:**
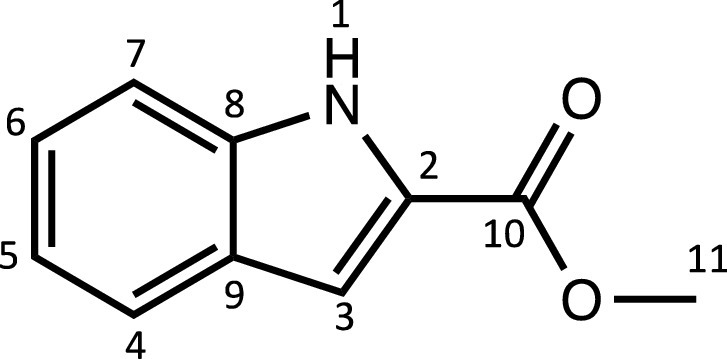

^1^H NMR (DMSO-d_6_, 400 MHz): δ = 11.91 (1H, s, H1), 7.65 (1H, d, ^3^*J*_H-H_ = 8.0 Hz, H4), 7.45 (1H, d, ^3^*J*_H-H_ = 8.3 Hz, H7), 7.26 (1H, dd, ^3^*J*_H-H_ = 6.8 and 8.0 Hz, H6), 7.15 (1H, m, H3), 7.07 (1H, dd, ^3^*J*_H-H_ = 6.8 and 8.0 Hz, H5), 3.87 ppm (3H, s, H11).

^13^C{^1^H} NMR (DMSO-d_6_, 101 MHz): δ = 161.8 (C10), 137.4 (C2), 127.0 (C8 or C9), 126.7 (C8 or C9), 124.7 (C6), 122.1 (C4), 120.2 (C5), 112.6 (C7), 107.8 (C3), 51.8 ppm (C11).

HRMS (ESI+): m/z calc. for C_10_H_10_NO_2_ [M + H]^+^ 176.0667; found 176.0702.

**[Ethyl indole-2-carboxylate] (L3).** The procedure used to prepare **L2** was followed with 30 mL of ethanol and 18.6 mmol of 1-*H*-indole-2-carboxylic acid. The pure compound **L3** was obtained as a white powder (4.1 g, 91%). The synthesis and characterisation are in accordance with the literature. (Kuuloja et al., 2011)

**Figure:**
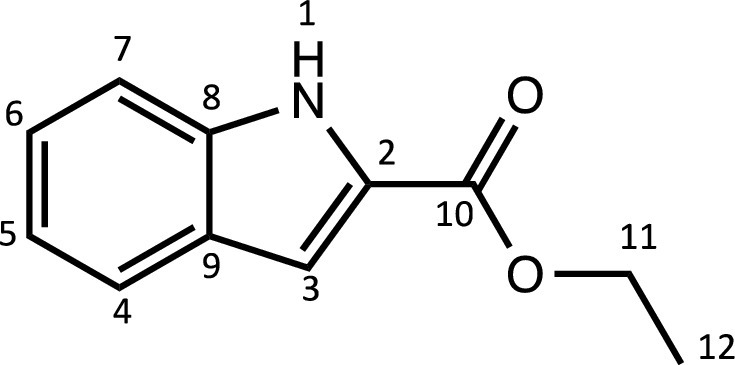

^1^H NMR (DMSO-d_6_, 400 MHz): δ = 11.86 (1H, s, H1), 7.65 (1H, d, ^3^*J*_H-H_ = 8.0 Hz, H4), 7.45 (1H, d, ^3^*J*_H-H_ = 8.4 Hz, H7), 7.25 (1H, dd, ^3^*J*_H-H_ = 7.2 and 8.4 Hz, H6), 7.14 (1H, m, H3), 7.07 (1H, dd, ^3^*J*_H-H_ = 7.2 and 8.0 Hz, H5), 4.34 (2H, q, ^3^*J*_H-H_ = 7.1 Hz, H11), 1.34 ppm (3H, t, ^3^*J*_H-H_ = 7.0 Hz, H12).

^13^C{^1^H} NMR (DMSO-d_6_, 101 MHz): δ = 161.3 (C10), 137.4 (C2), 127.3 (C8 or C9), 126.7 (C8 or C9), 124.6 (C6), 122.1 (C4), 120.2 (C5), 112.6 (C7), 107.7 (C3), 60.4 (C11), 14.3 ppm (C12).

HRMS (ESI+): m/z calc. for C_10_H_10_NO_2_ [M + H]^+^ 190.0823; found 190.08564.

**[Cyclohexyl indole-2-carboxylate] (L4).** The procedure used to prepare **L3** was followed with 10 mL of cyclohexanol and 18.6 mmol of 1-*H*-indole-2-carboxylic acid. The pure compound **L4** was obtained as a light-brown powder (4 g, 91%).

**Figure:**
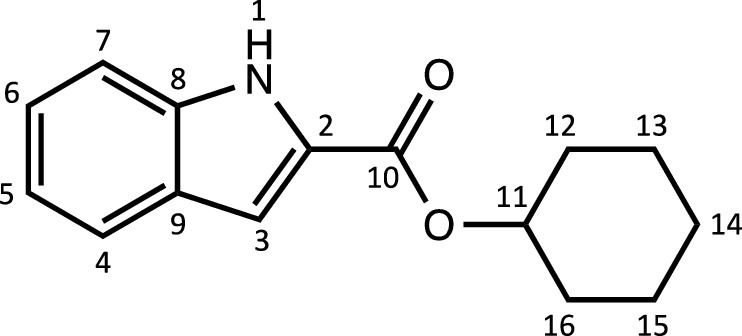

^1^H NMR (DMSO-d_6_, 400 MHz): δ = 11.72 (1H, s, H1), 7.55 (1H, d, ^3^*J*_H-H_ = 8.0 Hz, H4), 7.37 (1H, d, ^3^*J*_H-H_ = 8.4 Hz, H7), 7.16 (1H, dd, ^3^*J*_H-H_ = 7.2 and 7.8 Hz, H6), 7.05 (1H, m, H3), 6.98 (1H, dd, ^3^*J*_H-H_ = 7.0 and 7.6 Hz, H5), 4.86 (1H, m, H11), 1.86 – 1.15 ppm (10H, br, H_cyclohexane_).

^13^C{^1^H} NMR (DMSO-d_6_, 101 MHz): δ = 160.8 (C10), 137.4 (C2), 127.7 (C8 or C9), 126.8 (C8 or C9), 124.6 (C6), 122.0 (C4), 120.1 (C5), 112.6 (C7), 107.7 (C3), 72.3 (C11), 31.2 (C12, C16), 25.0 (C14), 23.1 ppm (C13, C15).

HRMS (ESI+): m/z calc. for C_15_H_18_NO_2_ [M + H]^+^ 244.1293; found 245.13568.

**[Phenyl indole-2-carboxylate] (L5).** 1-*H*-indole-2-carboxylic acid (5.0 g, 31.0 mmol) was added to a round-bottom flask (100 mL). Phenol (2.92 g, 31.0 mmol), *N,N*-dimethyl-4-aminopyridine (DMAP, 1 g, 8.19 mmol), 1-(3-dimethylaminopropyl)-3-ethylcarbodiimide hydrochloride (EDC, 8.0 g, 51.53 mmol) and dry dichloromethane (60 mL) were added to the mixture and subsequently heated to reflux for 24 h. Then, the crude solution was quenched with a saturated aqueous solution of NaHCO_3_ and extracted with H_2_O (3×30 mL). The organic phases were combined and dried over MgSO_4_ and filtered. The filtrate was evaporated under vacuum to afford a white powder (2.8 g, 37%). The synthesis and characterisation are in accordance with the literature. (Guo et al., 2016)

**Figure:**
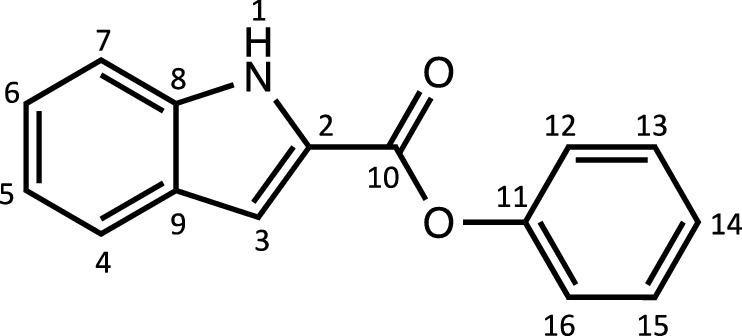

^1^H NMR (DMSO-d_6_, 400 MHz): δ = 12.15 (1H, s, H1), 7.72 (1H, d, ^3^*J*_H-H_ = 8.0 Hz, H4), 7.49 (3H, dd, ^3^*J*_H-H_ = 8.8 and 9.2 Hz, H7, H13 and H15_._)_,_ 7.41 (1H, m, H3), 7.32 (4H, m, H6, H12, H14 and H16), 7.13 ppm (1H, dd, ^3^*J*_H-H_ = 7.4 and 7.6Hz, H5).

^13^C{^1^H} NMR (DMSO-d_6_, 101 MHz): δ = 159.8 (C10), 150.3 (C11), 137.8 (C2), 129.6 (C13 and C15), 126.7 (C8 or C9), 126.2 (C8 or C9), 126.0 (C6 or C14), 125.2 (C6 or C14), 122.3 (C4), 122.0 (C12 and C16_._), 120.5 (C5), 112.7 (C7), 109.5 ppm (C3).

HRMS (ESI+): m/z calc. for C_15_H_12_NO_2_ [M + H]^+^ 238.0823; found 238.08532.

**[Methyl indole-2-thionoester] (L6). L2** (250 mg 1.43 mmol), and Lawesson's reagent (693 mg, 1.7 mmol) were added to a round-bottom flask and dissolved in toluene (35 mL). The mixture was refluxed for 6 days. The solution was cooled down and toluene was removed under reduced pressure. The crude was dissolved in ethyl acetate and extracted first with an aqueous saturated solution of NaHCO_3_, and then with brine, and dried over MgSO_4_ and filtered. The combined organic phases were brought to dryness, leaving a bright yellow oil, which was purified by column chromatography (hexane/ethyl acetate, 80:20 (v/v)). The solvent was removed under vacuum to give a bright yellow solid (203 mg, 74%).

**Figure:**
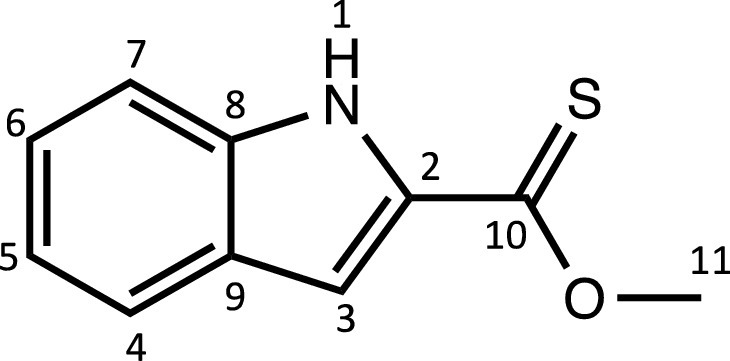

^1^H NMR (DMSO-d_6_, 400 MHz): δ = 11.75 (1H, s, H1), 7.67 (1H, d, ^3^*J*_H-H_ = 8.4 Hz, H4), 7.48 (1H, d, ^3^*J*_H-H_ = 8.4 Hz, H7), 7.28 (2H, m, H3 and H6), 7.07 (1H, dd, ^3^*J*_H-H_ = 7.6 and 8.0 Hz, H5), 4.24 ppm (3H, s, H11).

^13^C{^1^H} NMR (DMSO-d_6_, 101 MHz): δ = 201.7 (C10), 138.4 (C2), 136.4 (C8 or C9), 126.9 (C8 or C9), 125.6 (C6), 122.6 (C4), 120.6 (C5), 112.9 (C7), 107.2 (C3), 58.4 ppm (C11).

HRMS (ESI+): m/z calc. for C_10_H_10_NOS [M + H]^+^ 192.0483; found 192.0475.

**[Ethyl indole-2-thionoester] (L7).** The procedure used to prepare **L6** was followed with 250 mg (1.3 mmol) of **L3** and Lawesson's reagent (623 mg, 1.57 mmol) and refluxed for 6 days. The pure compound **L7** was obtained as a bright yellow solid (220 mg, 81%).

**Figure:**
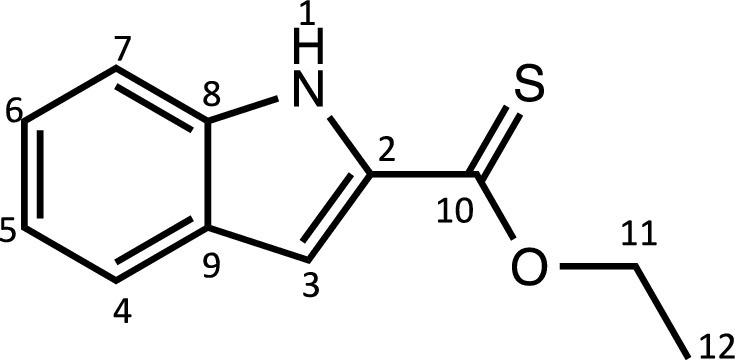

^1^H NMR (DMSO-d_6_, 400 MHz): δ = 11.69 (1H, s, H1), 7.67 (1H, d, ^3^*J*_H-H_ = 8.4 Hz, H4), 7.49 (1H, d, ^3^*J*_H-H_ = 8.4 Hz, H7), 7.28 (2H, m, H3 and H6), 7.07 (1H, dd, ^3^*J*_H-H_ = 7.6 and 8.0Hz, H5), 4.70 (2H, q, ^3^*J*_H-H_ = 7.1 Hz, H11), 1.47 ppm (3H, t, ^3^*J*_H-H_ = 7.0 Hz, H12).

^13^C{^1^H} NMR (DMSO-d_6_, 101 MHz): δ = 201.0 (C10), 138.3 (C2), 136.6 (C8 or C9), 126.9 (C8 or C9), 125.6 (C6), 122.6 (C4), 120.6 (C5), 112.9 (C7), 107.1 (C3), 67.4 (C11), 13.7 ppm (C12).

HRMS (ESI+): m/z calc. for C_11_H_12_NOS [M + H]^+^ 206.0595; found 206.0632.

**[Cyclohexyl indole-2-thionoester] (L8).** The procedure used to prepare **L6** was followed with 250 mg (1.0 mmol) of **L4** and Lawesson's Reagent (499 mg, 1.23 mmol) and refluxed for 4 days. The pure compound **L8** was obtained as a bright yellow solid (190 mg, 71%).

**Figure:**
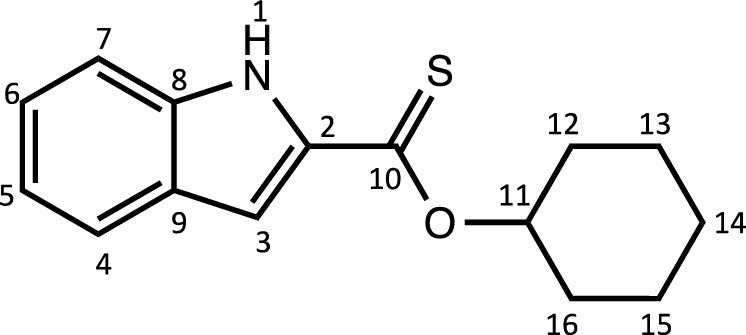

^1^H NMR (DMSO-d_6_, 400 MHz): δ = 11.62 (1H, s, H1), 7.67 (1H, d, ^3^*J*_H-H_ = 8.0 Hz, H4), 7.49 (1H, d, ^3^*J*_H-H_ = 8.0 Hz, H7), 7.28 (1H, dd, ^3^*J*_H-H_ = 7.8 and 8.4 Hz, H6), 7.25 (1H, m, H3), 7.07 (1H,dd, ^3^*J*_H-H_ = 7.6 and 8.0 Hz, H5), 5.58 (1H, m, H11), 2.05 – 1.33 ppm (10H, br, H_cyclohexane_).

^13^C{^1^H} NMR (DMSO-d_6_, 101 MHz): δ = 200.0 (C10), 138.3 (C2), 136.9 (C8 or C9), 126.9 (C8 or C9), 125.5 (C6), 122.6 (C4), 120.6 (C5), 112.9 (C7), 107.0 (C3), 78.7 (C11), 30.4 (C12, C16), 24.9 (C14), 23.0 ppm (C13, C15).

HRMS (ESI+): m/z calc. for C_15_H_18_NOS [M + H]^+^ 260.1064; found 260.1099.

**[Phenyl indole-2-thionoester] (L9):** The procedure used to prepare **L6** was followed with 250 mg (1.1 mmol) of **L5** and Lawesson's Reagent (511 mg, 1.26 mmol) and refluxed for 8 days. The pure compound **L9** was obtained as a bright yellow solid (100 mg, 37%).

**Figure:**
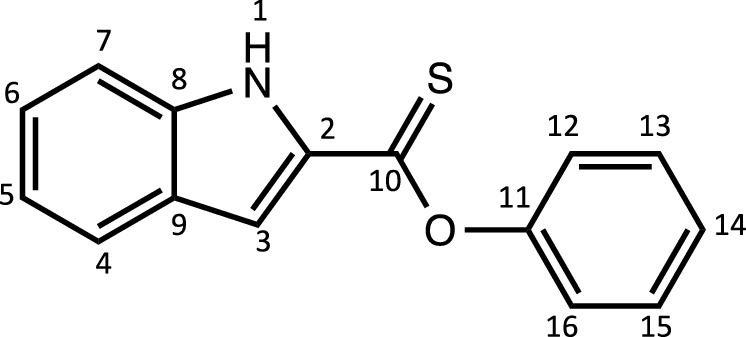

^1^H NMR (DMSO-d_6_, 400 MHz): δ = 11.96 (1H, s, H1), 7.72 (1H, d, ^3^*J*_H-H_ = 8.0 Hz, H4), 7.55 – 7.48 (4H, m, H3, H7, H13 and H15)_,_ 7.37 – 7.32 (2H, m, H6 and H14), 7.24 (2H, d, ^3^*J*_H-H_ = 7.6 Hz, H12 and H16), 7.11 ppm (1H, dd, ^3^*J*_H-H_ = 7.6 and 8.0 Hz, H5).

^13^C{^1^H} NMR (DMSO-d_6_, 101 MHz): δ = 200.0 (C10), 153.7 (C11), 138.9 (C2), 136.4 (C9 or C9), 129.7 (C13 and C15), 127.0 (C8 or C9, 126.5 (C6 or C14), 126.3 (C6 or C14), 122.9 (C4), 122.4 (C12 and C16), 120.9 (C5), 113.0 (C7), 108.9 ppm (C3).

HRMS (ESI+): m/z calc. for C_15_H_12_NOS [M + H]^+^ 254.0595; found 254.0631.

**[(*p*-cym)RuCl(methyl indole-2-thionoester)] (1).**

Ruthenium dimer [(*p*-cym)RuCl_2_]_2_ (70 mg, 0.11 mmol) and **L6** (46 mg, 0.24 mmol) were placed in a 50 mL round-bottom flask and dissolved in 15 mL of dry dichloromethane. Once dissolved, 62 μL of dry triethylamine (0.46 mmol) were added to the mixture. The bright orange mixture was stirred under nitrogen overnight at 25 ℃, until a brown solution was obtained. The crude was extracted with an aqueous solution of 0.1 M HCl (3×10 mL) and the combined organic phases were dried over MgSO_4_ and filtered. The product was purified by column chromatography (ethyl acetate/hexane 60:40 (v/v)) and recrystallised in dichloromethane/hexane to obtain a bright red solid (30 mg, 58%).

**Figure:**
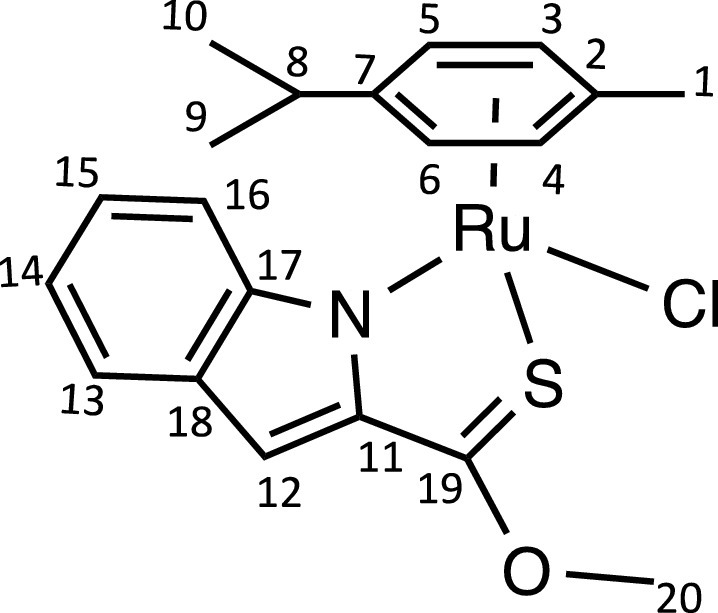

^1^H NMR (CDCl_3_, 400 MHz): δ = 7.59 (1H, d, ^3^*J*_H-H_ = 8.4 Hz, H13), 7.56 (1H, d, ^3^*J*_H-H_ = 8.4 Hz, H16), 7.22 (1H, dd, ^3^*J*_H-H_ = 6.8 and 8.0, H15), 7.07 (1H, s, H12), 6.96 (1H, dd, ^3^*J*_H-H_ = 6.8 and 8.0 Hz, H14), 5.83 (2H, q, ^3^*J*_H-H_ = 6.2 Hz, H3 and H4), 5.72 (1H, d, ^3^*J*_H-H_ = 5.8 Hz, H6), 5.58 (1H, d, ^3^*J*_H-H_ = 6.0 Hz, H5), 4.37 (3H, s, H20), 2.53 (1H, sept, ^3^*J*_H-H_ = 6.9 Hz, H8), 2.25 (3H, s, H1), 1.07 (3H, d, ^3^*J*_H-H_ = 6.9 Hz, H10), 0.92 ppm (3H, d, ^3^*J*_H-H_ = 6.9 Hz, H9).

^13^C{^1^H} NMR (CDCl_3_, 101 MHz): δ = 206.6 (C19), 152.0 (C11), 144.4 (C17 or C18), 130.9 (C17 or C18), 125.5 (C15), 124.5 (C13), 120.0 (C14), 116.8 (C16), 108.2 (C12), 103.0 (C7), 102.3 (C2), 85.9 (C6), 83.7 (C3 or C4), 83.2 (C3 or C4), 82.0 (C5), 60.1 (C20), 31.1 (C8), 23.0 (C10), 21.8 (C9), 19.0 ppm (C1).

HRMS (ESI+): m/z calc. for C_20_H_22_NORuS [M - Cl]^+^ 426.0466; found 426.0452.

**[(*p*-cym)RuCl(ethyl indole-2-thionoester)] (2).**

Complex **2** was synthesised following the procedure of complex **1** with **L7** (50 mg, 0.24 mmol). The pure compound **2** was obtained as a bright red solid (30 mg, 56%).

**Figure:**
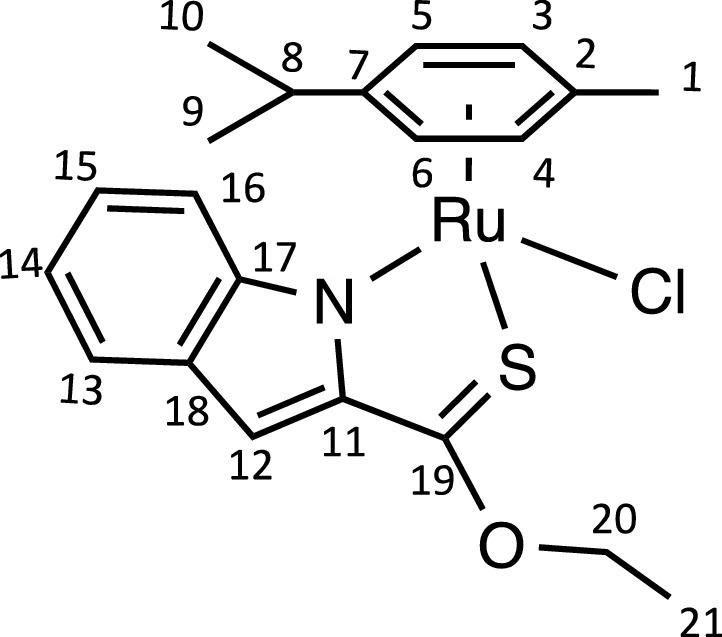

^1^H NMR (CDCl_3_, 400 MHz): δ = 7.58 (2H, m, H13, H16), 7.21 (1H, dd, ^3^*J*_H-H_ = 6.8 and 8.0 Hz, H15), 7.08 (1H, s, H12), 6.96 (1H, dd, ^3^*J*_H-H_ = 6.8 and 7.6 Hz, H14), 5.82 (2H, q, ^3^*J*_H-H_ = 6.0 Hz, H3, H4), 5.71 (1H, d, ^3^*J*_H-H_ = 5.9 Hz, H6), 5.56 (1H, d, ^3^*J*_H-H_ = 5.9 Hz, H5), 4.74 (2H, q, ^3^*J*_H-H_ = 7.1 Hz, H20), 2.53 (1H, sept, ^3^*J*_H-H_ = 6.9 Hz, H8), 2.25 (3H, s, H1), 1.55 (3H, t, ^3^*J*_H-H_ = 7.0 Hz, H21), 1.07 (3H, d, ^3^*J*_H-H_ = 7.2 Hz, H10), 0.92 ppm (3H, d, ^3^*J*_H-H_ = 6.9 Hz, H9).

^13^C{^1^H} NMR (CDCl_3_, 101 MHz): δ = 205.8 (C19), 151.9 (C11), 144.5 (C17 or C18), 130.9 (C17 or C18), 125.3 (C15), 124.5 (C13), 119.9 (C14), 116.8 (C16), 108.0 (C12), 103.0 (C7), 102.1 (C2), 85.9 (C6), 83.7 (C3 or C4), 83.2 (C3 or C4), 82.0 (C5), 69.8 (C20), 31.1 (C8), 23.0 (C10), 21.8 (C9), 19.1 (C1), 14.4 ppm (C21).

HRMS (ESI+): m/z calc. for C_21_H_24_NORuS [M - Cl]^+^ 440.0622; found 440.0614.

**[(*p*-cym)RuCl(cyclohexyl indole-2-thionoester)] (3).**

Complex **3** was synthesised following the procedure of complex **1** with **L8** (62 mg, 0.24 mmol). The pure compound **3** was obtained as a bright red solid (39 mg, 65%).

**Figure:**
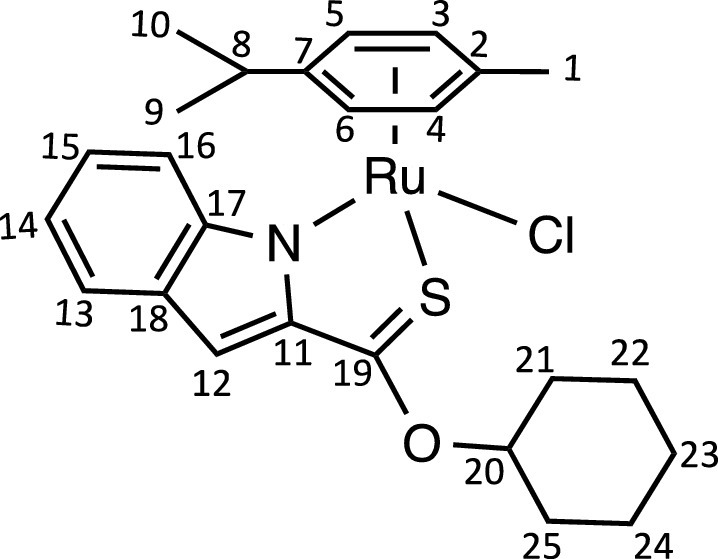

^1^H NMR (CDCl_3_, 400 MHz): δ = 7.58 (2H, m, H13, H16), 7.21 (1H, dd, ^3^*J*_H-H_ = 6.8 and 8.0 Hz, H15), 7.07 (1H, s, H12), 6.95 (1H, dd, ^3^*J*_H-H_ = 6.8 and 7.6 Hz, H14), 5.80 (2H, m, H3, H4), 5.70 (1H, d, ^3^*J*_H-H_ = 6.0 Hz, H6), 5.55 (1H, d, ^3^*J*_H-H_ = 5.9 Hz, H5), 5.37 (1H, m, H20), 2.52 (1H, sept, ^3^*J*_H-H_ = 6.9 Hz, H8), 2.25 (3H, s, H1), 2.20 – 1.24 (10H, br, H_cyclohexane_), 1.07 (3H, d, ^3^*J*_H-H_ = 7.0 Hz, H10), 0.93 ppm (3H, d, ^3^*J*_H-H_ = 7.0 Hz, H9).

^13^C{^1^H} NMR (CDCl_3_, 101 MHz): δ = 204.8 (C19), 151.8 (C11), 145.0 (C17 or C18), 130.8 (C17 or C18), 125.2 (C15), 124.4 (C13), 119.8 (C14), 116.8 (C16), 107.9 (C12), 103.0 (C7), 101.9 (C2), 85.9 (C6), 83.8 (C3 or C4), 83.3 (C3 or C4), 82.9 (C20), 82.0 (C5), 31.5 (CH_2cyclohex._), 31.1 (C8), 25.4 (CH_2cyclohex._), 23.7 (2CH_2cyclohex._), 23.6 (CH_2cyclohex._) 22.9 (C10), 21.8 (C9), 19.0 ppm (C1).

HRMS (ESI+): m/z calc. for C_25_H_30_NORuS [M - Cl]^+^ 494.1092; found 494.1088.

**[(*p*-cym)RuCl(phenyl indole-2-thionoester)] (4).**

Complex **4** was synthesised following the procedure of complex **1** with **L9** (61 mg, 0.24 mmol). The pure compound **4** was obtained as a bright red solid (31 mg, 51%).

**Figure:**
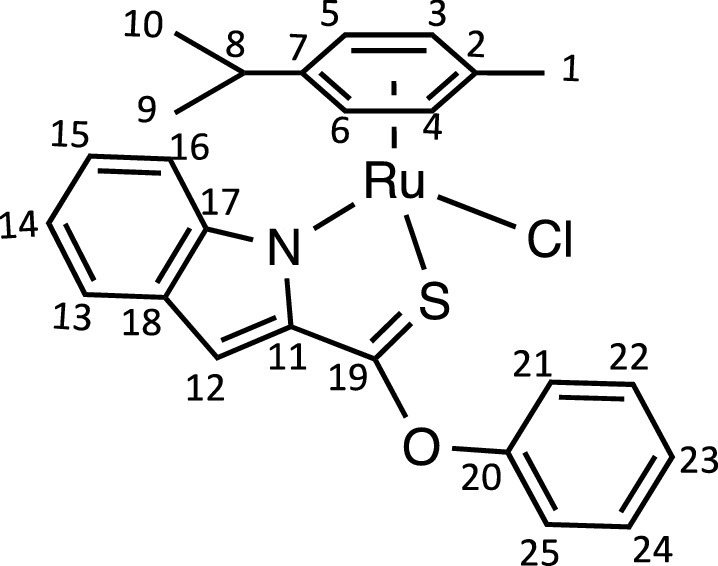

^1^H NMR (CDCl_3_, 400 MHz): δ= 7.63 (1H, d, ^3^*J*_H-H_ = 8.2 Hz, H13), 7.57 (1H, d, ^3^*J*_H-H_ = 8.7 Hz, H16), 7.48 (2H, m, H22 and H24), 7.37 (1H, t, ^3^*J*_H-H_ = 7.4 Hz, H23), 7.29 – 7.21 (4H, m, H12, H15, H21 and H25), 6.99 (1H, dd, ^3^*J*_H-H_ = 6.4 and 8.0 Hz, H14), 5.83 (1H, d, ^3^*J*_H-H_ = 6.0 Hz, H4), 5.80 (1H, d, ^3^*J*_H-H_ = 6.0 Hz, H3), 5.64 (1H, d, ^3^*J*_H-H_ = 5.9 Hz, H6), 5.49 (1H, d, ^3^*J*_H-H_ = 5.9 Hz, H5), 2.49 (1H, sept, ^3^*J*_H-H_ = 6.9 Hz, H8), 2.24 (3H, s, H1), 1.06 (3H, d, ^4^*J*_H-H_ = 7.0 Hz, H10), 0.94 ppm (3H, d, ^4^*J*_H-H_ = 7.0 Hz, H9).

^13^C{^1^H} NMR (CDCl_3_, 101 MHz): δ = 204.9 (C19), 154.8 (C20), 153.0 (C11), 144.8 (C17 or C18), 131.1 (C17 or C18), 130.0 (C22 and C24), 127.2 (C23), 126.2 (C15), 124.9 (C13), 122.0 (C21 and C25), 120.4 (C14), 116.9 (C16), 109.5 (C12), 103.3 (C7), 102.1 (C2), 85.9 (C6), 83.9 (C4), 83.5 (C3), 81.8 (C5), 31.1 (C8), 22.8 (C10), 21.9 (C9), 19.0 ppm (C1).

HRMS (ESI+): m/z calc. for C_25_H_24_NORuS [M - Cl]^+^ 488.0622; found 488.0617.

### Solution chemistry

Stability determination: Complexes **1** – **4** were dissolved in MeOD (2.2 mM) and diluted to a final concentration of 1.1 mM with either MeOD or D_2_O. ^1^H NMR spectra were recorded at t ≤ 10 min, 12 h and 24 h. p*K*a determination: Complexes **1** – **4** were dissolved in acetonitrile/H_2_O 6:94 (v/v) and UV-Vis spectra of the samples were recorded increasing the pH from 7 to 12. p*K*a value of each complex was determined using Origin 2019 by plotting the absorbance against the pH.

Hydrolysis rate: Complexes **1** – **4** were dissolved in DMSO/RPMI 1:1 (v/v) (3×10^−5^ M) at room temperature and UV-Vis spectra of the samples were recorded at different times between t ≤ 10 min and 24 h. The dissociation constant values were calculated using Origin 2019 by plotting the absorbance against the time at fixed wavelength.

### Chemosensitivity Assays

*In vitro* chemosensitivity tests were performed against A2780, A2780cisR and PNT2 cells. Cancer cell lines were routinely maintained as monolayer cultures in RPMI medium supplemented with 10% foetal calf serum, penicillin (100 I.U./mL), streptomycin (100 μg/mL), sodium pyruvate (1 mM), and L-glutamine (2 mM). For chemosensitivity studies, cells were incubated in 96-well plates at a concentration of 7.5 × 10^3^ cells per well and the plates were incubated for 24 h at 37 °C and a 5% CO_2_ humidified atmosphere prior to drug exposure.

Complexes were dissolved in dimethylsulfoxide (DMSO) to provide stock solutions which were further diluted with media to provide a range of final concentrations. Drug-media solutions were added to cells (the final concentration of DMSO was less than 1% (v/v) in all cases) and incubated for 24 h at 37 °C and 5% CO_2_ humidified atmosphere. The drug-media was removed from the wells and the cells were washed with PBS (100 μL, twice), and 100 μL of complete fresh media were added to each well. The plates were further incubated for 48 h at 37 °C in a 5% CO_2_ humidified atmosphere to allow for a period of recovery. 3-(4,5-dimethylthiazol-2-yl)-2,5-diphenyltetrazolium bromide (MTT) (20 μL, 2.5 mg/mL) was added to each well and incubated for 2 h at 37 °C and 5% CO_2_ humidified atmosphere. All solutions were then removed and 100 μL of DMSO was added to each well in order to dissolve the purple formazan crystals. A Thermo Scientific Multiskan EX microplate photometer was used to measure the absorbance in each well at 570 nm. Cell survival was determined as the absorbance of treated cells divided by the absorbance of controls and expressed as a percentage. The IC_50_ values were determined from plots of % survival against drug concentration. Each experiment was repeated in triplicates of triplicates and a mean value was obtained and stated as IC_50_ (μM) ± SD. Cisplatin was also used as a positive control.

### Bacterial susceptibility testing

A broth microdilution assay was used to determine antimicrobial activity of the compounds. Briefly, complexes **1-4** were made up to a stock solution of 10 mg/mL in DMSO and serially diluted in 96 well plates to final concentrations of 100 µg/mL, 50 µg/mL, 25 µg/mL, 12.5 µg/mL, 6.25 µg/mL and 3.125 µg/mL with 1% DMSO. All bacterial suspensions (except *M. tuberculosis*) were adjusted to an optical density (OD_600_) of 0.1 prior to inoculation. An initial spectrophotometric read was taken using a Biotek EL808 plate reader at 570 nm. The plates were then incubated at 37 °C and read every 24 h for the length of the assay (*M. abscessus* = 96 h, all other organisms = 24 h). Once the spectrophotometric assays were complete, 5 µL of each well was plated out onto appropriate agar and incubated at 37 °C for 24-48 h to check for bactericidal activity. All data was processed through GraphPad Prism 8.

*M. tuberculosis* was assessed by addition of OD_600_ adjusted culture (OD_600_=0.001) to experimental wells containing serially diluted complexes **1-4** (as previously described). Plates were incubated for 168 h at 37 °C, before 5 µL of each experimental well was plated onto Middlebrook 7H11 agar with OADC. These agar plates were incubated at 37 °C for 312 h. The drug containing 96-well plates were also additionally incubated for a further 240 h at 37 °C, after which resazurin (0.0025%) was added and plates were incubated at 37 °C for a final 24 h. Minimum inhibitory concentrations were determined by conversion of resazurin to resorufin. Minimum bactericidal concentrations were determined by the lowest concentration in which no growth was visibly seen on the solid agar plates.

## CRediT authorship contribution statement

**Victoria C. Nolan:** Conceptualization, Formal analysis, Supervision. **Laia Rafols:** Formal analysis, Methodology. **James Harrison:** Formal analysis, Supervision. **Joan J. Soldevila-Barreda:** Formal analysis, Methodology, Supervision. **Marialuisa Crosatti:** . **Natalie J. Garton:** . **Malgorzata Wegrzyn:** . **Danielle L. Timms:** Supervision. **Colin C. Seaton:** Formal analysis, Methodology. **Helen Sendron:** Methodology. **Maria Azmanova:** Methodology. **Nicolas P.E. Barry:** Conceptualization, Funding acquisition, Supervision. **Anaïs Pitto-Barry:** Formal analysis, Supervision. **Jonathan A.G. Cox:** Conceptualization, Funding acquisition, Supervision.

## Declaration of Competing Interest

Nicolas Barry, Anaïs Pitto-Barry, Joan Soldevila-Barreda, and Jonathan Cox have patent #International PCT Application No. PCT/GB2021/050471 pending to Aston University and University of Bradford.

## Appendix Supplementary materials

Image, application 1
