# Supplementary material for: Indole-containing arene-ruthenium complexes with broad spectrum activity against antibiotic-resistant bacteria
Source: Curr Res Microb Sci. 2021 Dec 16;3:100099. doi: 10.1016/j.crmicr.2021.100099 (PMC8760505; doi:10.1016/j.crmicr.2021.100099)
Supplement: Supplementary file 1 [file mmc1.docx]

Supplementary Material for

Indole-containing arene-ruthenium complexes with broad scope of antibacterial activity against Gram-negative antibiotic-resistant bacteria

Victoria C. Nolan^1‡^, Laia Rafols^2‡^, James Harrison^1^, Joan J. Soldevila-Barreda^2^, Marialuisa Crosatti^3^, Natalie J. Garton^4^, Malgorzata Wegrzyn^3^, Danielle L. Timms,^1^ Colin C. Seaton,^2^ Helen Sendron^2^, Maria Azmanova^2^, Nicolas P. E. Barry^2^, Anaïs Pitto-Barry^2,5^*, Jonathan A. G. Cox^1^*

^1^ College of Health and Life Sciences, Aston University, B4 7ET, Birmingham, United Kingdom

^2^ School of Chemistry and Biosciences, University of Bradford, BD7 1DP, Bradford, United Kingdom

^3^CL3 facility, Division of Biomedical Services, University of Leicester, LE1 7RH, Leicester, United Kingdom

^4^Department of Respiratory Sciences and Leicester TB Research Group, University of Leicester, LE1 7RH, Leicester, United Kingdom

^5^ Université Paris-Saclay, CNRS, Institut Galien Paris-Saclay, 92296 Châtenay-Malabry, France

Figures S1 – S24: ^1^H and ^13^C NMR spectra of ligands **L2** – **L9** and complexes **1** – **4**

Figures S25 – S36: High-resolution ESI-MS spectra of ligands **L2** – **L9** and complexes **1** – **4**

Figures S37 – S38: IC_50_ graphs for ligands **L6** – **L9** and complexes **1** – **4** against PNT2, A2780, and A2780cisR cell lines

Figure S39: Minimum bactericidal concentrations for Complexes **1** – **4**.

Figure S40: X-ray structures of ligand **L9** and Ru^II^ complexes **2** – **4**.

Tables S1 – S12: Crystallographic data of ligand **L9** and complexes **2** – **4**.


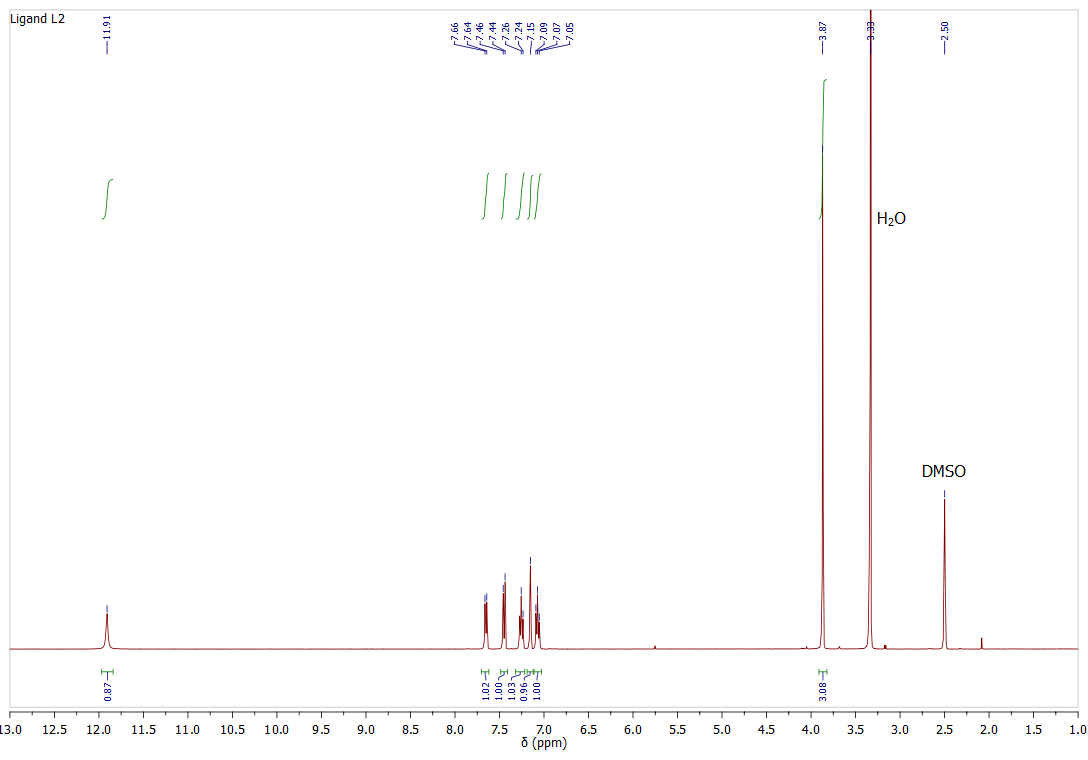


Figure S1. ^1^H NMR spectrum (400 MHz, DMSO-d_6_) of ligand L2


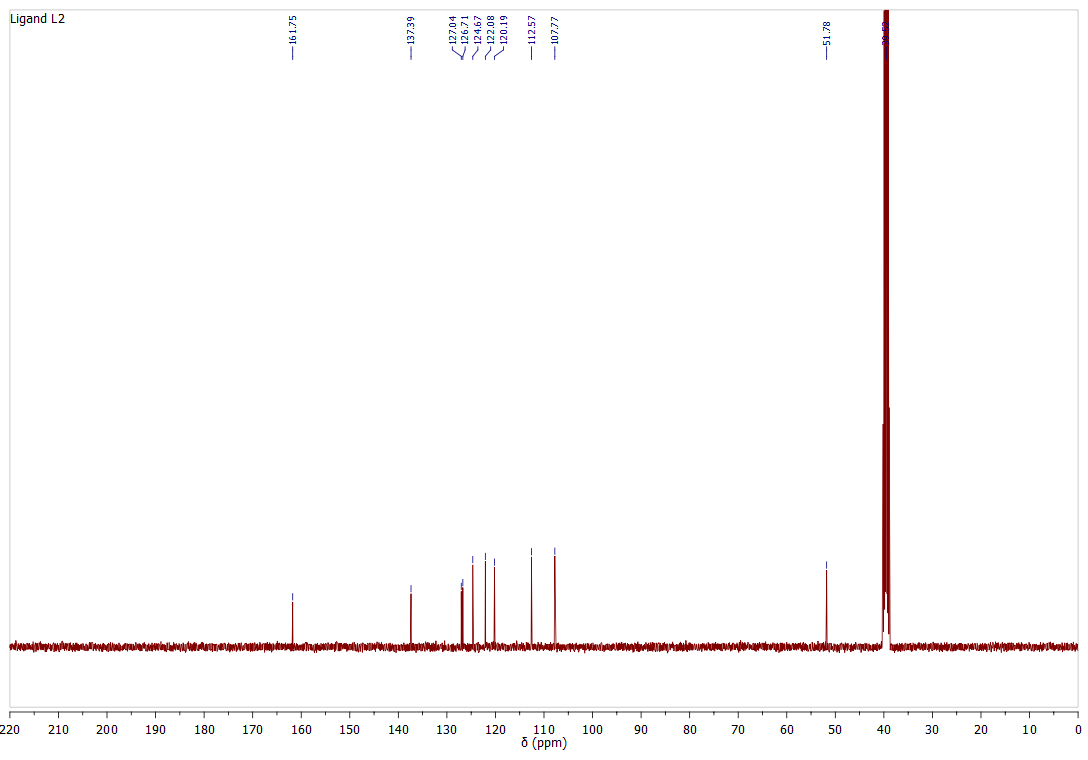


Figure S2. ^13^C NMR spectrum (100 MHz, DMSO-d_6_) of ligand L2


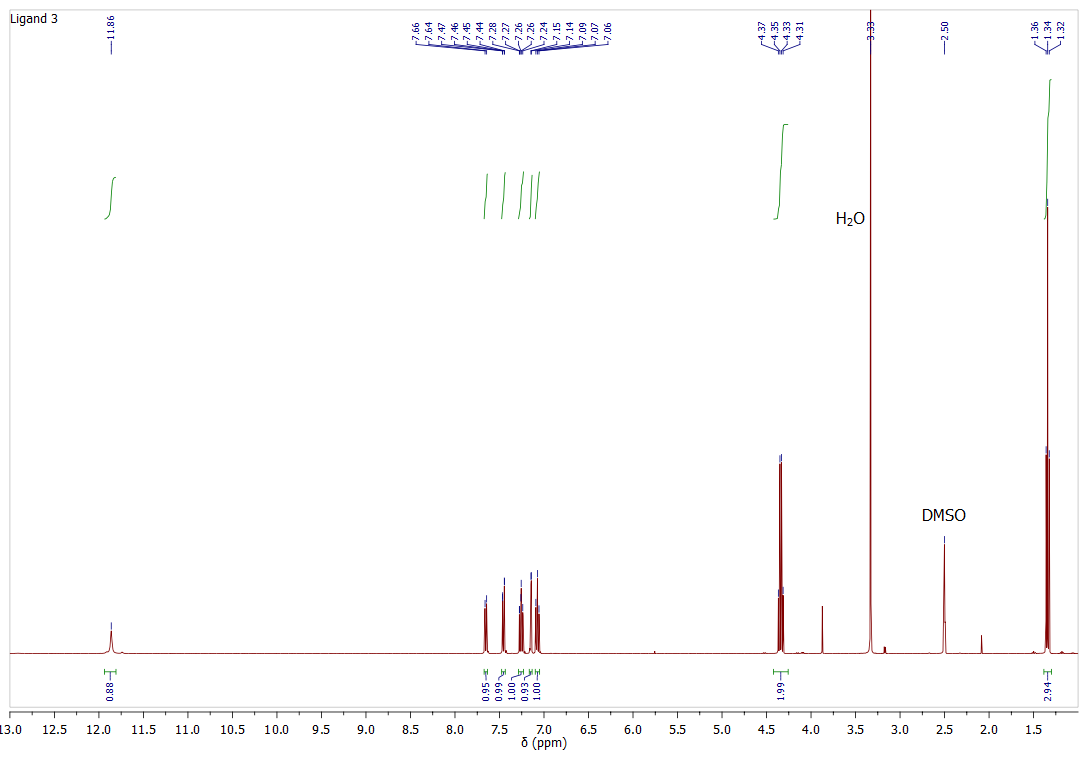


Figure S3. ^1^H NMR spectrum (400 MHz, DMSO-d_6_) of ligand L3


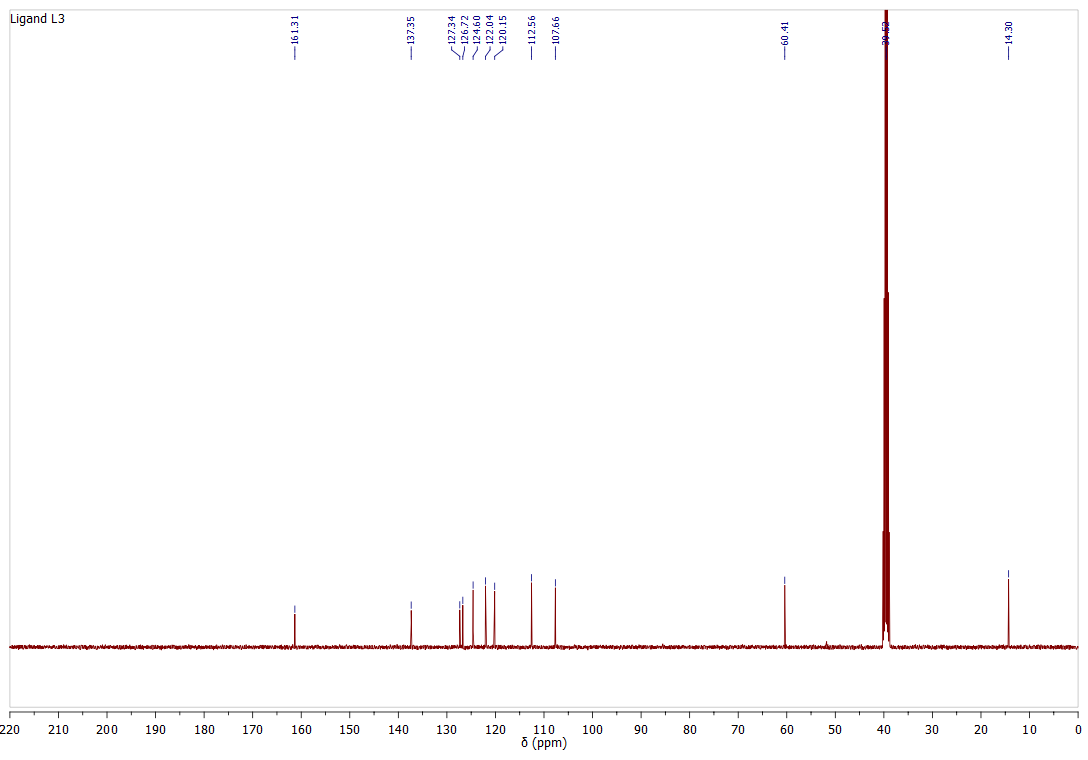


Figure S4. ^13^C NMR spectrum (100 MHz, DMSO-d_6_) of ligand L3


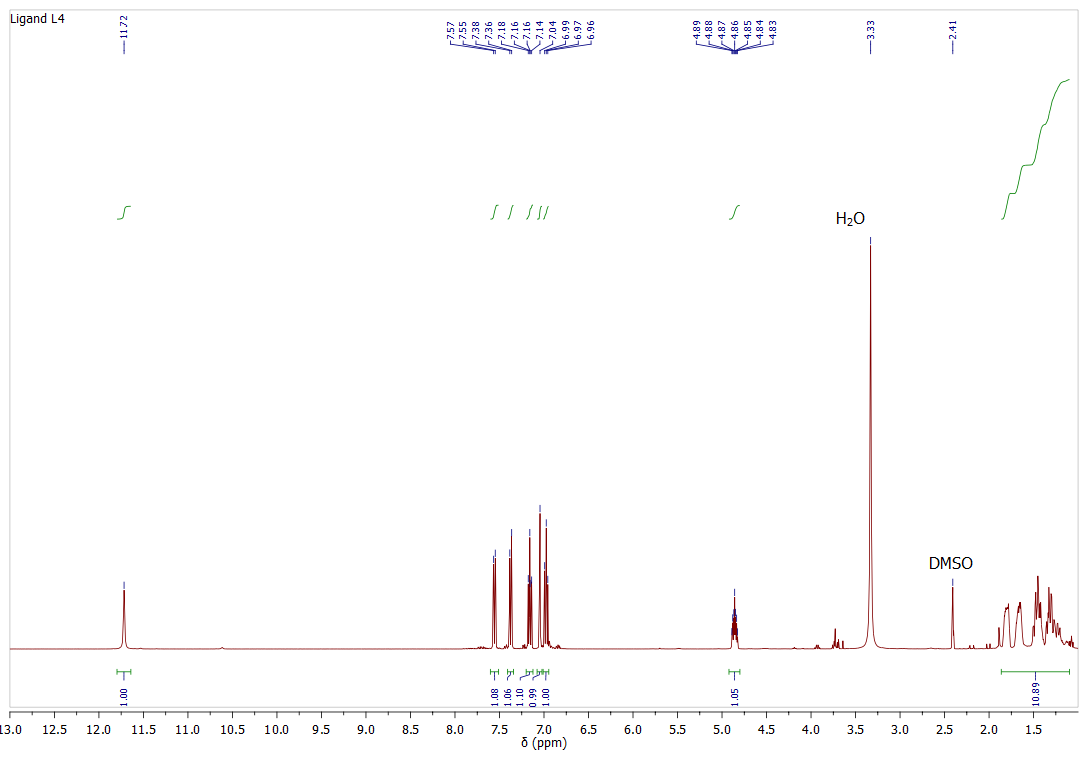


Figure S5. ^1^H NMR spectrum (400 MHz, DMSO-d_6_) of ligand L4


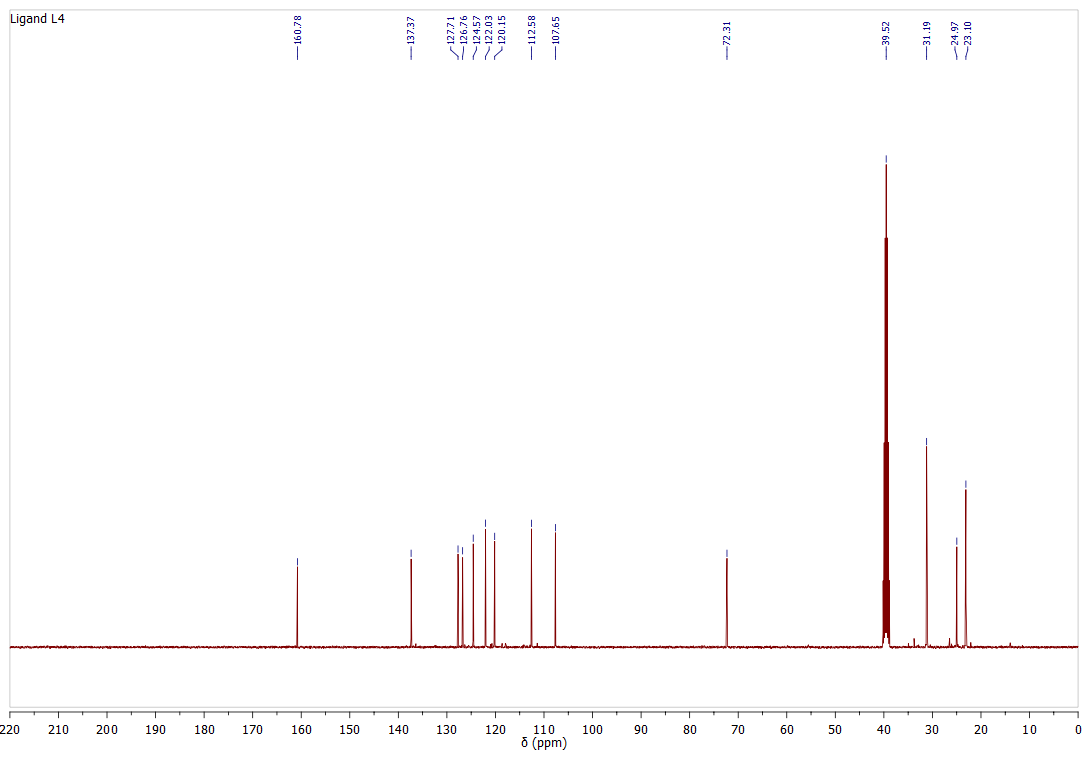


Figure S6. ^13^C NMR spectrum (100 MHz, DMSO-d_6_) of ligand L4


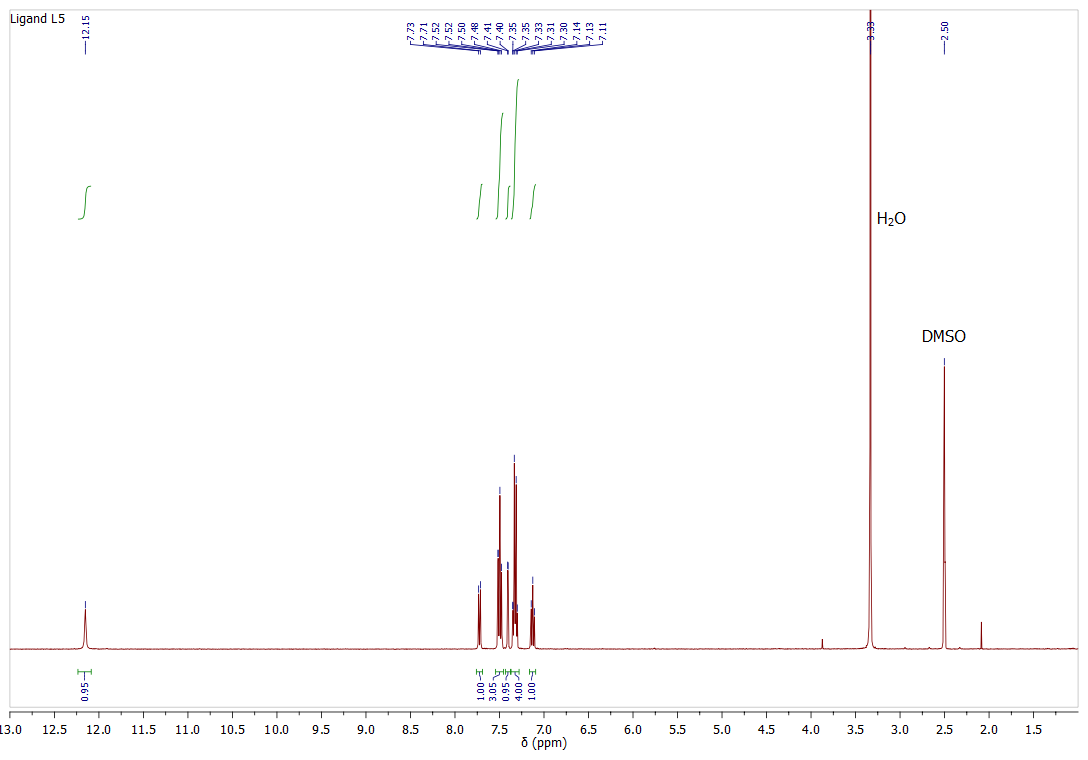


Figure S7. ^1^H NMR spectrum (400 MHz, DMSO-d_6_) of ligand L5


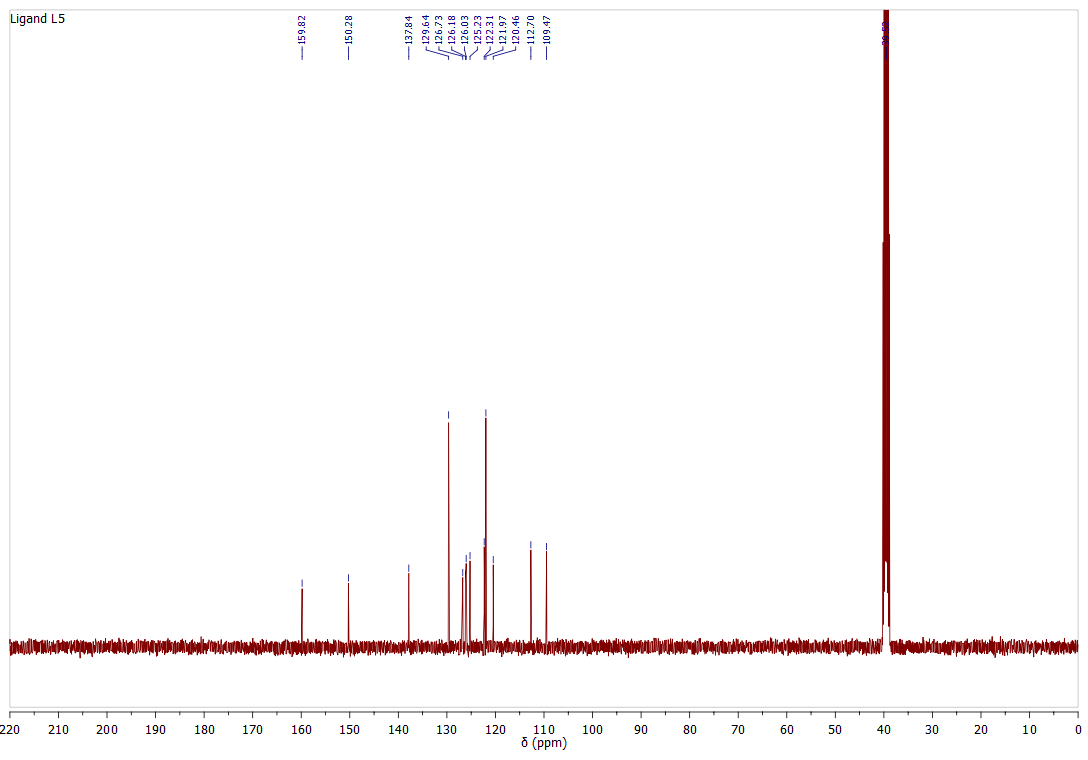


Figure S8. ^13^C NMR spectrum (100 MHz, DMSO-d_6_) of ligand L5


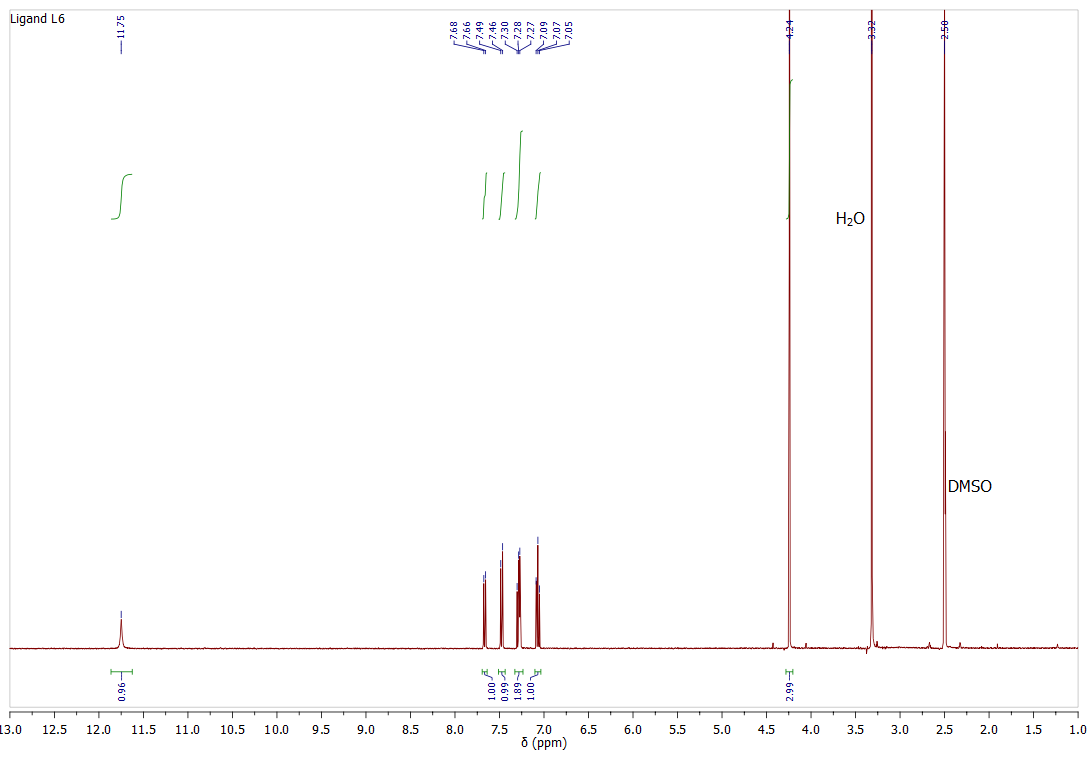


Figure S9. ^1^H NMR spectrum (400 MHz, DMSO-d_6_) of ligand L6


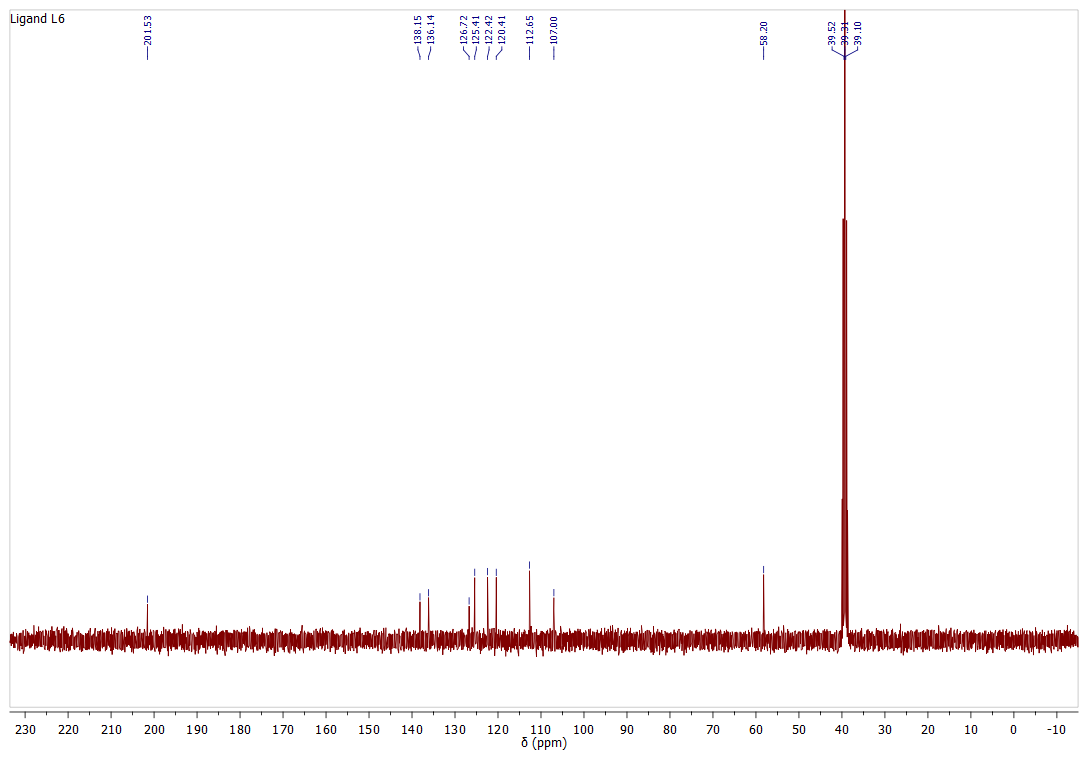


Figure S10. ^13^C NMR spectrum (100 MHz, DMSO-d_6_) of ligand L6


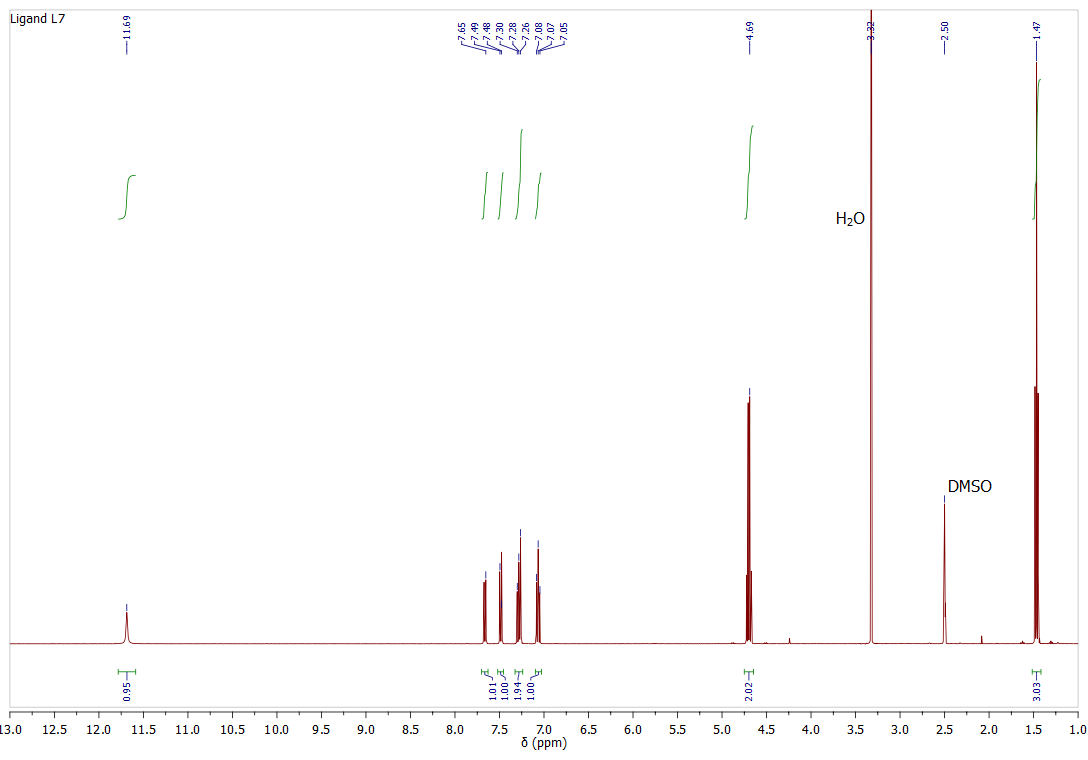


Figure S11. ^1^H NMR spectrum (400 MHz, DMSO-d_6_) of ligand L7


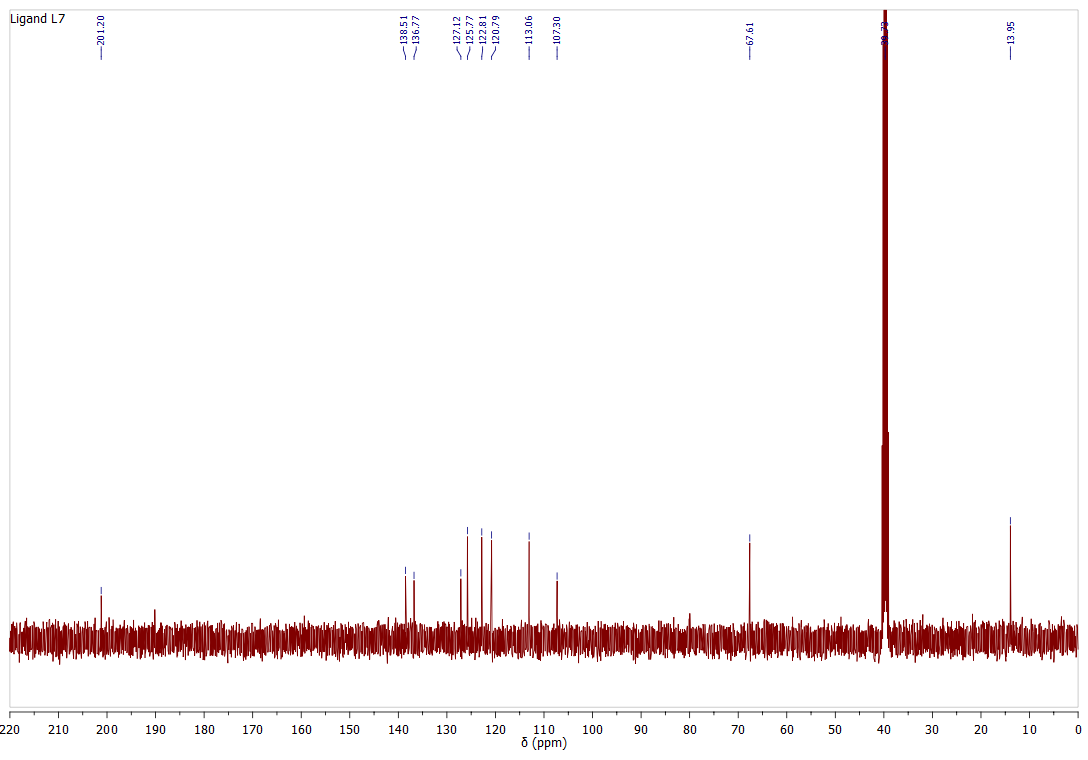


Figure S12. ^13^C NMR spectrum (100 MHz, DMSO-d_6_) of ligand L7


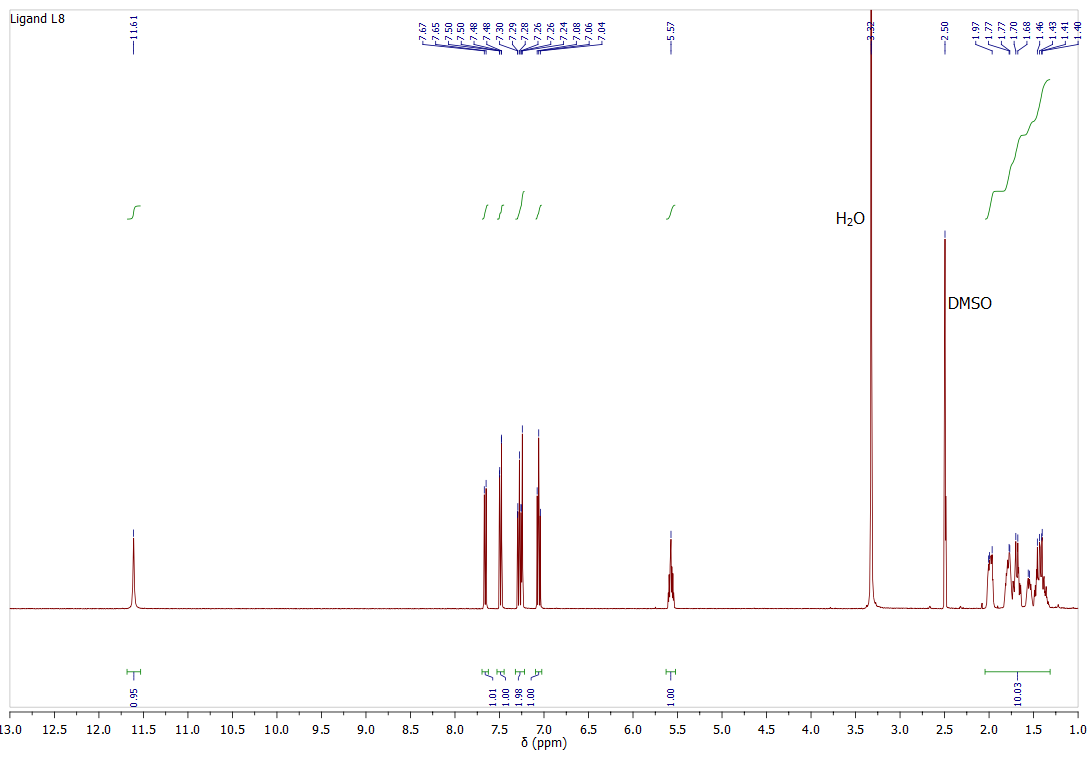


Figure S13. ^1^H NMR spectrum (400 MHz, DMSO-d_6_) of ligand L8


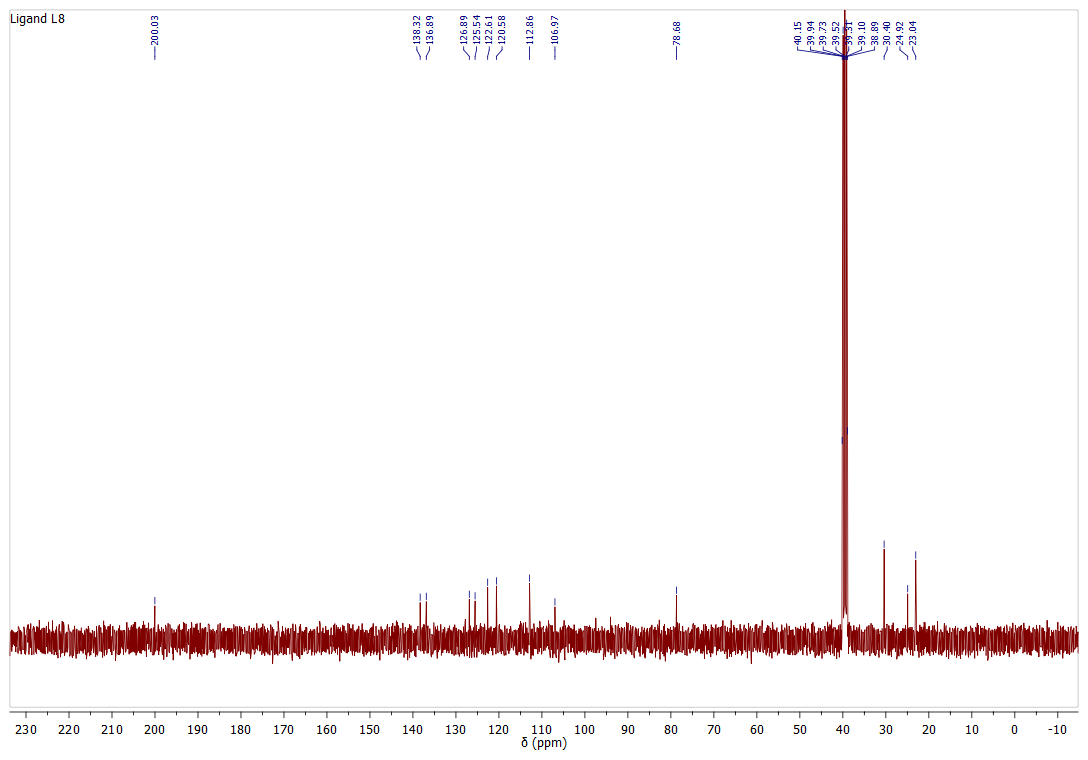


Figure S14. ^13^C NMR spectrum (100 MHz, DMSO-d_6_) of ligand L8


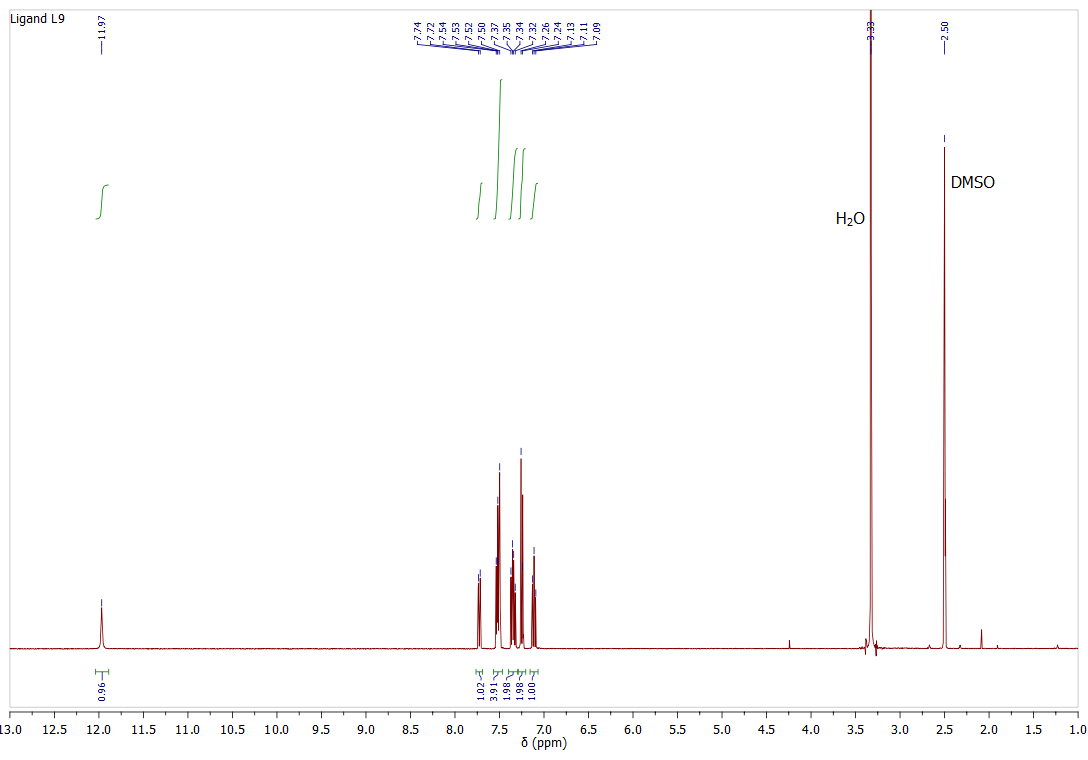


Figure S15. ^1^H NMR spectrum (400 MHz, DMSO-d_6_) of ligand L9


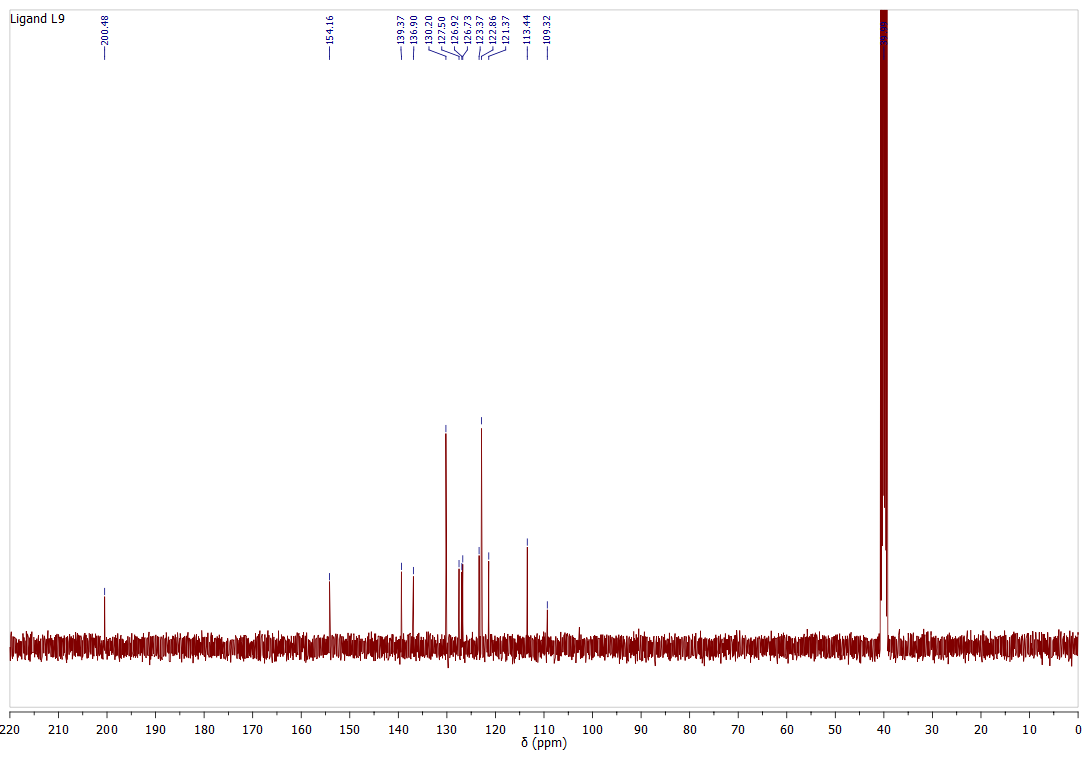


Figure S16. DEPT NMR spectrum (100 MHz, DMSO-d_6_) of ligand L9


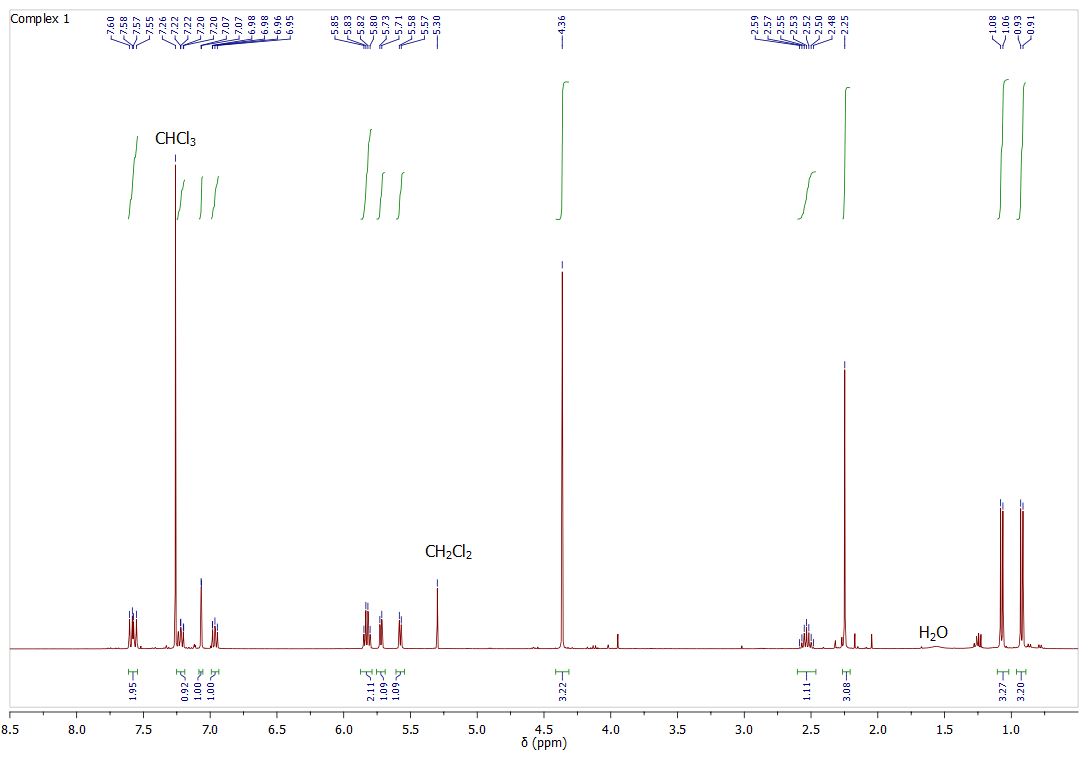


Figure S17. ^1^H NMR spectrum (400 MHz, CDCl_3_) of complex **1**

**
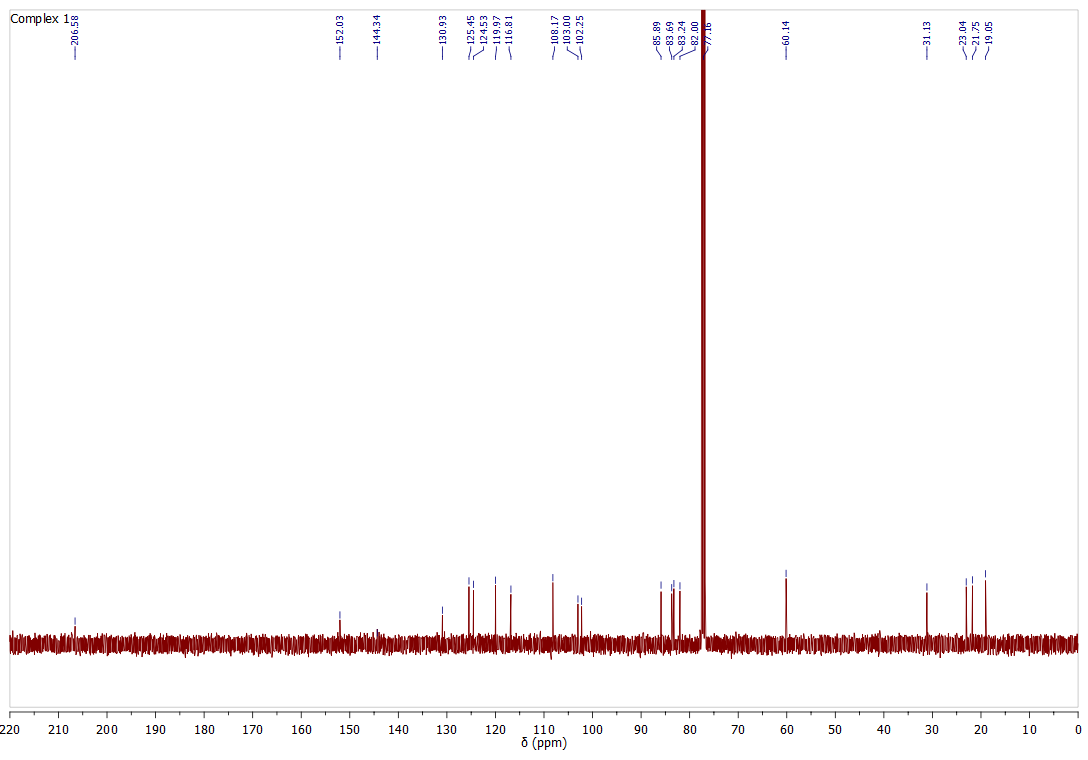
**

Figure S18. ^13^C NMR spectrum (100 MHz, CDCl_3_) of complex **1**


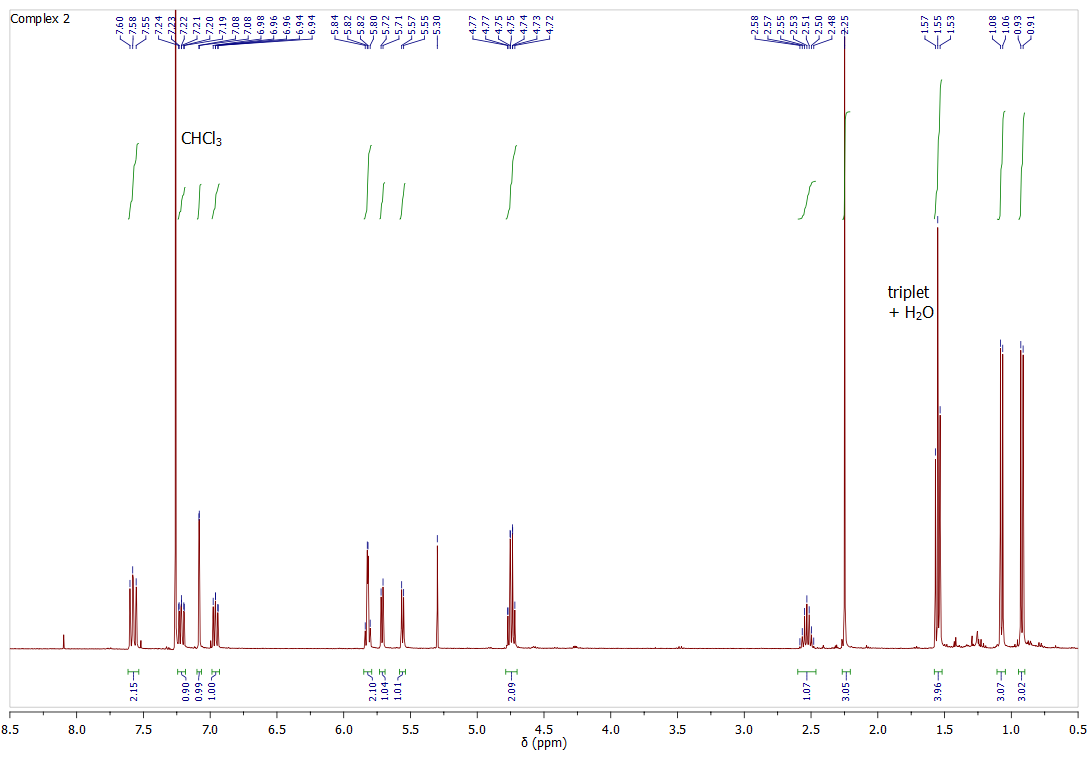


Figure S19. ^1^H NMR spectrum (400 MHz, CDCl_3_) of complex **2**

**
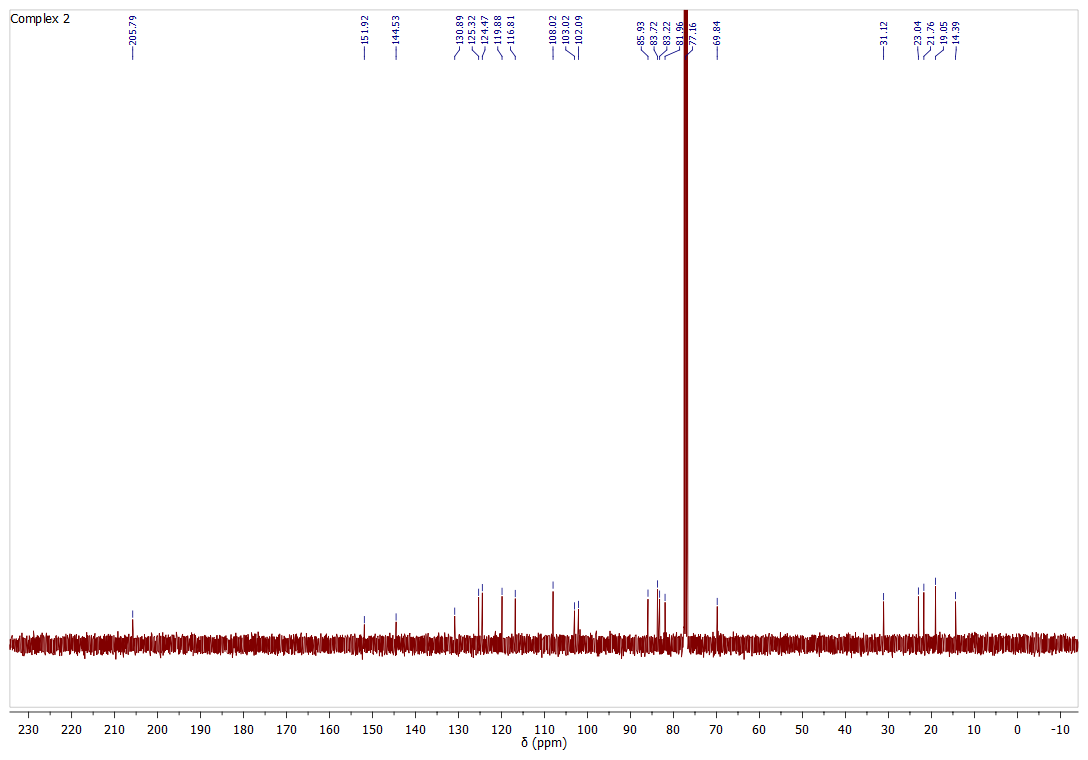
**

Figure S20. ^13^C NMR spectrum (100 MHz, CDCl_3_) of complex **2**

**
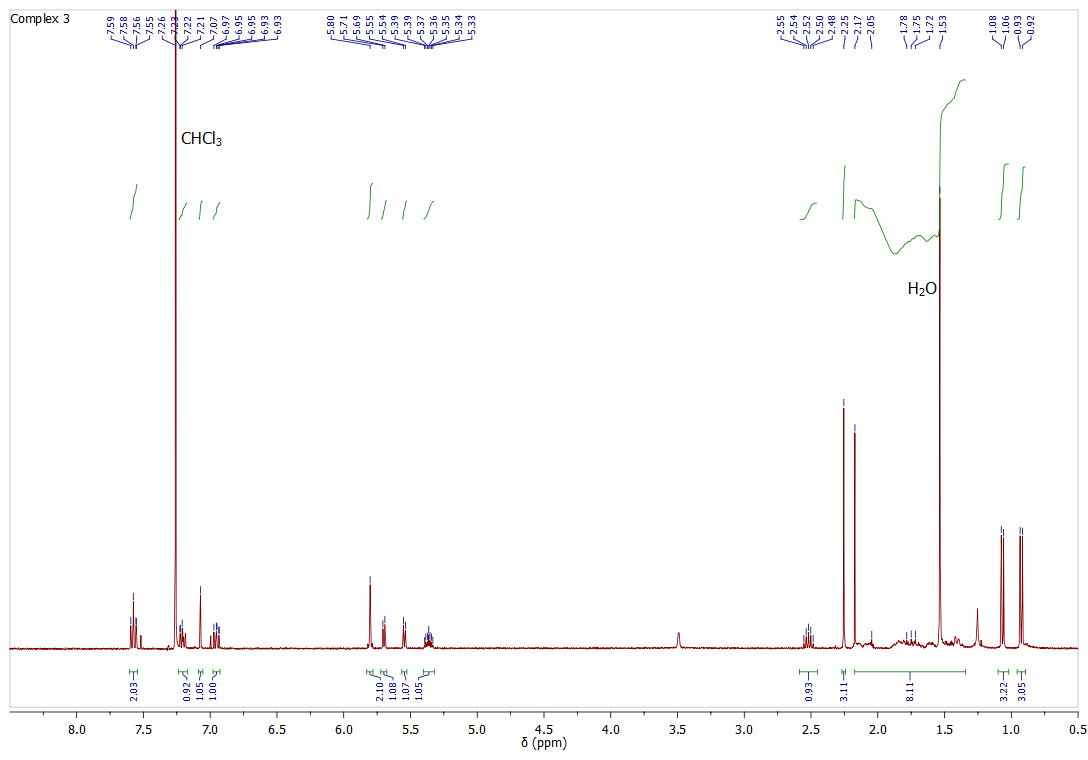
**

Figure S21. ^1^H NMR spectrum (400 MHz, CDCl_3_) of complex **3**


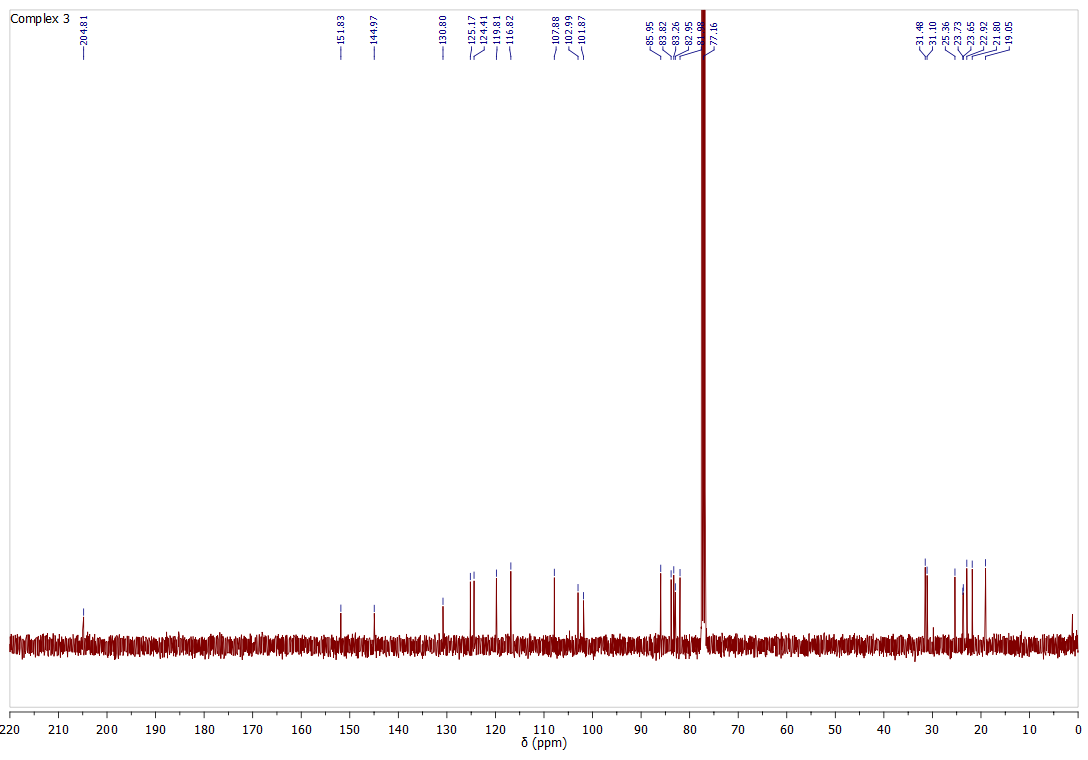


Figure S22. ^13^C NMR spectrum (100 MHz, CDCl_3_) of complex **3**

**
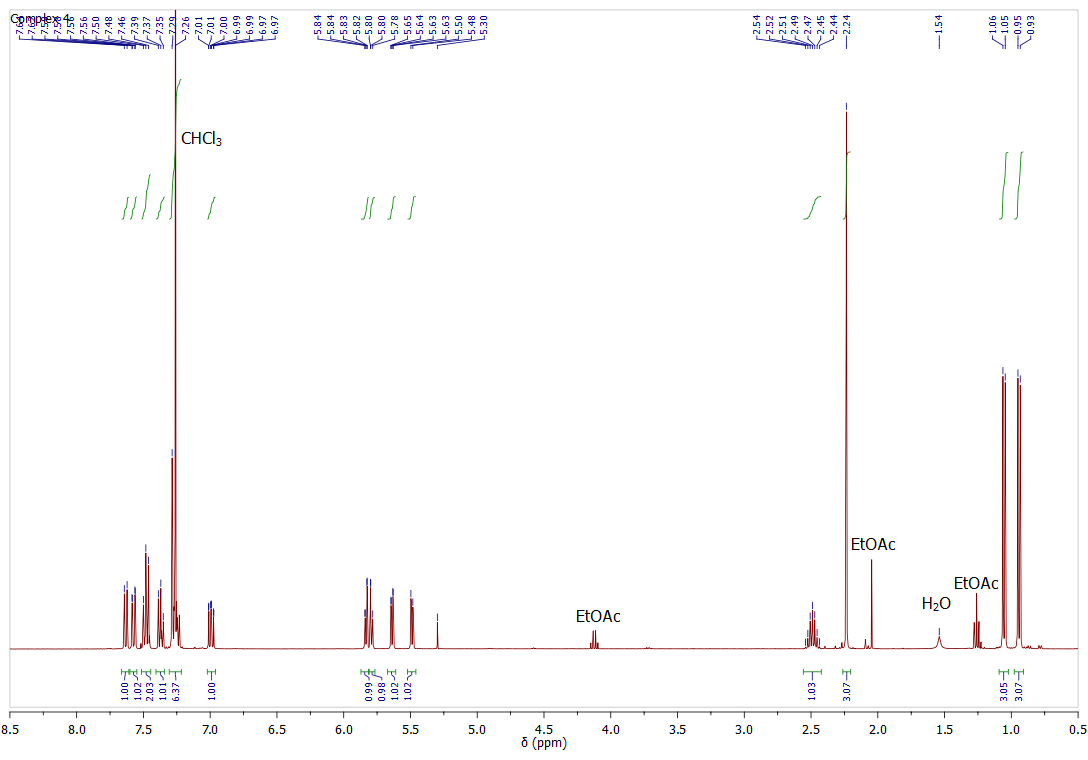
**

Figure S23. ^1^H NMR spectrum (400 MHz, CDCl_3_) of complex **4**


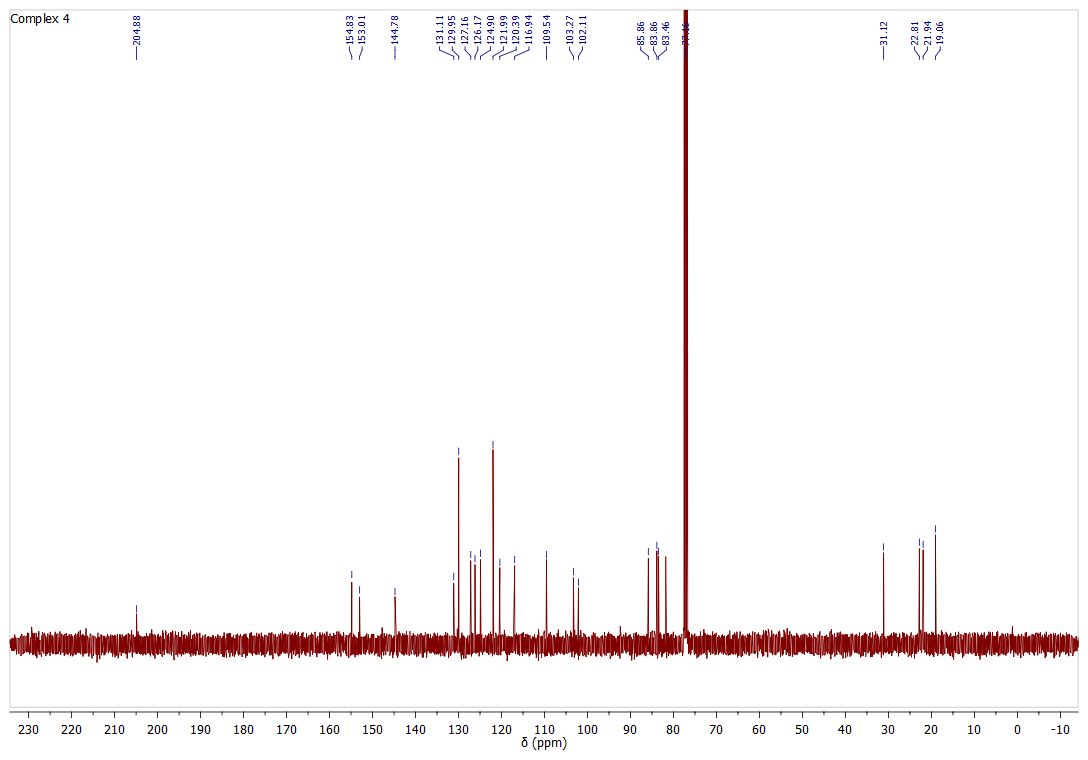


Figure S24. ^13^C NMR spectrum (100 MHz, CDCl_3_) of complex **4**

**
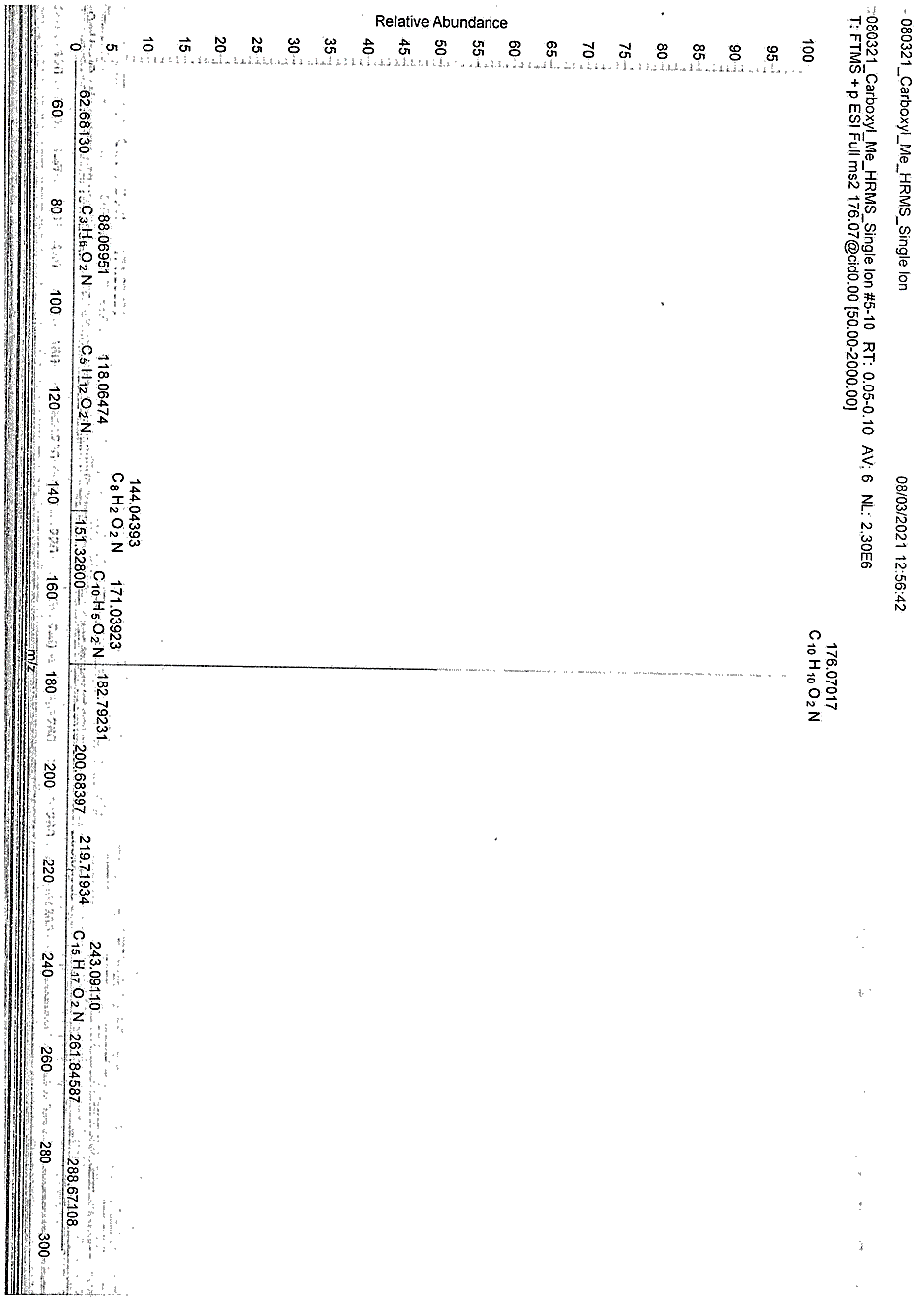
**

Figure S25. HRMS spectrum of ligand L2


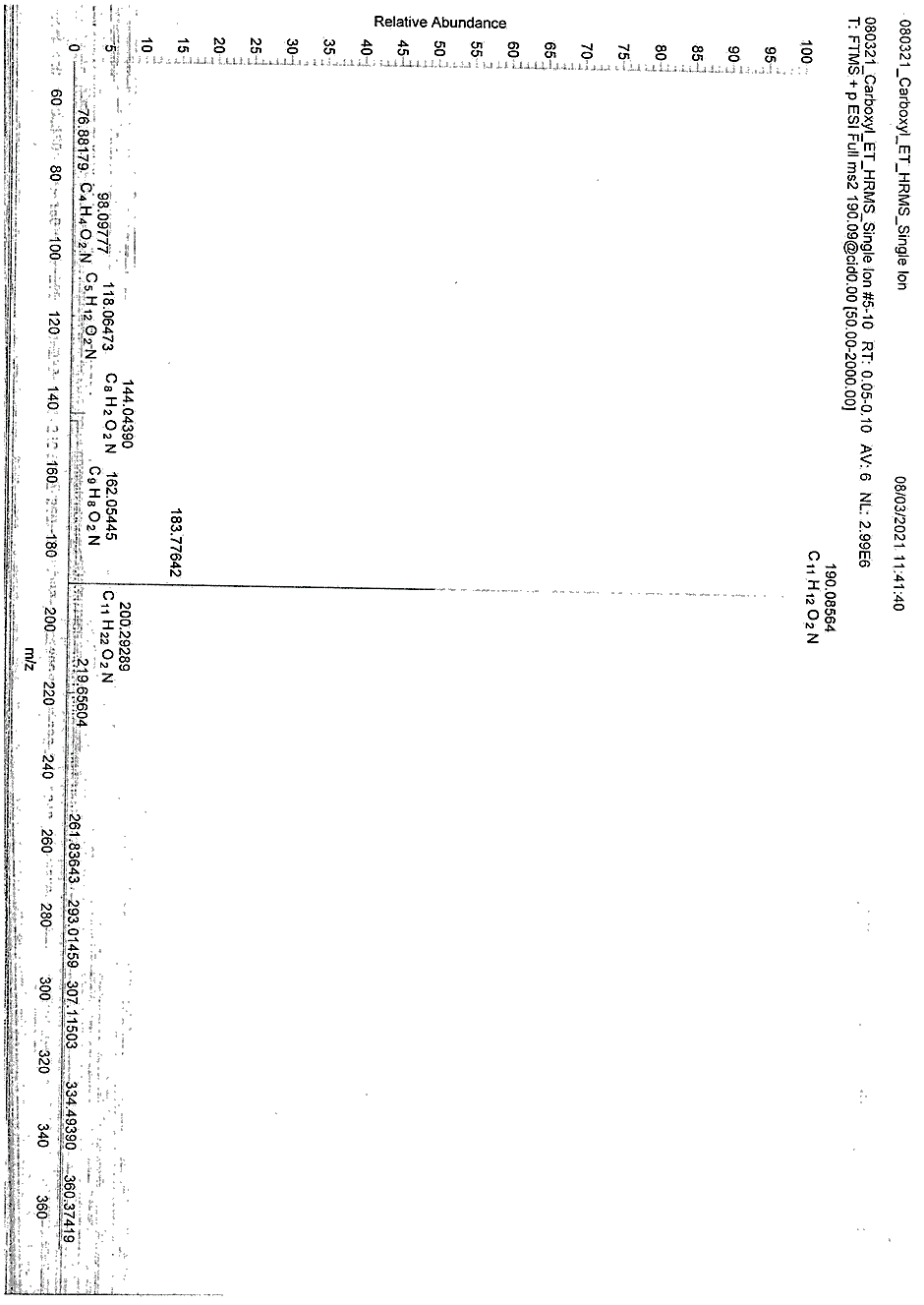


Figure S26. HRMS spectrum of ligand L3


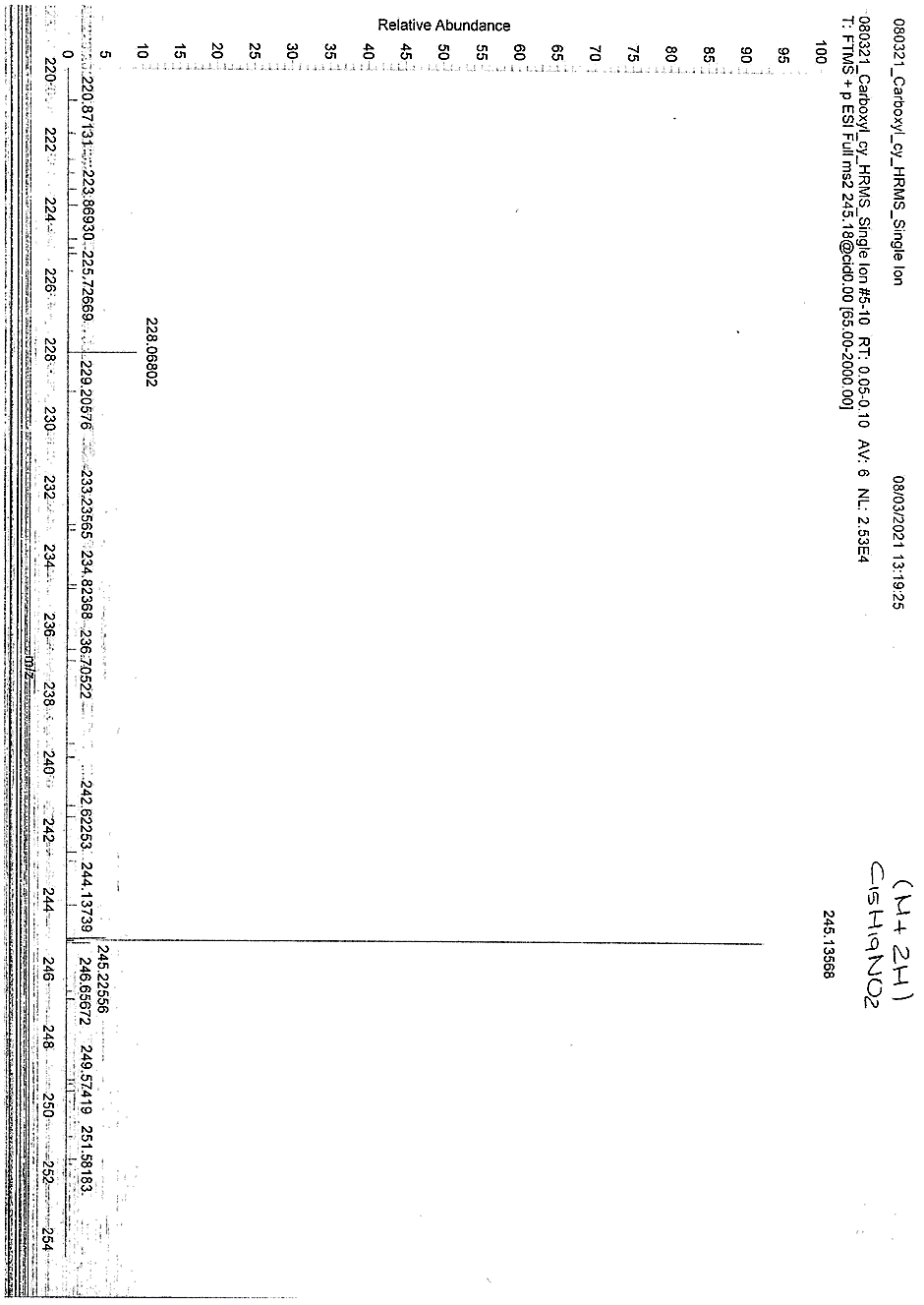


Figure S27. HRMS spectrum of ligand L4


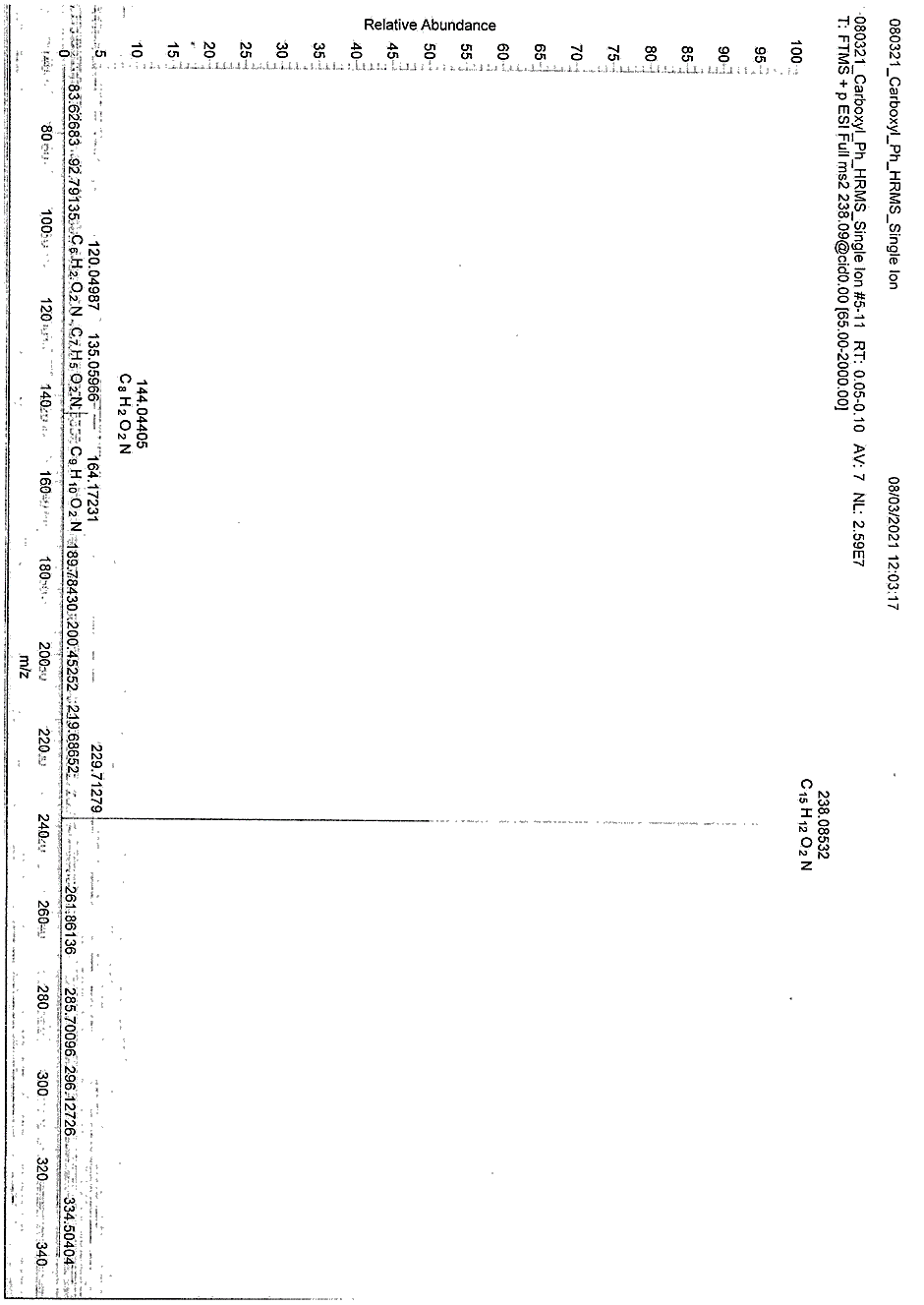


Figure S28. HRMS spectrum of ligand L5


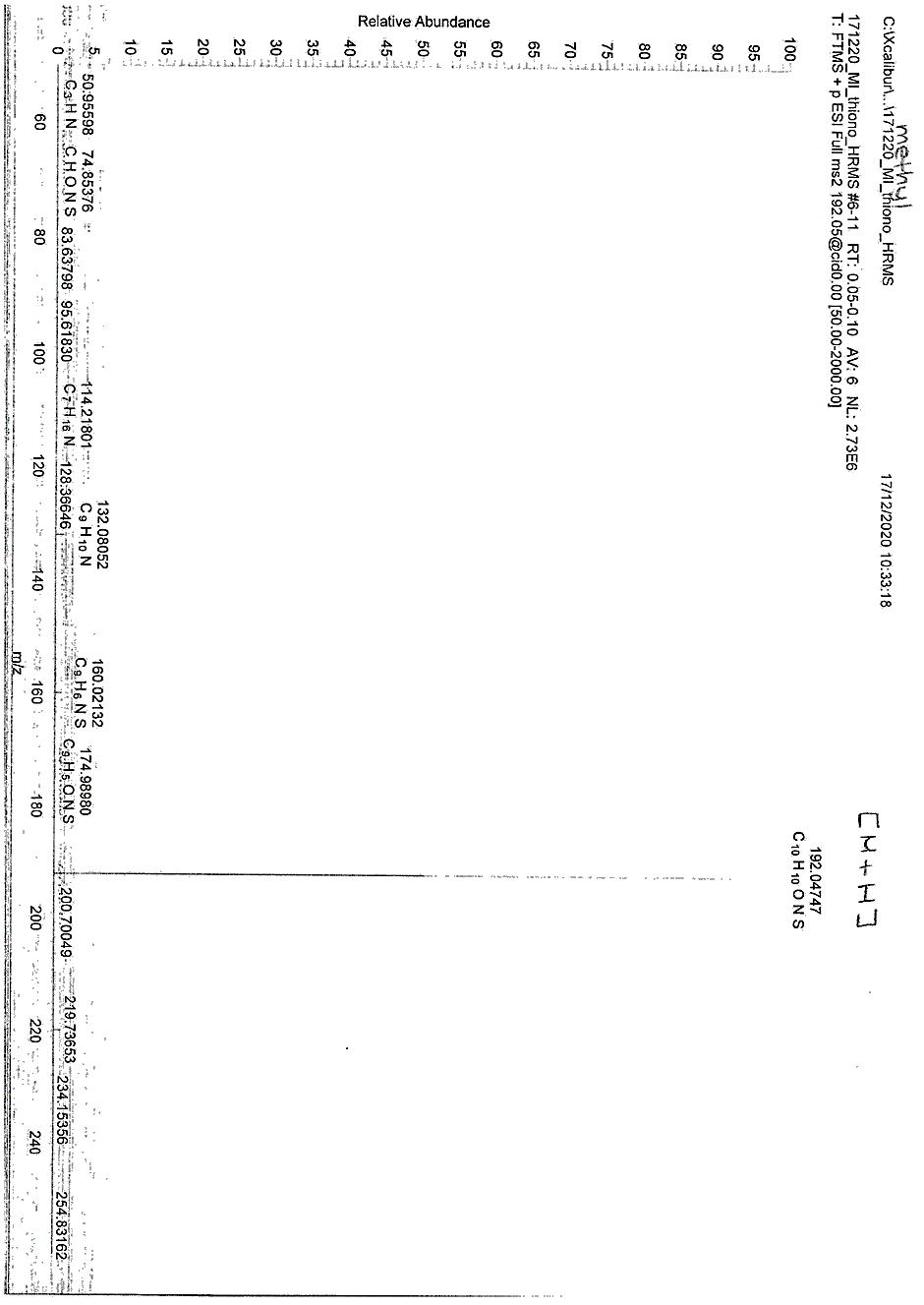


Figure S29. HRMS spectrum of ligand L6


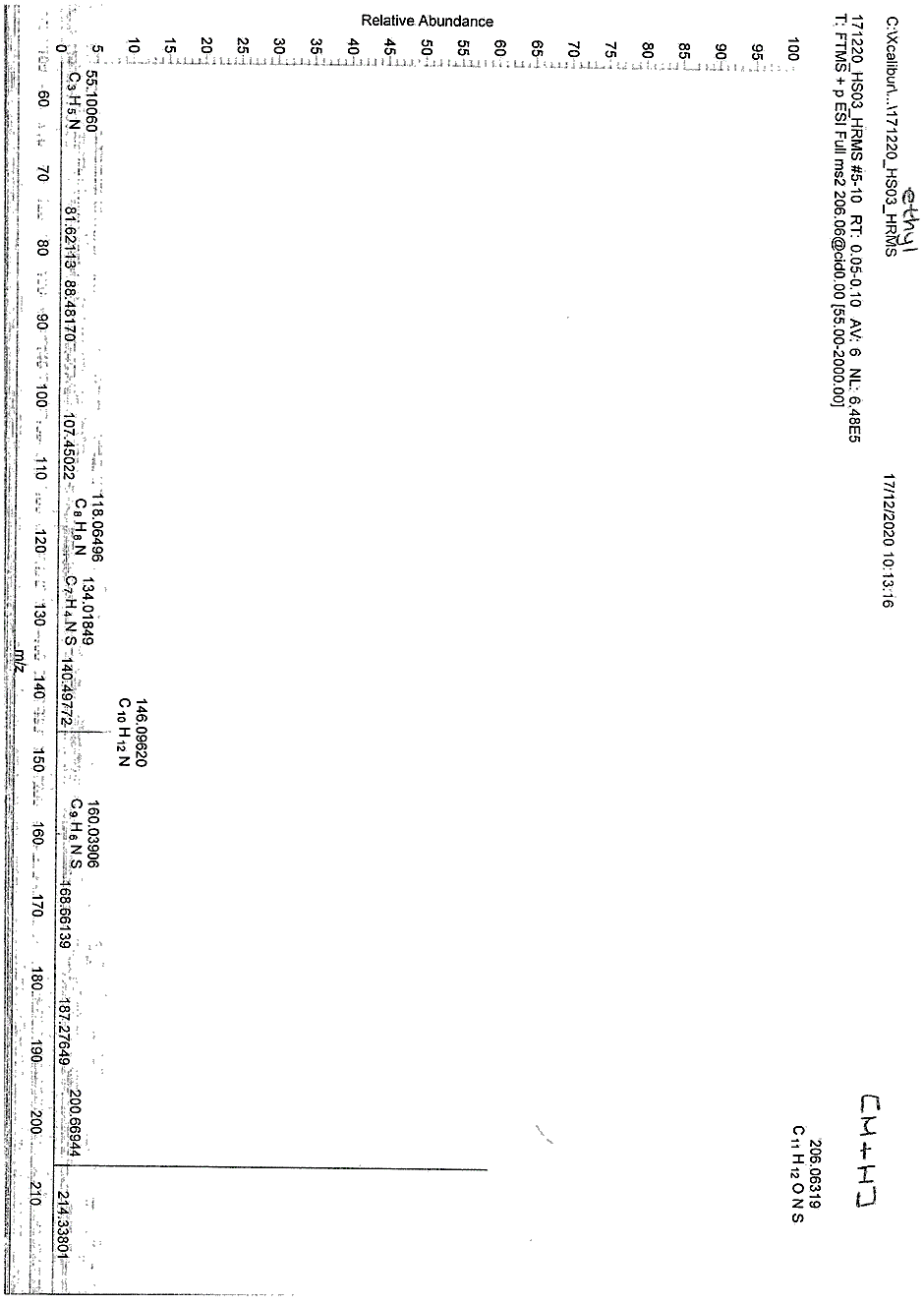


Figure S30. HRMS spectrum of ligand L7


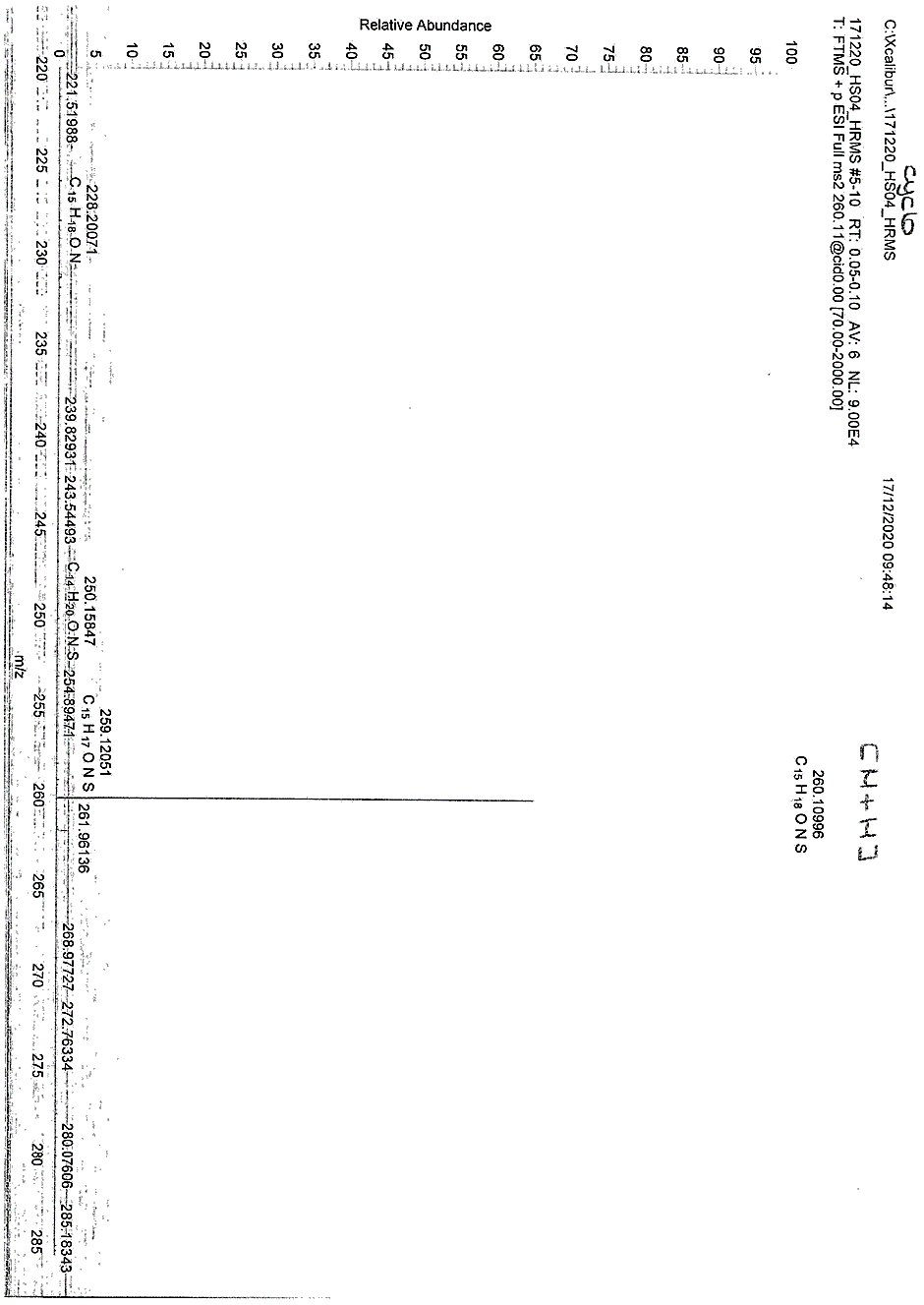


Figure S31. HRMS spectrum of ligand L8


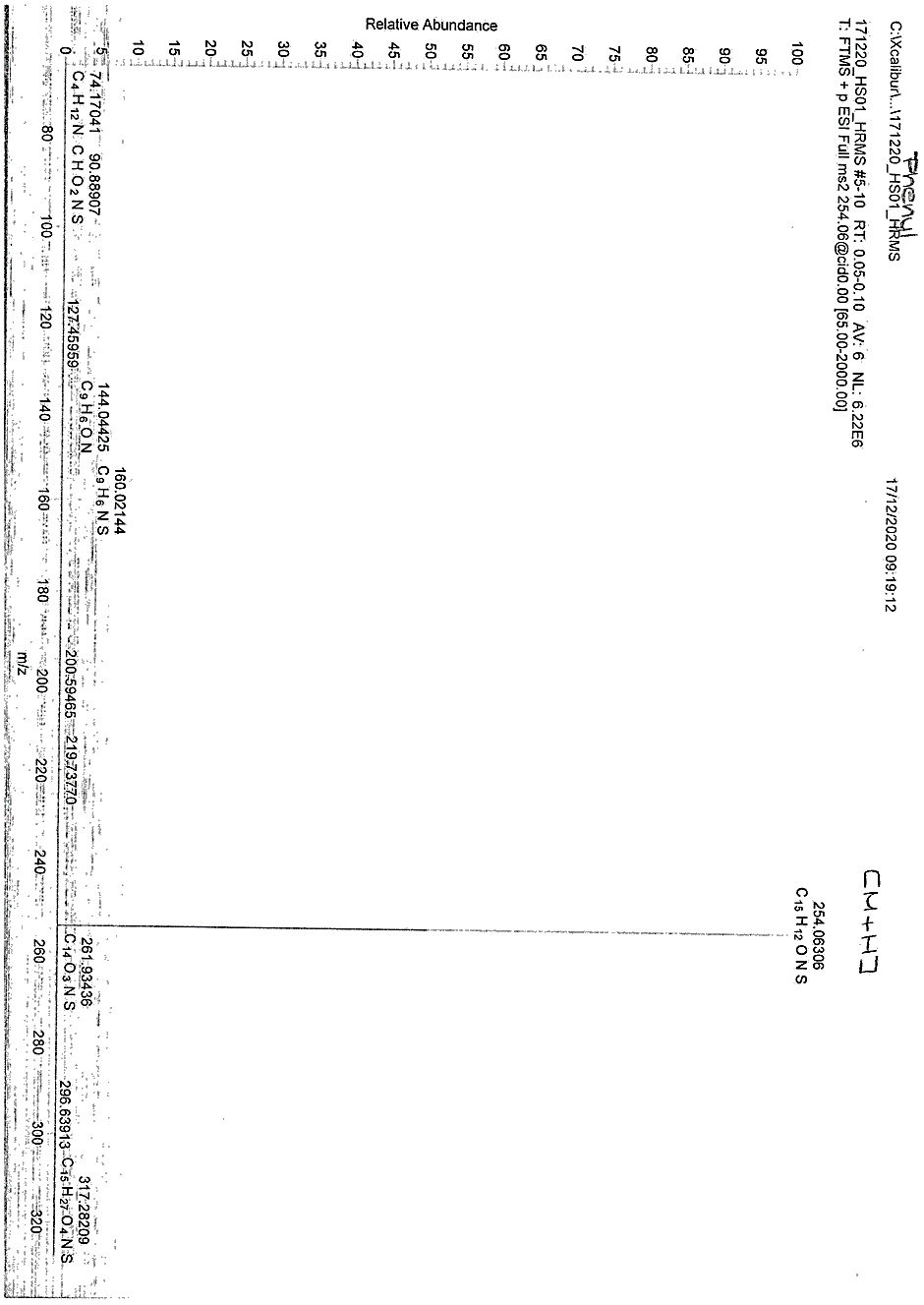


Figure S32. HRMS spectrum of ligand L9


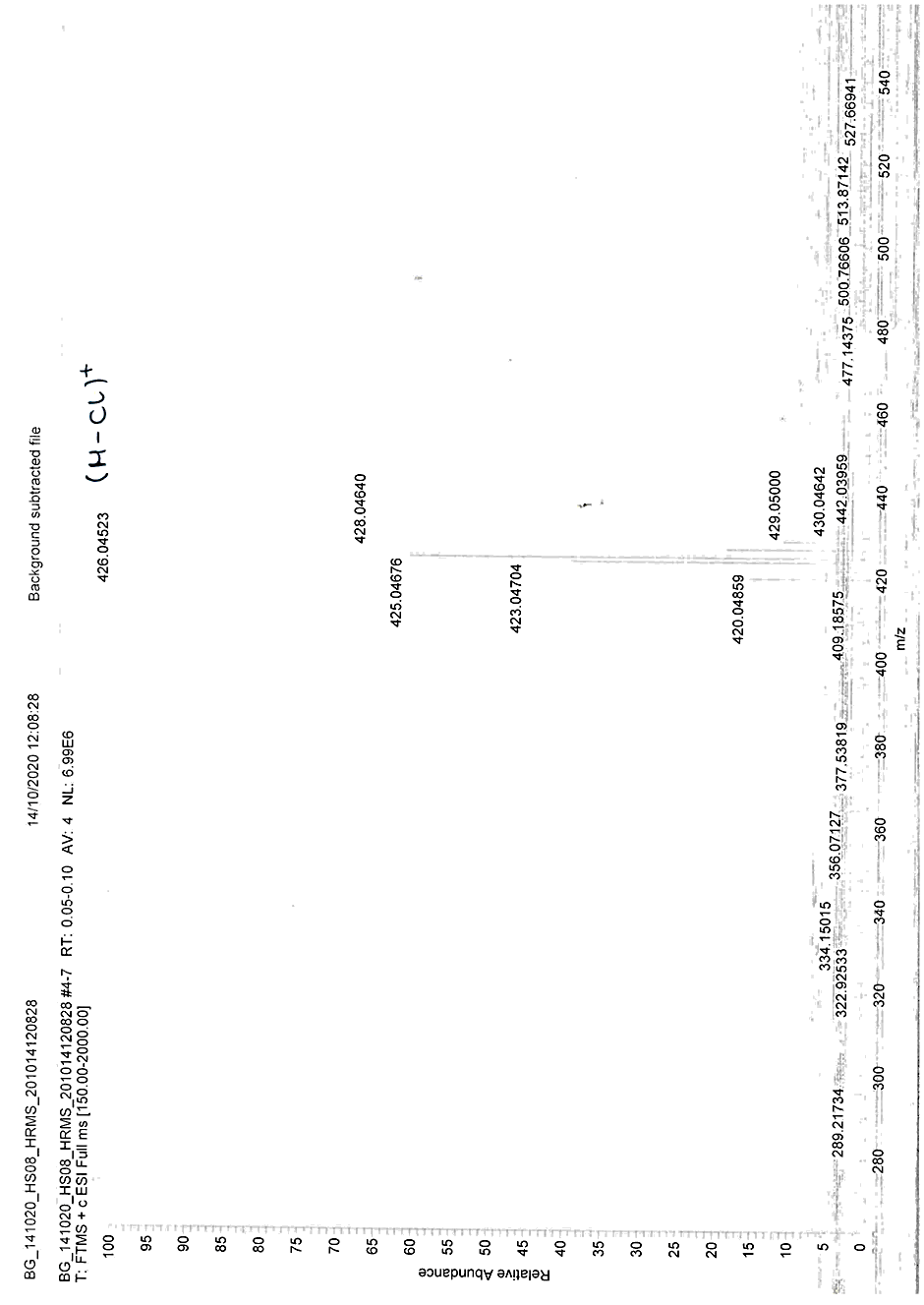


Figure S33. HRMS spectrum of complex **1**


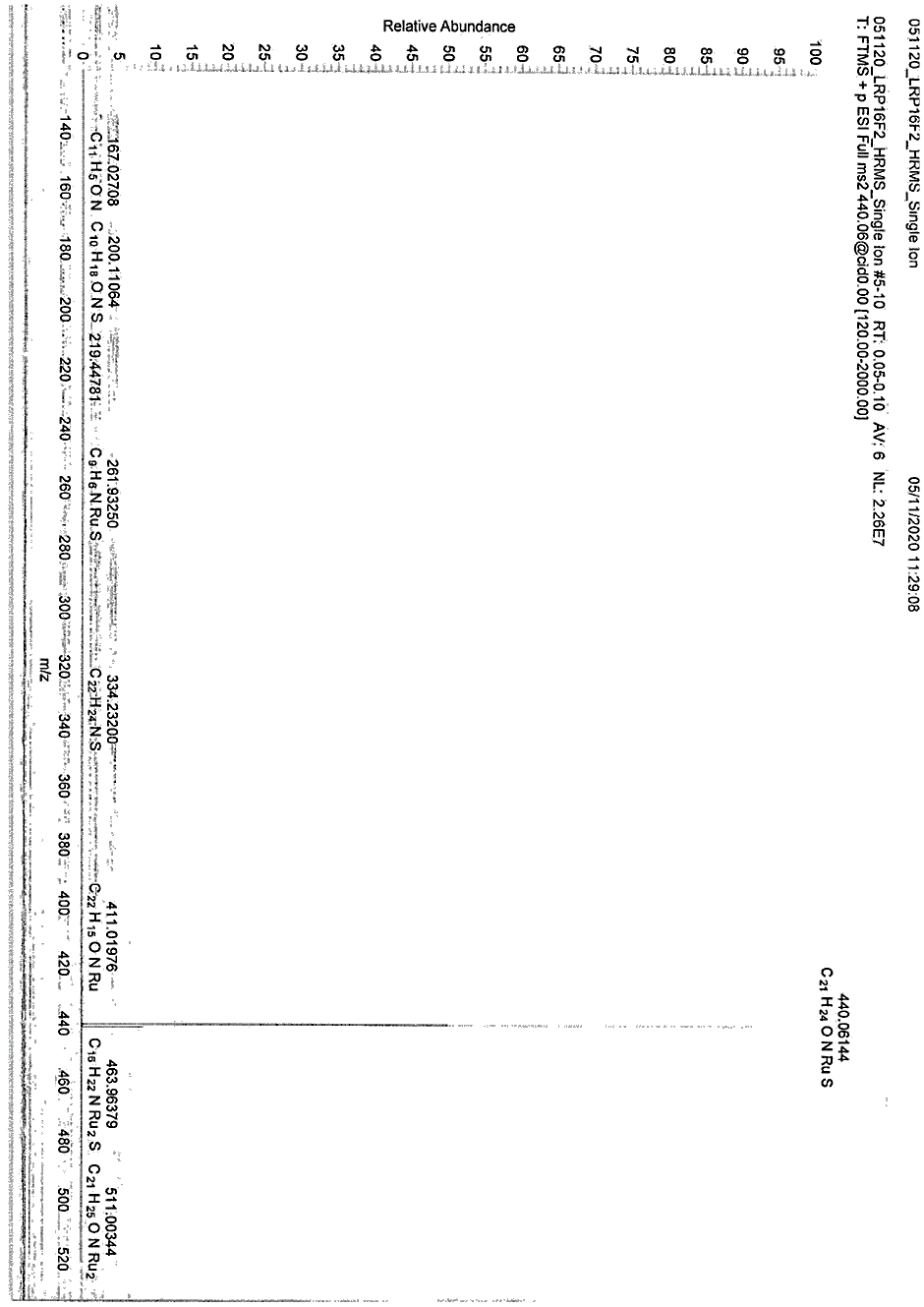


Figure S34. HRMS spectrum of complex **2**


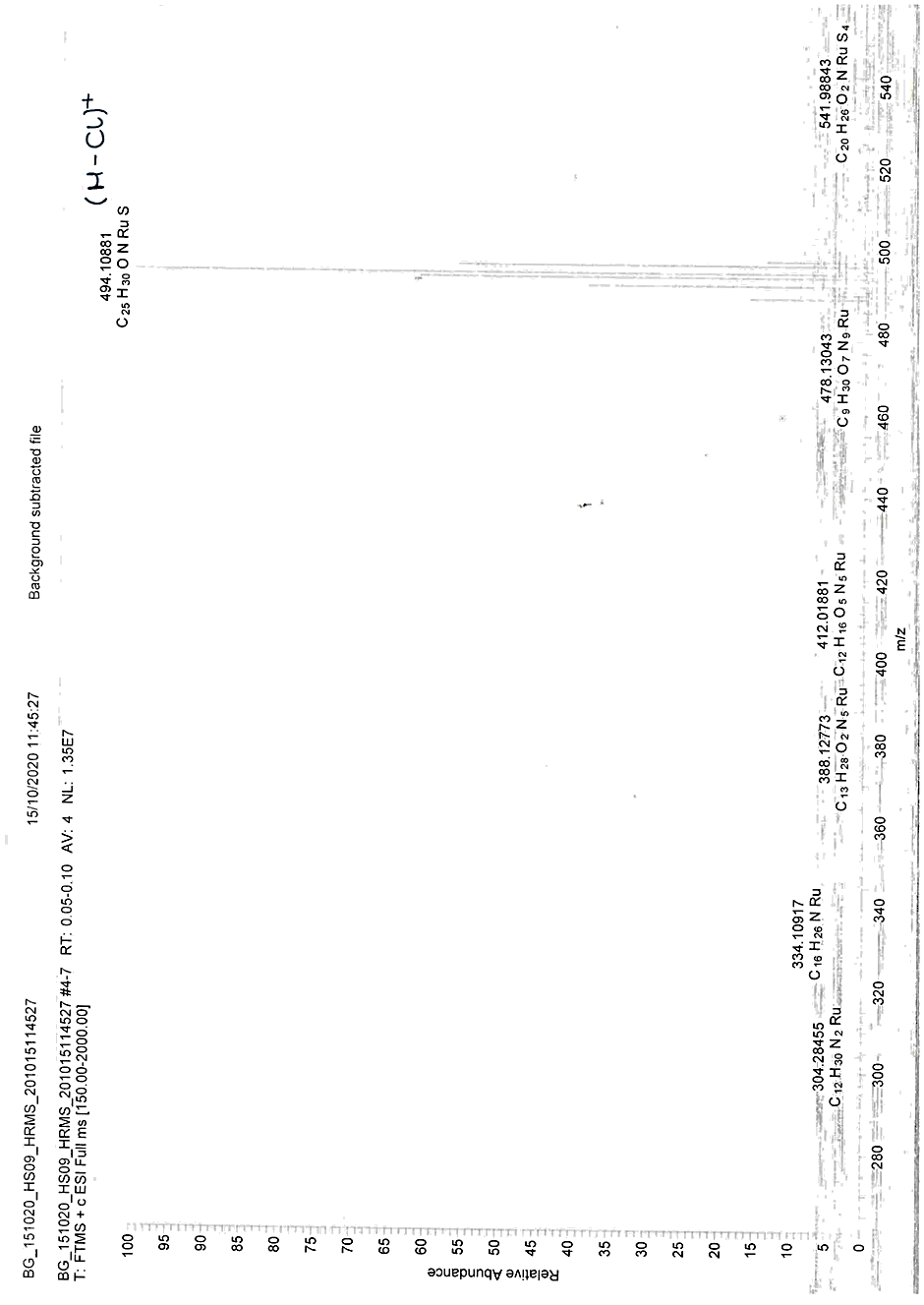


Figure S35. HRMS spectrum of complex **3**


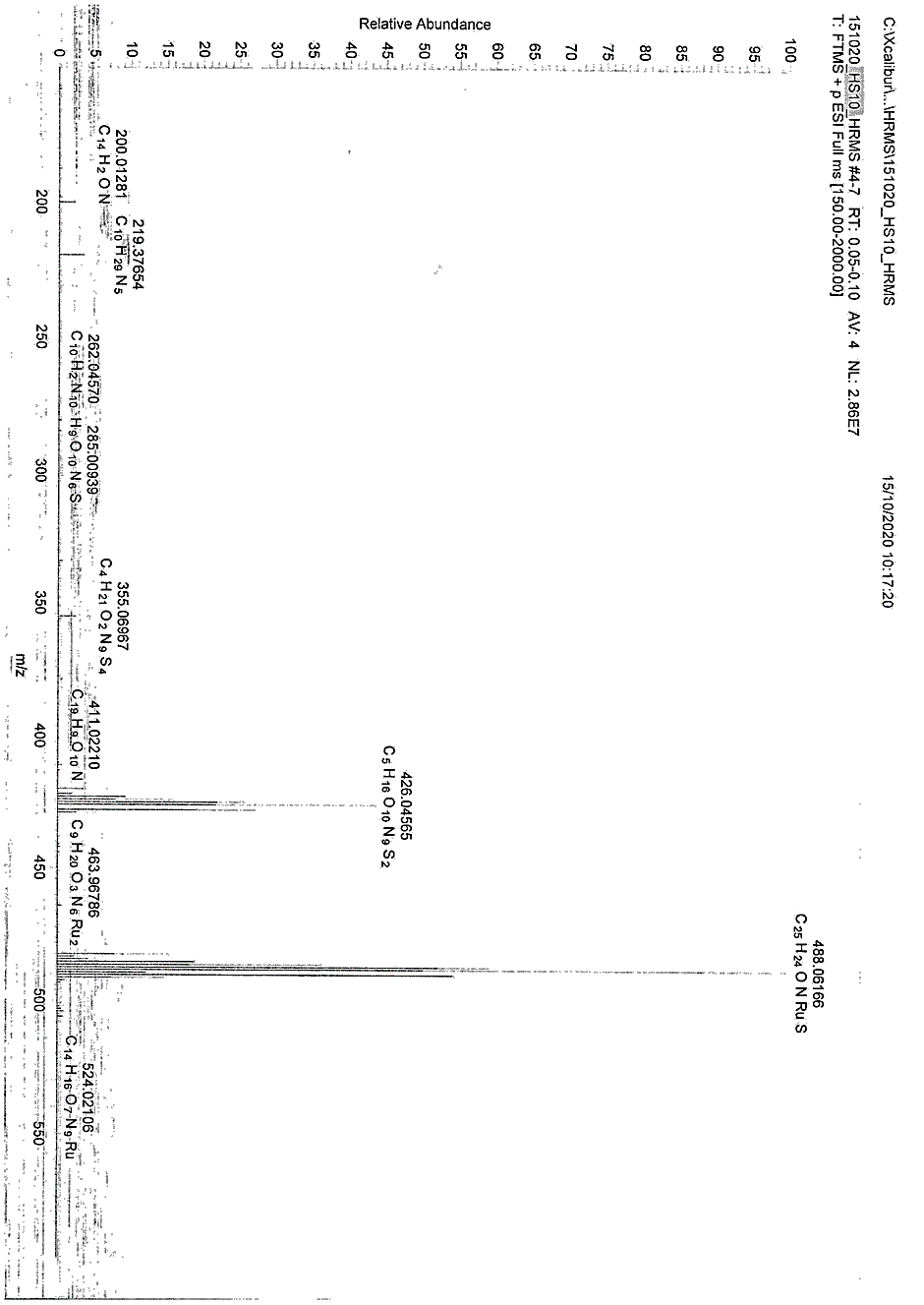


Figure S36. HRMS spectrum of complex **4**


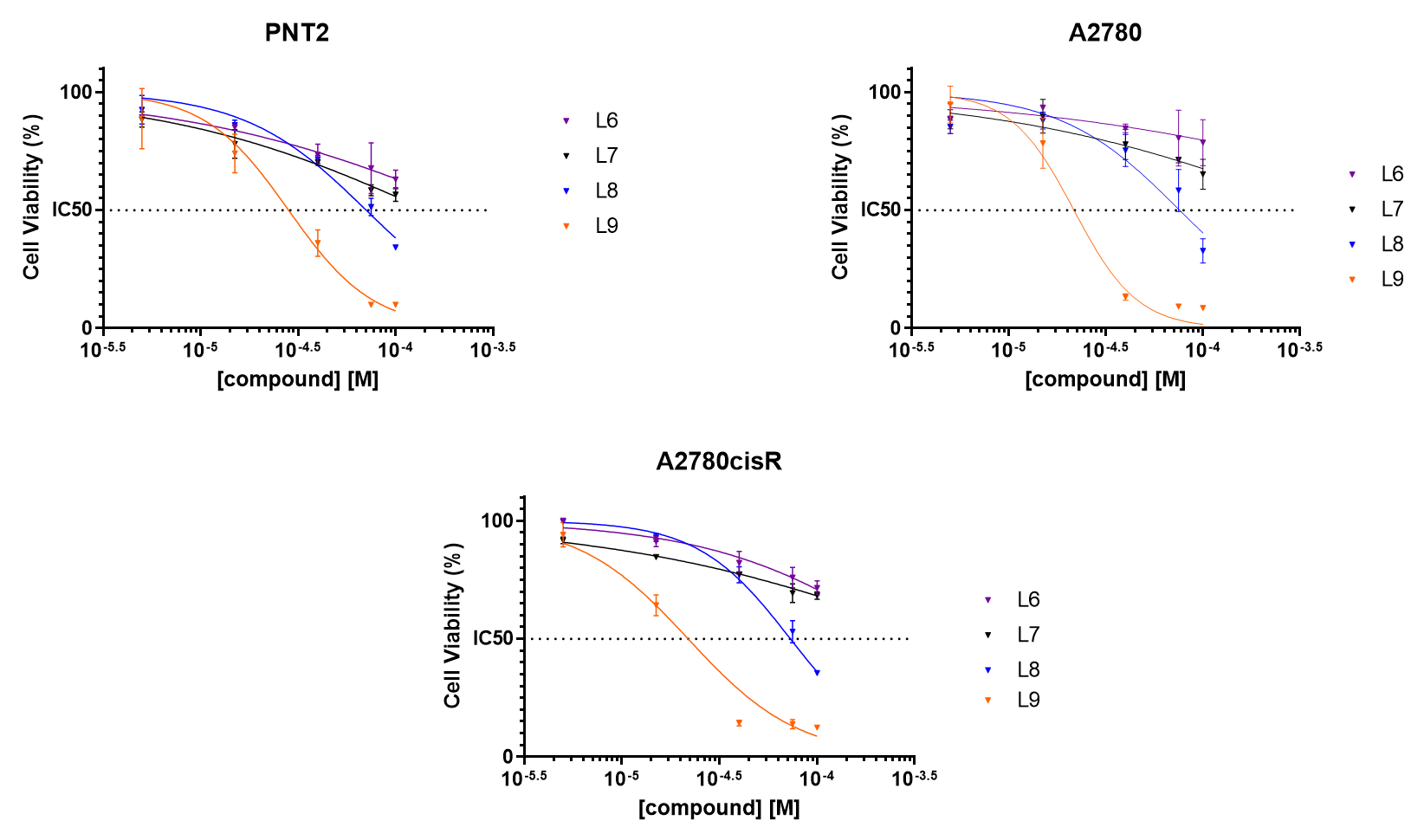


Figure S38. IC_50_ graphs for ligands L6 – L9 against PNT2, A2780, and A2780cisR


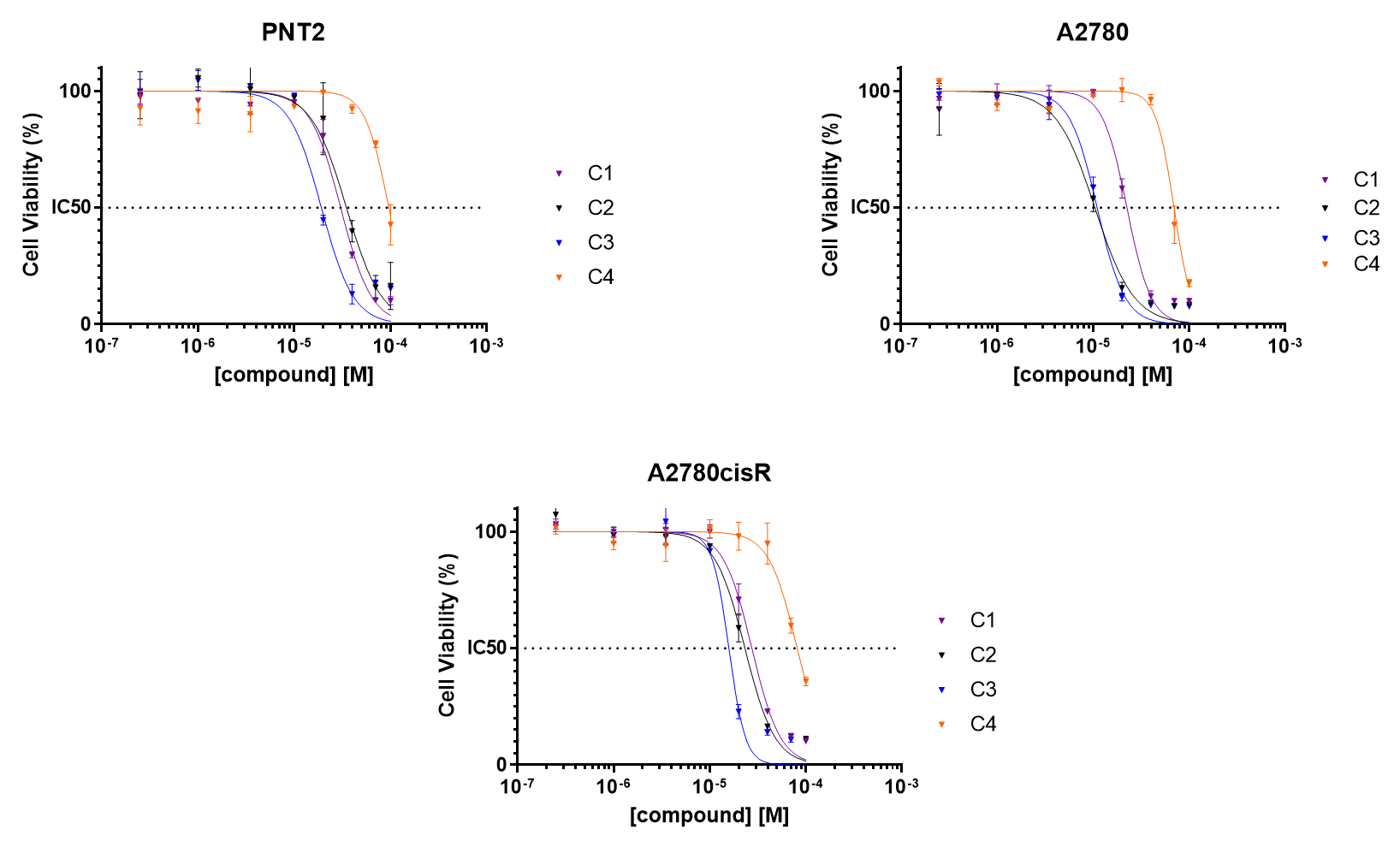


Figure S38. IC_50_ graphs for complexes **1** – **4** against PNT2, A2780, and A2780cisR


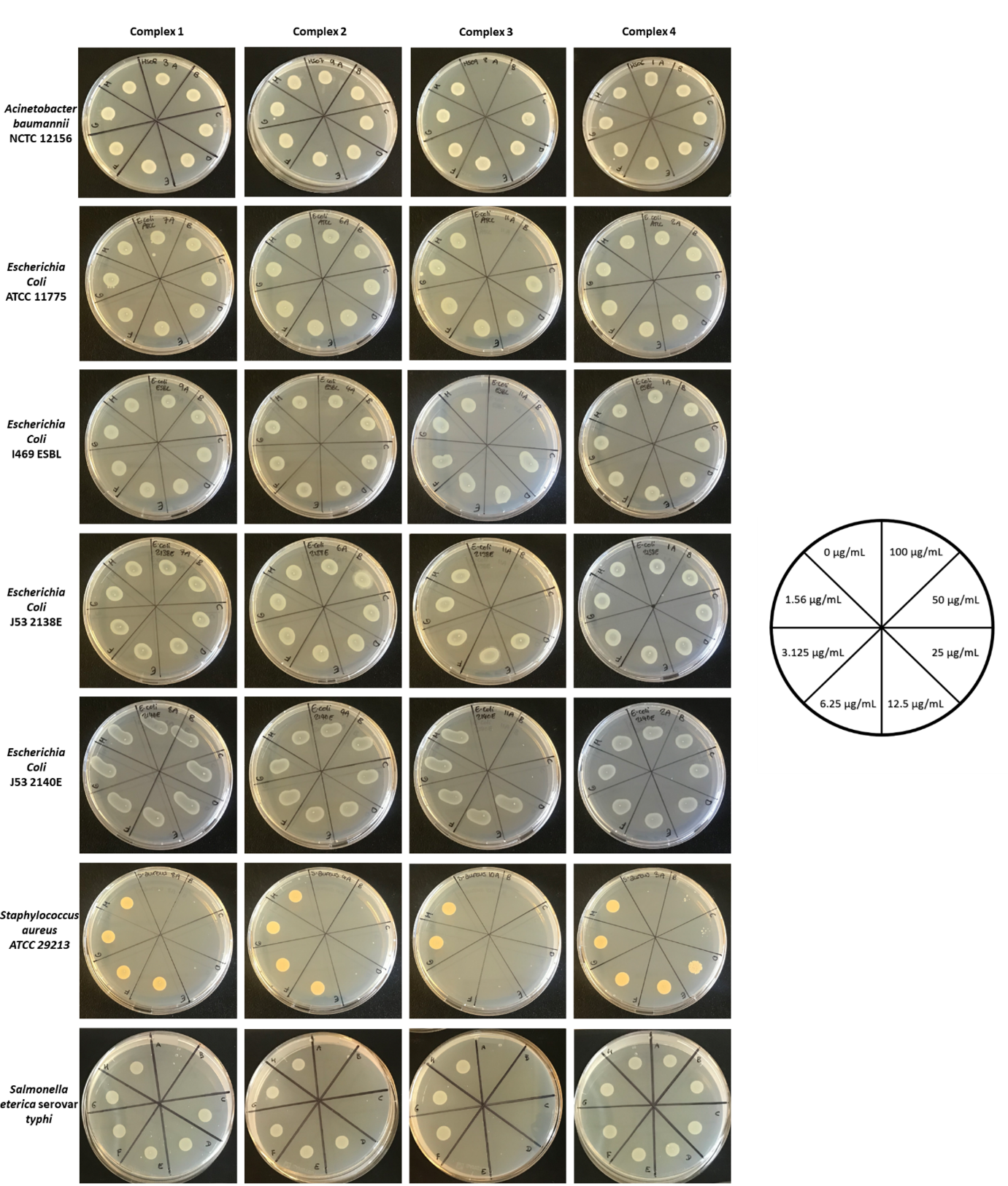


Figure S39. Minimum bactericidal concentrations for Complexes **1-4**. The organisms that were observed to have bactericidal activity were *Escherichia coli* ATCC 11775, *E. coli* I469 ESBL, *E. coli* J53 2138E, *E. coli* J53 2140E, *Staphylococcus aureus* ATCC29213 and *Salmonella enterica* serovar *typhi*. Complex **1** had a minimum bactericidal concentration (MBC) of 12.5 µg/mL for *S. aureus* and 50 µg/mL for *S. typhi.* No MBC was observed for any *E. coli* strain tested. Complex **2** had an MBC of 12.5 µg/mL for *S. aureus* and 25 µg/mL for *S. typhi.* No MBC was observed for any *E. coli* strain tested. Complex **3** had MBCs of 50 µg/mL against both *E. coli* ATCC 11775 and *E. coli* I469 ESBL, 25 µg/mL for *E. coli* J53 2138E and *E. coli* 2140E, 6.25 µg/mL for *S. typhi* and 3.125 µg/mL for *S. aureus*. Complex **4** only had an MBC observed for *S. aureus* of 50 µg/mL where single colonies can be observed.


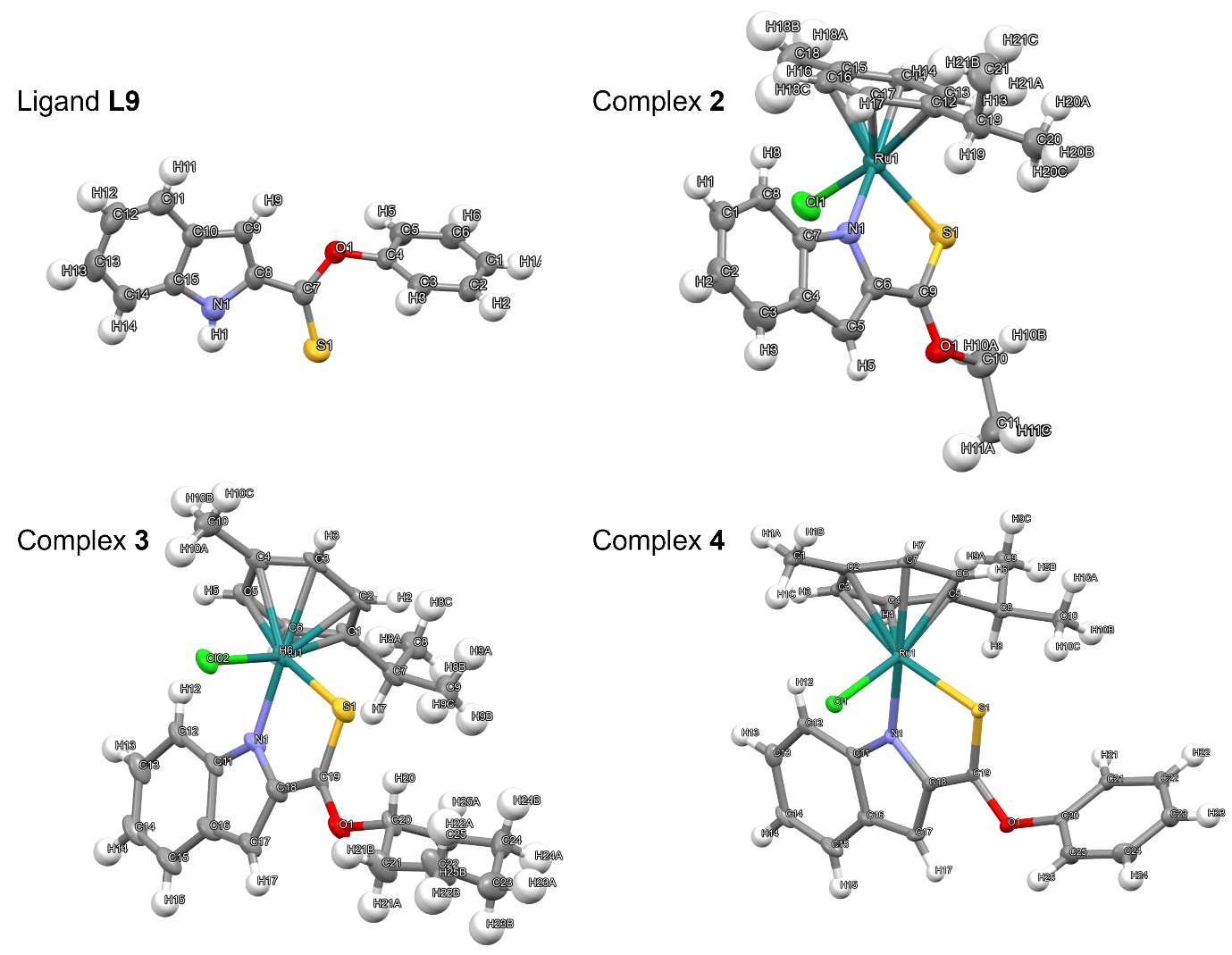


Figure S40. X-ray structures of ligand **L9** and complexes **2-4**. Thermal ellipsoids are drawn at the 50% probability level.

Table S1. Crystal data and structure refinement for ligand **L9**

| Identification code | Ligand **L9** |
| --- | --- |
| Empirical formula | C_15_H_11_NOS |
| Formula weight | 253.31 |
| Temperature (K) | 296.15 |
| Crystal system | orthorhombic |
| Space group | Pbca |
| a (Å) | 6.0376(2) |
| b (Å) | 8.1016(3) |
| c (Å) | 50.6945(18) |
| α (°) | 90 |
| β (°) | 90 |
| γ (°) | 90 |
| Volume (Å^3^) | 2479.68(15) |
| Z | 8 |
| ρ_calc_ (g/cm^3^) | 1.357 |
| μ (mm^‑1^) | 0.246 |
| F(000) | 1056.0 |
| Crystal size (mm^3^) | 0.75 × 0.51 × 0.24 |
| Radiation | MoKα (λ = 0.71073) |
| 2Θ range for data collection (°) | 4.822 to 52.874 |
| Index ranges | -7 ≤ h ≤ 7, -10 ≤ k ≤ 10, -63 ≤ l ≤ 63 |
| Reflections collected | 27809 |
| Independent reflections | 2555 [R_int_ = 0.0406, R_sigma_ = 0.0203] |
| Data/restraints/parameters | 2555/0/163 |
| Goodness-of-fit on F^2^ | 1.186 |
| Final R indexes [I>=2σ (I)] | R_1_ = 0.0533, wR_2_ = 0.1107 |
| Final R indexes [all data] | R_1_ = 0.0661, wR_2_ = 0.1159 |
| Largest diff. peak/hole (e Å^-3^) | 0.21/-0.28 |

Table S2. Selected bond lengths (Å) for ligand **L9**

| Atoms | Length (Å) |
| --- | --- |
| S1-C7 | 1.619(3) |
| O1-C4 | 1.407(3) |
| O1-C7 | 1.360(3) |
| N1-C15 | 1.384(3) |
| N1-C8 | 1.368(3) |
| C9-C10 | 1.405(3) |
| C9-C8 | 1.355(3) |
| C10-C15 | 1.406(3) |
| C10-C11 | 1.404(4) |
| C4-C5 | 1.369(3) |
| C4-C3 | 1.371(3) |
| C15-C14 | 1.394(3) |
| C8-C7 | 1.448(3) |
| C5-C6 | 1.386(4) |
| C3-C2 | 1.379(3) |
| C1-C2 | 1.375(4) |
| C1-C6 | 1.371(4) |
| C14-C13 | 1.366(4) |
| C11-C12 | 1.368(4) |
| C13-C12 | 1.392(4) |

Table S3. Selected bond angles (°) for ligand **L9**

| Atoms | Angle (˚) |
| --- | --- |
| C7-O1-C4 | 119.98(19) |
| C8-N1-C15 | 109.0(2) |
| C8-C9-C10 | 108.2(2) |
| C9-C10-C15 | 107.0(2) |
| C11-C10-C9 | 133.8(2) |
| C11-C10-C15 | 119.2(2) |
| C5-C4-O1 | 117.0(2) |
| C5-C4-C3 | 122.1(2) |
| C3-C4-O1 | 120.7(2) |
| N1-C15-C10 | 106.8(2) |
| N1-C15-C14 | 131.7(2) |
| C14-C15-C10 | 121.6(2) |
| N1-C8-C7 | 123.0(2) |
| C9-C8-N1 | 109.0(2) |
| C9-C8-C7 | 127.9(2) |
| O1-C7-S1 | 125.63(18) |
| O1-C7-C8 | 109.4(2) |
| C8-C7-S1 | 125.0(2) |
| C4-C5-C6 | 118.6(2) |
| C4-C3-C2 | 118.4(2) |
| C6-C1-C2 | 119.9(2) |
| C1-C2-C3 | 120.6(3) |
| C13-C14-C15 | 117.7(3) |
| C12-C11-C10 | 118.5(3) |
| C1-C6-C5 | 120.3(3) |
| C14-C13-C12 | 121.6(3) |
| C11-C12-C13 | 121.4(3) |

Table S4. Crystal data and structure refinement for complex **2**

| Identification code | Complex **2** |
| --- | --- |
| Empirical formula | C_21_H_24_ClNORuS |
| Formula weight | 474.99 |
| Temperature (K) | 276.15 |
| Crystal system | monoclinic |
| Space group | P2_1_/n |
| a (Å) | 14.200(5) |
| b (Å) | 9.637(3) |
| c (Å) | 14.732(6) |
| α (°) | 90 |
| β (°) | 100.752(14) |
| γ (°) | 90 |
| Volume (Å^3^) | 1980.7(13) |
| Z | 4 |
| ρ_calc_ (g/cm^3^) | 1.593 |
| μ (mm^‑1^) | 1.042 |
| F(000) | 968.0 |
| Crystal size (mm^3^) | 0.53 × 0.26 × 0.21 |
| Radiation | MoKα (λ = 0.71073) |
| 2Θ range for data collection (°) | 5.078 to 56.782 |
| Index ranges | -18 ≤ h ≤ 17, -12 ≤ k ≤ 12, -19 ≤ l ≤ 19 |
| Reflections collected | 14345 |
| Independent reflections | 4881 [R_int_ = 0.0345, R_sigma_ = 0.0461] |
| Data/restraints/parameters | 4881/0/316 |
| Goodness-of-fit on F^2^ | 1.013 |
| Final R indexes [I>=2σ (I)] | R_1_ = 0.0330, wR_2_ = 0.0643 |
| Final R indexes [all data] | R_1_ = 0.0550, wR_2_ = 0.0727 |
| Largest diff. peak/hole (e Å^-3^) | 0.40/-0.54 |

Table S5. Selected bond lengths (Å) for Complex **2**

| Atoms | Length (Å) |
| --- | --- |
| Ru1-Cl1 | 2.4073(10) |
| Ru1-S1 | 2.3813(11) |
| Ru1-N1 | 2.084(2) |
| Ru1-C12 | 2.209(3) |
| Ru1-C13 | 2.166(3) |
| Ru1-C14 | 2.209(3) |
| Ru1-C15 | 2.249(3) |
| Ru1-C16 | 2.221(3) |
| Ru1-C17 | 2.173(3) |
| S1-C9 | 1.672(3) |
| O1-C9 | 1.322(3) |
| O1-C10 | 1.440(4) |
| N1-C6 | 1.371(3) |
| N1-C7 | 1.359(3) |

Table S6. Selected bond angles (°) for Complex **2**

| Atoms | Angle (˚) |
| --- | --- |
| S1-Ru1-Cl1 | 85.11(3) |
| N1-Ru1-Cl1 | 84.42(7) |
| N1-Ru1-S1 | 81.06(6) |
| N1-Ru1-C12 | 104.32(9) |
| N1-Ru1-C13 | 137.83(10) |
| N1-Ru1-C14 | 171.16(10) |
| N1-Ru1-C15 | 139.73(10) |
| N1-Ru1-C16 | 107.12(10) |
| N1-Ru1-C17 | 92.35(10) |
| C12-Ru1-C11 | 169.20(8) |
| C12-Ru1-S1 | 102.34(8) |
| C12-Ru1-C14 | 68.13(11) |
| O1-C9-S1 | 124.5(2) |
| C13-Ru1-S1 | 89.53(9) |
| C14-Ru1-S1 | 104.68(9) |
| C15-Ru1-S1 | 137.78(8) |
| C16-Ru1-S1 | 168.31(8) |
| C17-Ru1-S1 | 136.27(8) |
| O1-C10-C11 | 108.0(3) |

Table S7. Crystal data and structure refinement for complex **3**

| Identification code | Complex **3** |
| --- | --- |
| Empirical formula | C_25_H_30_ClNORuS |
| Formula weight | 529.08 |
| Temperature (K) | 100.05 |
| Crystal system | monoclinic |
| Space group | P2_1_/c |
| a (Å) | 13.5831(15) |
| b (Å) | 9.3045(11) |
| c (Å) | 18.0026(18) |
| α (°) | 90 |
| β (°) | 98.045(6) |
| γ (°) | 90 |
| Volume (Å^3^) | 2252.8(4) |
| Z | 4 |
| ρ_calc_ (g/cm^3^) | 1.560 |
| μ (mm^‑1^) | 0.925 |
| F(000) | 1088.0 |
| Crystal size (mm^3^) | 0.495 × 0.213 × 0.076 |
| Radiation | MoKα (λ = 0.71073) |
| 2Θ range for data collection (°) | 4.57 to 56.722 |
| Index ranges | -18 ≤ h ≤ 18, -10 ≤ k ≤ 12, -24 ≤ l ≤ 24 |
| Reflections collected | 34003 |
| Independent reflections | 5608 [R_int_ = 0.1167, R_sigma_ = 0.0944] |
| Data/restraints/parameters | 5608/0/274 |
| Goodness-of-fit on F^2^ | 1.020 |
| Final R indexes [I>=2σ (I)] | R_1_ = 0.0583, wR_2_ = 0.1322 |
| Final R indexes [all data] | R_1_ = 0.1086, wR_2_ = 0.1553 |
| Largest diff. peak/hole (e Å^-3^) | 1.90/-2.04 |

Table S8. Selected bond lengths (Å) for Complex **3**

| Atoms | Length (Å) |
| --- | --- |
| Ru1-Cl2 | 2.4117(14) |
| Ru1-S1 | 2.3723(14) |
| Ru1-N1 | 2.088(4) |
| Ru1-C1 | 2.195(6) |
| Ru1-C2 | 2.168(5) |
| Ru1-C3 | 2.221(5) |
| Ru1-C4 | 2.251(5) |
| Ru1-C5 | 2.220(5) |
| Ru1-C6 | 2.172(5) |
| S1-C19 | 1.668(5) |
| O1-C19 | 1.327(6) |
| O1-C20 | 1.470(5) |
| N1-C11 | 1.359(6) |
| N1-C18 | 1.365(7) |

Table S9. Selected bond angles (°) for Complex **3**

| Atoms | Angle (˚) |
| --- | --- |
| S1-Ru1-Cl2 | 85.32(5) |
| N1-Ru1-Cl2 | 84.77(13) |
| N1-Ru1-S1 | 81.30(12) |
| N1-Ru1-C1 | 103.54(18) |
| N1-Ru1-C2 | 137.14(19) |
| N1-Ru1-C3 | 171.11(18) |
| N1-Ru1-C4 | 140.53(17) |
| N1-Ru1-C5 | 107.29(17) |
| N1-Ru1-C6 | 92.08(18) |
| C1-Ru1-Cl2 | 170.02(15) |
| C1-Ru1-S1 | 101.25(14) |
| C1-Ru1-C3 | 68.53(19) |
| O1-C19-S1 | 124.1(4) |
| C2-Ru1-S1 | 88.25(14) |
| C3-Ru1-S1 | 103.83(13) |
| C4-Ru1-S1 | 136.96(13) |
| C5-Ru1-S1 | 167.71(14) |
| C6-Ru1-S1 | 135.97(14) |
| O1-C20-C21 | 107.4(4) |

Table S10. Crystal data and structure refinement for complex 4

| Identification code | Complex 4 |
| --- | --- |
| Empirical formula | C_25_H_24_ClNORuS |
| Formula weight | 523.03 |
| Temperature (K) | 105.15 |
| Crystal system | monoclinic |
| Space group | P2_1_/c |
| a (Å) | 13.1367(9) |
| b (Å) | 9.2851(6) |
| c (Å) | 17.9816(13) |
| α (°) | 90 |
| β (°) | 101.507(4) |
| γ (°) | 90 |
| Volume (Å^3^) | 2149.2(3) |
| Z | 4 |
| ρ_calc_ (g/cm^3^) | 1.616 |
| μ (mm^‑1^) | 0.969 |
| F(000) | 1064.0 |
| Crystal size (mm^3^) | 0.202 × 0.169 × 0.088 |
| Radiation | MoKα (λ = 0.71073) |
| 2Θ range for data collection (°) | 4.958 to 55.244 |
| Index ranges | -17 ≤ h ≤ 16, -12 ≤ k ≤ 11, -22 ≤ l ≤ 22 |
| Reflections collected | 14049 |
| Independent reflections | 4715 [R_int_ = 0.0477, R_sigma_ = 0.0657] |
| Data/restraints/parameters | 4715/0/274 |
| Goodness-of-fit on F^2^ | 1.021 |
| Final R indexes [I>=2σ (I)] | R_1_ = 0.0370, wR_2_ = 0.0676 |
| Final R indexes [all data] | R_1_ = 0.0611, wR_2_ = 0.0764 |
| Largest diff. peak/hole (e Å^-3^) | 0.63/-0.60 |

Table S11. Selected bond lengths (Å) for Complex **4**

| Atoms | Length (Å) |
| --- | --- |
| Ru1-Cl1 | 2.4059(8) |
| Ru1-S1 | 2.3812(8) |
| Ru1-N1 | 2.082(3) |
| Ru1-C2 | 2.256(3) |
| Ru1-C3 | 2.224(3) |
| Ru1-C4 | 2.174(3) |
| Ru1-C5 | 2.205(3) |
| Ru1-C6 | 2.173(3) |
| Ru1-C7 | 2.228(3) |
| S1-C19 | 1.670(3) |
| O1-C19 | 1.356(4) |
| O1-C20 | 1.405(4) |
| N1-C11 | 1.351(4) |
| N1-C18 | 1.389(4) |

Table S12. Selected bond angles (°) for Complex **4**

| Atoms | Angle (˚) |
| --- | --- |
| S1-Ru1-Cl1 | 85.73(3) |
| N1-Ru1-Cl1 | 85.27(7) |
| N1-Ru1-S1 | 81.28(7) |
| N1-Ru1-C2 | 139.51(11) |
| N1-Ru1-C3 | 106.54(11) |
| N1-Ru1-C4 | 91.21(11) |
| N1-Ru1-C5 | 103.35(11) |
| N1-Ru1-C6 | 137.03(11) |
| N1-Ru1-C7 | 170.22(10) |
| C2-Ru1-Cl1 | 88.68(8) |
| C2-Ru1-S1 | 138.14(9) |
| C3-Ru1-Cl1 | 103.63(8) |
| O1-C19-S1 | 124.0(2) |
| C3-Ru1-S1 | 168.08(8) |
| C4-Ru1-S1 | 135.61(9) |
| C5-Ru1-S1 | 101.78(9) |
| C6-Ru1-S1 | 89.04(9) |
| C7-Ru1-S1 | 104.97(8) |
| O1-C19-C18 | 114.7(3) |
